# Supplementary material for: Nivolumab plus platinum-doublet chemotherapy in treatment-naive patients with advanced grade 3 Neuroendocrine Neoplasms of gastroenteropancreatic or unknown origin: The multicenter phase 2 NICE-NEC trial (GETNE-T1913)
Source: Nat Commun. 2024 Aug 8;15:6753. doi: 10.1038/s41467-024-50969-8 (PMC11310219; doi:10.1038/s41467-024-50969-8)
Supplement: Supplementary file 1 — Supplementary Information [file 41467_2024_50969_MOESM1_ESM.pdf]

SUPPLEMENTARY INFORMATION

Supplementary Table 1. Immune-related adverse events

**Event; n (%)**	**G3 GEP-NENs; N = 38**	
	**Any grade**	**Grade ≥3**
Oral mucositis	6 (15.8)	1 (2.6)
Pruritus	4 (10.5)	-
Rash	2 (5.3)	-
Hypothyroidism	3 (7.9)	-
Hyperthyroidism	2 (5.3)	-
Arthralgia	2 (5.3)	-
Skin hyperpigmentation	1 (2.6)	-
Acute kidney injury	1 (2.6)	1 (2.6)
AST increased	2 (5.3)	-
ALT increased	2 (5.3)	1 (2.6)

Supplementary Table 2. Treatment-related adverse events observed in >5% of patients.

AEs; n (%)	All	G-1	G-2	G-3	G-4	G-5
**Fatigue**	23 (60.5)	8 (21.1)	12 (31.6)	3 (7.9)	-	-
**Neutropenia**	21 (55.3)	2 (5.3)	4 (10.5)	10 (26.3)	5 (13.2)	-
**Nausea**	17 (44.7)	11 (29)	6 (15.8)	-	-	-
**Anemia**	12 (31.6)	4 (10.5)	5 (13.2)	3 (7.9)	-	-
**Alopecia**	12 (31.6)	3 (7.9)	9 (23.7)	-	-	-
**Diarrhea**	11 (29)	8 (21.1)	3 (7.9)	-	-	-
**Vomiting**	8 (21.1)	6 (15.8)	2 (5.3)	-	-	-
**Thrombopenia**	8 (21.1)	4 (10.5)	2 (5.3)	1 (2.6)	1 (2.6)	-
**Anorexia**	7 (18.4)	2 (5.3)	5 (13.2)	-	-	-
**Oral mucositis**	6 (15.8)	4 (10.5)	1 (2.6)	1 (2.6)	-	-
**Gastrointestinal disorders**	5 (13.2)	4 (10.5)	-	-	-	-
**Constipation**	5 (13.2)	3 (7.9)	2 (5.3)	-	-	-
**Febrile neutropenia**	4 (10.5)	-	-	1 (2.6)	3 (7.9)	-
**Pruritus**	4 (10.5)	4 (10.5)	-	-	-	-
**Dysgeusia**	3 (7.9)	3 (7.9)	-	-	-	-
**Leucopenia**	3 (7.9)	2 (5.3)	1 (2.6)	-	-	-
**Fever**	3 (7.9)	3 (7.9)	-	-	-	-
**Hypothyroidism**	3 (7.9)	-	3 (7.9)	-	-	-
**Arthralgia**	2 (5.3)	-	2 (5.3)	-	-	-
**Hyperthyroidism**	2 (5.3)	-	2 (5.3)	-	-	-
**Acneiform rash**	2 (5.3)	2 (5.3)	-	-	-	-
**Hypomagnesemia**	2 (5.3)	2 (5.3)	-	-	-	-
**ALT increased**	2 (5.3)	1 (2.6)	-	1 (2.6)	-	-
**AST increased**	2 (5.3)	1 (2.6)	1 (2.6)	-	-	-
**Renal and urinary disorders**	2 (5.3)	1 (2.6)	1 (2.63)	-	-	-
**Esophageal mucositis**	1 (2.6)	-	-	-	-	1 (2.6)

Supplementary Table 3. Antibodies used to assess PD-L1 and MMR protein expression by immunohistochemistry.

PROTEIN	CLON	DILUTION	COMPANY	COUNTRY	REFERENCE
MLH1	MLH1 E505	Prediluted, ready to use	LEICA	Germany	PA0988
MSH2	MSH2 79H11	Prediluted, ready to use	LEICA	Germany	PA0989
MSH6	MSH6 EP49	Prediluted, ready to use	LEICA	Germany	PA0990
PMS2	PMS2 EP 51	Prediluted, ready to use	LEICA	Germany	PA091
PDL1	22C3 PharmDx	Prediluted, ready to use	DAKO	USA	Ref SK 006

Further information may be found in:
https://www.leicabiosystems.com/en-es/products/
https://www.agilent.com/en/dako-products

Supplementary figure 1. Stratified analysis of OS. A) Stratified analysis of OS according to Ki-67 index (A), differentiation (NET vs NEC) (B), CgA levels at baseline (C), enolase levels at baseline (D), and primary tumor site (E). Kaplan-Meier comparisons between subgroups are calculated by the log-rank test. Subgroup analyses were pre-planned to explore potential signs of differential efficacy by most relevant clinical features, although the study was not powered for formal comparisons; therefore, all findings are exploratory in nature. The solid lines show the estimated survival proportions of each subgroup and the corresponding coloured shadow areas the 95% CI. The red dashed lines indicate the 50% survival probability. Abbreviations: CGA, chromogranin A; NEC, neuroendocrine carcinoma; NET, neuroendocrine tumor; NSE, neuron-specific enolase; OS, overall survival.

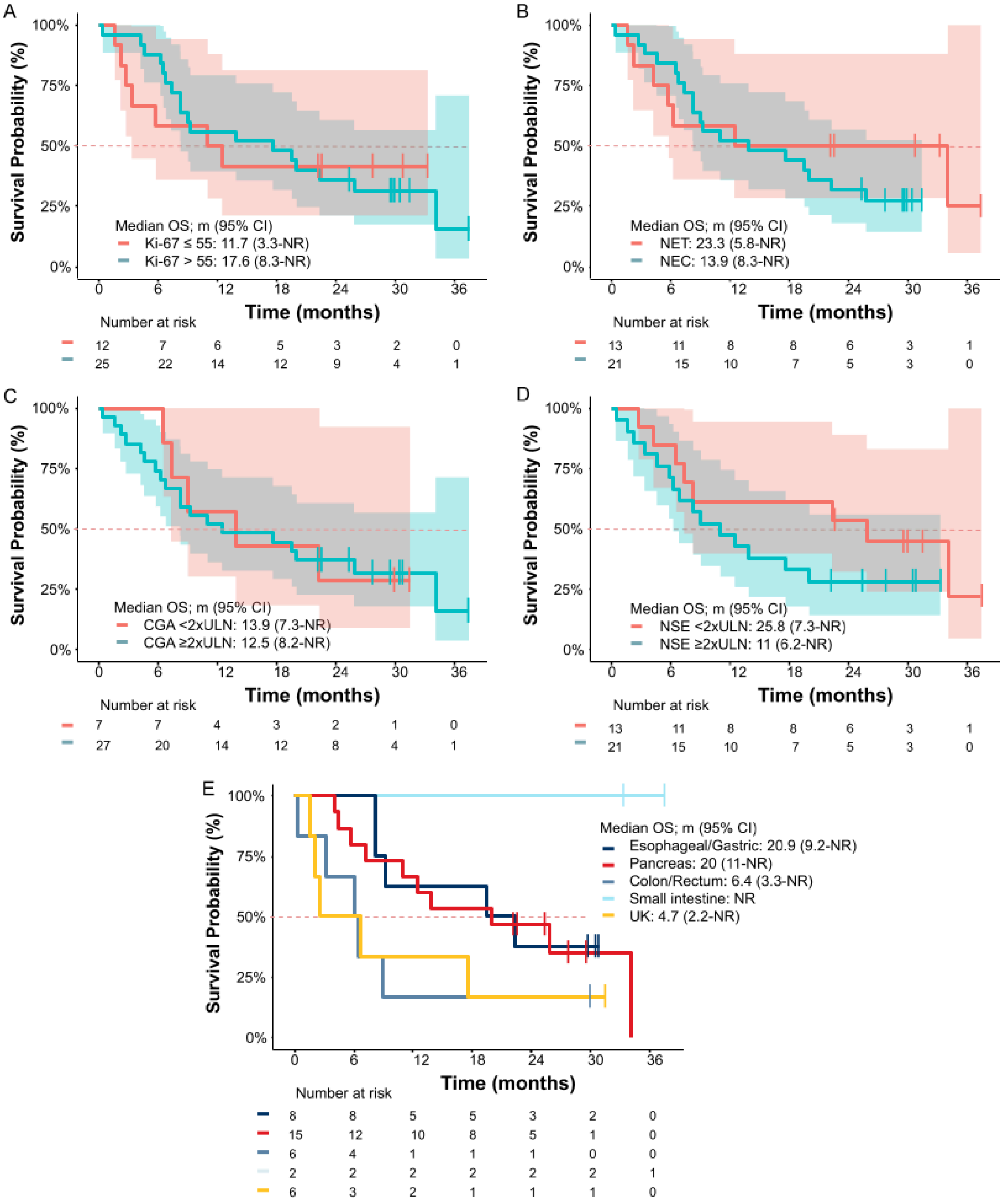

Supplementary figure 2. Univariable forest plot analysis of OS. The graph shows all baseline characteristics tested in univariable analysis and used for the construction of multivariable Cox models. These subgroup analyses were pre-planned but were exploratory in nature as the study was not powered for formal comparisons. The forest plot shows the hazard ratio of each subgroup and its 95% CI. Abbreviations: CGA, chromogranin A; CI, confidence interval; ECOG, Eastern cooperative oncology group performance status; HR, hazard ratio; LDH, lactate dehydrogenase; NSE, neuron specific enolase; y, years.

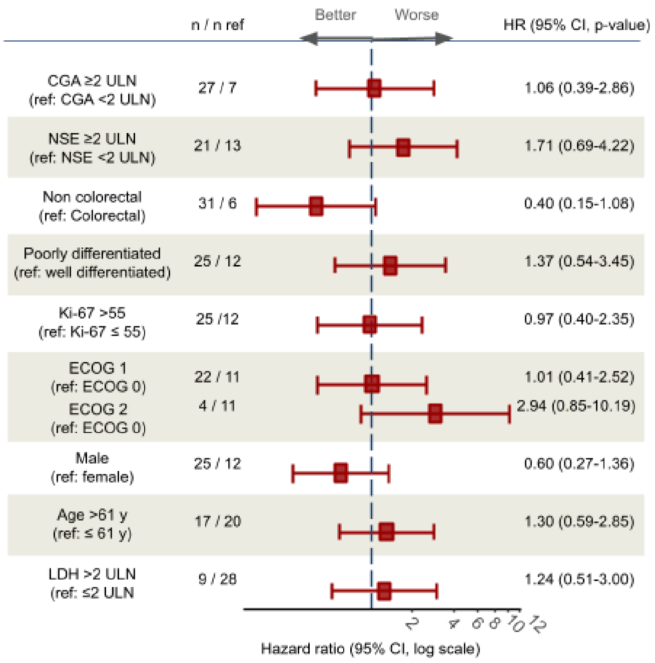

Supplementary Figure 3. Stratified analysis of PFS. A) Stratified analysis of PFS according to Ki-67 index (A), tumor differentiation (NET vs NEC) (B), baseline CgA levels (C), baseline enolase levels (D), and primary tumor site (E). Comparisons between subgroups were assessed by the log-rank test. Subgroup analyses were pre-planned but were exploratory in nature as the study was not powered for formal comparisons. The solid lines show the estimated survival proportions of each subgroup and the corresponding coloured shadow areas the 95% CI. The red dashed lines indicate the 50% survival probability. Abbreviations: CGA, chromogranin A; NEC, neuroendocrine carcinoma; NET, neuroendocrine tumor; NSE, neuron-specific enolase; PFS, progression-free survival; OS, overall survival.

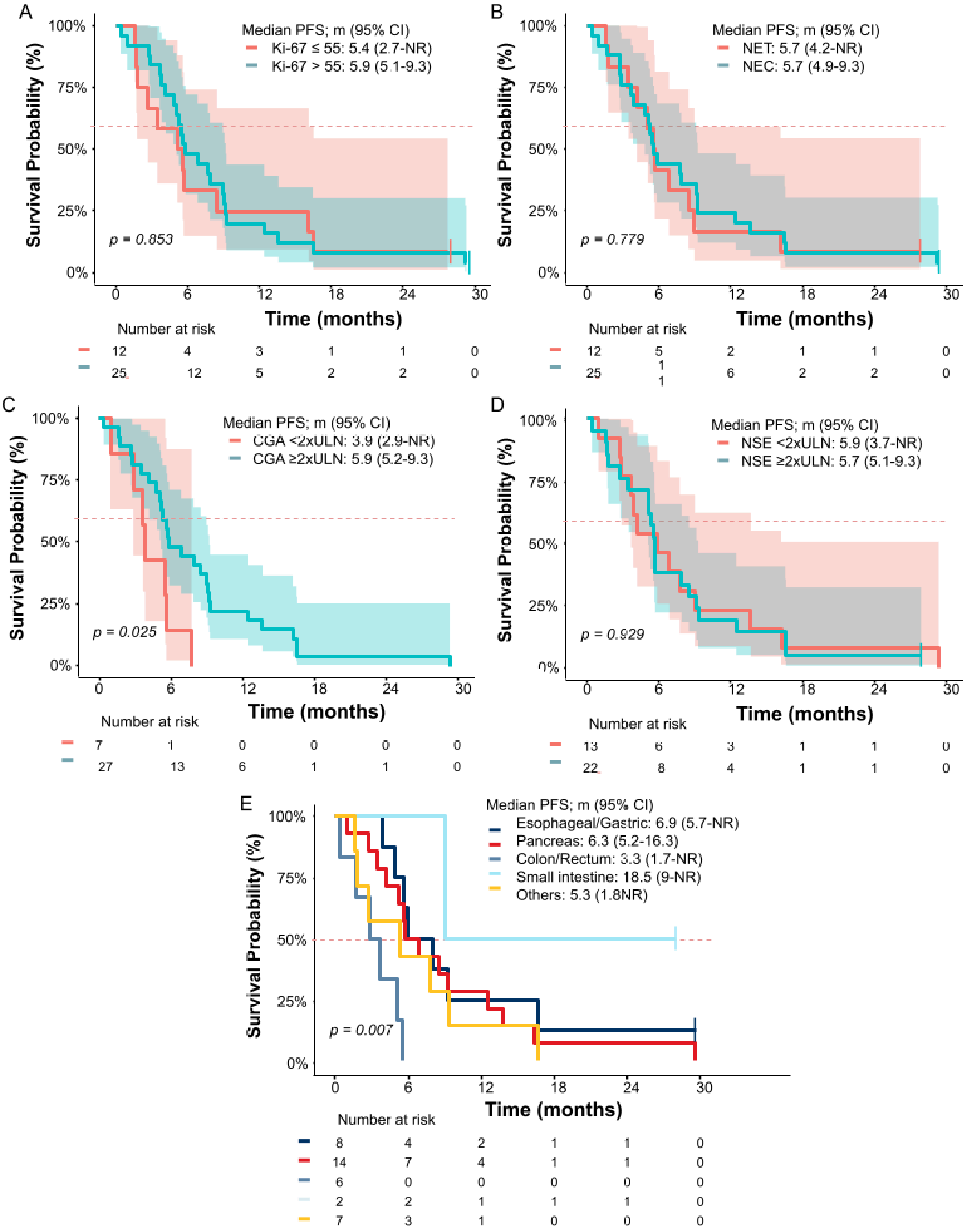

Supplementary figure 4. Univariable forest plot analysis of PFS. The graph shows all baseline characteristics tested in univariable analysis and used for the construction of multivariable Cox models. These subgroup analyses were pre-planned but were exploratory in nature as the study was not powered for formal comparisons.. The forest plot shows the hazard ratio of each subgroup and its 95% CI. Abbreviations: CGA, chromogranin A; CI, confidence interval; ECOG, Eastern cooperative oncology group performance status; HR, hazard ratio; LDH, lactate dehydrogenase; NSE, neuron specific enolase; y, years.

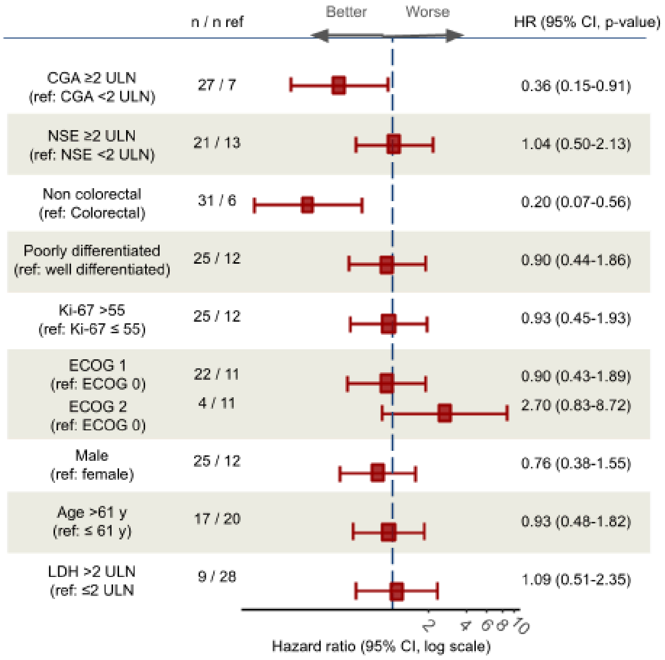

SUPPLEMENTARY NOTE

A phase II study of Platinum-doublet chemotherapy in combination with nivolumab as first-line treatment, in subjects with unresectable, locally advanced or metastatic G3 Neuroendocrine Neoplasms (NENs) of the gastroenteropancreatic (GEP) tract or of unknown (UK) origin.

Protocol identification number / code / Registration n°: CA209-73D

Sponsor code: GETNE-T1913

EudraCT number: 2019-001546-18

Acronym: NICE-NEC

Study Coordinator or Chair:

Rocío García-Carbonero, MD, PhD

Maria del Carmen Riesco-Martínez, MD, PhD

[redacted]

(date)	**Protocol approval**
(date)	**Amendment x**

Version: (3.1) / 17 June, 2019

SYNOPSIS

Clinical Protocol CA209- 73D

Sponsor code: GETNE-T1913

EudraCT number: 2019-001546-18

Protocol Title: A phase II study of platinum-doublet chemotherapy in combination with nivolumab as first-line treatment in subjects with unresectable, locally advanced or metastatic G3 neuroendocrine neoplasms (NENs) of the gastroenteropancreatic (GEP) tract or of unknown (UK) origin.

Sponsor: Grupo Español de Tumores Neuroendocrinos y Endocrinos (GETNE)

Investigational Product(s), Dose and Mode of Administration, Duration of Treatment with Investigational Product(s):

INDUCTION PHASE

- Nivolumab 360 mg IV will be administered every 3 weeks (±3 days)
- Carboplatin (AUC=5) IV will be administered every 3 weeks (±3 days) following the administration of nivolumab.
- Etoposide 100 mg/m^2/day IV will be administered on D1-3 every 3 weeks (±3 days) following the administration of carboplatin.

MAINTENANCE PHASE

- Nivolumab 480 mg IV will be administered every 4 weeks (±3 days) for 2 years

Study Phase: Non-randomized phase II

Study Population:

- Patients with histologically confirmed non-resectable or stage IV G3 (Ki-67>20%) neuroendocrine neoplasms of the GEP tract or of UK origin.
- No prior systemic therapy.
- Available tumor sample for translational research.

Research Hypothesis:

- The addition of Nivolumab to platinum-based chemotherapy doublet will induce deeper and more durable tumor responses and prolong overall survival (OS) of patients with advanced G3 NENs of gastroenteropancreatic (GEP) or unknown (UK) origin, as compared to historical cohorts of patients treated with standard chemotherapy alone.

Objectives:

Objectives	Endpoints
**Primary**<br>● To determine the overall survival patients with advanced G3 NENs treated with nivolumab + platinum-based chemotherapy.	● 1 year-OS rate
**Secondary**<br>● To determine other efficacy outcomes of nivolumab + platinum-based chemotherapy in patients with advanced G3 NENs.	● ORR<br>● Duration of response<br>● PFS<br>● OS
● To evaluate the safety and tolerability of nivolumab + platinum-based chemotherapy in this patient population.	● Incidence of AEs, SAEs and selected AEs
● To evaluate biochemical response as predictive biomarker of efficacy of nivolumab + chemotherapy in this patient population.	● Chromogranin A and enolase values and their association with ORR, PFS and OS.
● To explore potential predictive and prognostic biomarkers.	● Mutational burden, gene expression signature, soluble factors and other molecular markers in peripheral blood and their association with clinical outcomes.

Study Design:

Protocol **CA209- 73D** is a non-randomized, open-label, phase 2 trial that will enroll 38 ≥ 18 years old with untreated metastatic or unresectable G3 NENs of gastroenteropancreatic or UK origin, evaluating the efficacy of nivolumab combined with platinum-based doublet chemotherapy as first-line treatment, followed by maintenance treatment with nivolumab in patients who have not progressed after 6 months of first line platinum-based induction chemotherapy.

Tumor progression and response endpoints will be assessed using RECIST 1.1 criteria. If there is no evidence of disease progression after the induction phase, treatment with nivolumab will continue until RECIST 1.1 defined progression or death, unacceptable toxicity, 24 months of treatment or withdrawal of consent.

Dose reductions will not be allowed for nivolumab. Treatment beyond initial investigator-assessed progression (either clinical or radiological) is permitted for nivolumab if the subject has an investigator-assessed clinical benefit and is tolerating well study drug.

The study period will include 18 months of accrual plus 12 months of follow-up. The study will end once survival follow-up has concluded.

Study Treatment

Medication	Potency	IP/Non-IP
Nivolumab	100 mg/vial	IP
Carboplatin	450 mg/vial	IP
Etoposide	100 mg/vial	IP

Figure-1. Study Schema

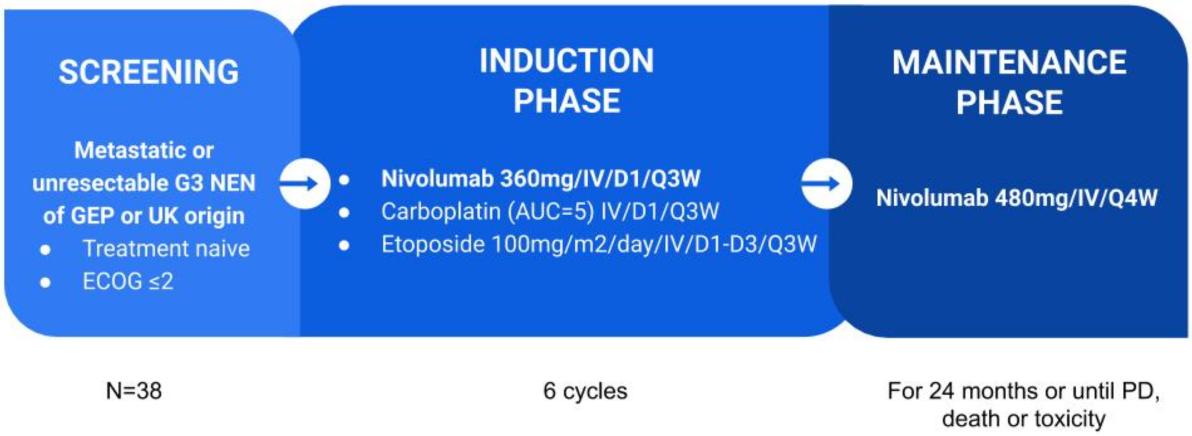

Study Population:
For study entry all the following criteria MUST be met:

Key Inclusion Criteria (See Protocol Section 4.1 for full list of criteria)

- Histologically confirmed G3 NENs originated in the gastroenteropancreatic tract (WHO 2015/2019 classification). Patients with a G3 NEN of unknown primary will also be eligible for this trial.
- Ki-67 >20% or mitotic rate > 20 per 10 HPF.
- Metastatic or locally advanced unresectable disease not amenable to treatment with curative intent.
- No prior systemic treatment for advanced disease nor as adjuvant therapy permitted.
- Availability of fresh or archive formalin-fixed, paraffin-embedded tumor tissue for biomarker assessment.
- Patients must have clinically and/or radiographically documented measurable disease.
- Adequate organ function as defined by the following criteria

    - Absolute neutrophil count (ANC) ≥1500 cells/mm3;
    - Platelets ≥100,000 cells/mm3;
    - Hemoglobin ≥9.0 g/dL;
    - AST and ALT ≤2.5 x upper limit of normal (ULN); in patients with liver metastases AST and ALT ≤5.0 x ULN;
    - Total bilirubin ≤1.5 x ULN;
    - Serum creatinine ≤1.5 x ULN or calculated creatinine clearance ≥60 mL/min.
- ECOG performance status of 0-2.

Key Exclusion Criteria (See Protocol Section 4.2 for full list of criteria)

- The following endocrine tumor types may not be included: paraganglioma, adrenal, thyroid parathyroid or pituitary endocrine tumors. Large or small cell lung neuroendocrine carcinoma of the lung will also be excluded.
- Prior therapy with any immune checkpoint inhibitor.
- Prior organ transplantation, including allogeneic stem-cell transplantation.
- Systemic chronic steroid therapy (≥ 10 mg/day prednisone or equivalent) or other immunosuppressive agents or use of any investigational drug within 28 days before the start of trial treatment.
- Known history of positive testing for Human Immunodeficiency Virus (HIV) infection, known history of positive tests for Hepatitis B virus surface antigen (HBVsAg) or Hepatitis C ribonucleic acid (HCV RNA) indicating acute or chronic infection or other significant acute or chronic infections requiring medication at study entry.

Study Assessments:

The primary objective is to determine one-year OS rate of nivolumab in combination with platinum-doublet chemotherapy in treatment-naïve patients with unresectable G3 NENs. This will be calculated from the date of treatment initiation with platinum-based chemotherapy and nivolumab until the date of death from any cause. Subjects will be assessed for response every 8 weeks (± 7 days) after the date of administration of the first dose for 12 months and every 12 weeks (± 7 days) thereafter until disease progression. Patients that stop therapy due to toxicity in the absence of disease progression shall continue to be assessed with CT scans with the same frequency until disease progression or initiation of a new line of therapy. Upon treatment discontinuation, subjects will be followed for survival every 3 months (via telephone contact allowed) until death. All patients included will be followed.

Statistical Considerations:

Sample Size

A sample size of 38 subjects will provide 80% power to test the null hypothesis (H_0) that one-year OS rate for advanced G3 NENs treated with nivolumab in combination with platinum-doublet chemotherapy is 0.5 against a two-sided alternative. This design yields a two-sided type I error rate of 5% when the true one-year OS rate is 0.72.

Study Calendar:

-	Study start (estimated)	3Q 2019
-	First patient First visit (estimated)	4Q 2019
-	End or recruitment (estimated)	2Q 2021
-	Last patient Last visit (estimated)	2Q 2022
-	End of study (estimation)	4Q 2022

Table of contents

1. Background and introduction **12**
1.1. Background Disease Information 12
1.2. Background Therapeutic Information 13
1.2.1. Nivolumab: mechanism of action 13
1.2.2. Preclinical experience with Nivolumab 13
1.2.3. Clinical experience with Nivolumab in SCLC 13
1.2.4. Immune checkpoint inhibitors in other high grade NENs 15
1.2.5. Chemotherapy in combination with immune checkpoint inhibitors 15
1.2.6. Potential for drug-drug interactions 17

2. Objectives and Endpoints of the trial **17**
2.1. Primary objective 17
2.2. Secondary Objectives 17

3. Study Design **18**
3.1. Overall Design 18
3.1.1. Data Monitoring Committee and Other External Committees 19
3.2. Number of participants 19
3.3. End of Study Definition 20
3.4. Study Calendar 20

4. Patient selection criteria **20**
4.1. Inclusion Criteria 20
4.2. Exclusion Criteria 21
4.3. Screening Failures 22
4.3.1. Retesting during Screening Period 22

5. Therapeutic Regimens, Expected Toxicity, Dose Modifications **22**
5.1. Treatments Administered 23
5.1.1. Dosing 24
5.1.1.1. Nivolumab 24
5.1.1.2 Chemotherapy dosing 24
5.1.1.3 Premedication 25
5.1.2. Patient Monitoring 25
5.1.3. Treatment of Nivolumab Infusion Reactions 26
5.2. Dose Adjustments 28
5.2.1. Dose modifications for Nivolumab 28
5.2.2. Dose modifications for Chemotherapy 32
5.2.2.1 Dose Reductions for Hematologic Adverse Events 32
5.2.2.2. Dose Reductions for Non-Hematologic Adverse Events 33
5.2.3. Dose Delay Criteria 34

5.2.3.1. Dose delay Criteria for Nivolumab 34
5.2.3.2. Dose delay Criteria for Chemotherapy 35
5.2.4. Criteria to Resume Dosing 35
5.2.4.1. Criteria to Resume Nivolumab Dosing 35
5.2.4.2. Criteria to Resume Treatment with Chemotherapy 36
5.3. Duration of Therapy 36
5.3.1. Treatment Beyond Disease Progression 36
5.4. Preparation/Handling/Storage/ Accountability 37
5.5. Concomitant therapy 38
5.5.1. Prohibited and/or Restricted Therapy 38
5.5.2. Permitted Therapy 38
5.5.3. Palliative Local Therapy 39
5.5.4. Imaging Restrictions and Precautions 39

6. Discontinuation Criteria 40
6.1. Discontinuation from Study Treatment 40
6.1.1. Nivolumab Dose Discontinuation 40
6.1.2. Chemotherapy Dose Discontinuation 41
6.2. Discontinuation from Study 42
6.2.1. Post Study Treatment Study Follow-up 43
6.3. Lost to Follow-up 43

7. Clinical evaluation, laboratory tests, follow-up 43
7.1. Before treatment start 44
7.1.1. Informed Consent 44
7.1.1.1. General Informed Consent 44
7.1.1.2. Consent and Collection of Specimens for Future Biomedical Research 44
7.1.2. Inclusion/Exclusion Criteria Assessment 44
7.1.3. Medical History 45
7.1.4. Prior and concomitant medications review 45
7.1.4.1. Prior Medications 45
7.1.4.2. Concomitant Medications 45
7.1.5. Assignment of Screening Number 45
7.1.6. Subject Identification Card 45
7.1.7. Submitting Tumor Sample 45
7.1.8. Tumor Imaging 46
7.1.8.1. Computed (CT) with IV contrast or Magnetic Resonance Imaging (MRI) of chest, abdomen and pelvis. 46
7.1.8.2. Brain CT with IV contrast or MRI scan 46
7.1.8.3. Whole body bone scan 46
7.1.9. Laboratory Assessments 46
7.1.10. 12-Lead Electrocardiogram (ECG) 47
7.2. During treatment 47

7.2.1. Adverse Event (AE) Monitoring 47
7.2.2. Physical Exam 47
7.2.3. Vital Signs 47
7.2.4. 12-Lead Electrocardiogram (ECG) 47
7.2.5. Eastern Cooperative Oncology Group (ECOG) Performance Status 47
7.2.6. Tumor Imaging and Assessment of Disease 48
7.2.7. Laboratory Assessments 48
7.2.8. Serum/Urine β-hCG 49
7.3. After the end of treatment (Follow-up) 49
7.4. Summary table 49
8. Evaluation criteria 50
8.1. Definitions 50
8.2. Evaluation of efficacy 51
8.2.1. Measurability of tumour lesions at baseline 51
8.2.1.1. Definitions 51
8.2.1.2. Methods of measurements 52
8.2.2. Tumor response evaluation 52
8.2.2.1. Response evaluation according RECIST 1.1 [xliv] 52
8.2.2.2. Response evaluation according irRECIST [xlvi] 54
8.2.2.3. Frequency of tumor re-evaluation 55
8.2.3. Reporting of tumor response 56
8.2.3.1. Response duration 56
8.2.3.2. Stable disease duration 56
8.2.3.3. Progression Free Survival 56
8.2.3.4. Overall Survival 56
9. Statistical considerations 56
9.1. Statistical design 56
9.1.1. Sample Size Determination 56
9.2. Populations for Analysis 57
9.3. Statistical Analysis 57
9.3.1. Efficacy Analysis 57
9.3.1.1. Methods for Primary Endpoint 58
9.3.1.2. Methods for Secondary Endpoints 58
9.3.2. Safety Analysis 58
10. Translational research 59
11. Patient registration procedure 59
12. Reporting adverse events 59
12.1. Definitions for adverse event reporting 59
12.2. Reporting procedure 60

13. Quality assurance **63**
13.1. Control of data consistency 63
13.2. On-site quality control (for multi-centre studies only when data are sent to a Data Centre or similar) 64
13.3. Audits (for multi-centre studies only when data are sent to Data Centre or similar) 64
13.4. Central review of pathology 65

14. Ethical considerations **65**
14.1. Patient protection 65
14.2. Subject identification 65
14.3. Informed consent 65

15. Publication policy **66**

APPENDICES **67**
Appendix A. Performance Status Criteria 67
Appendix B. References 68

Schema:

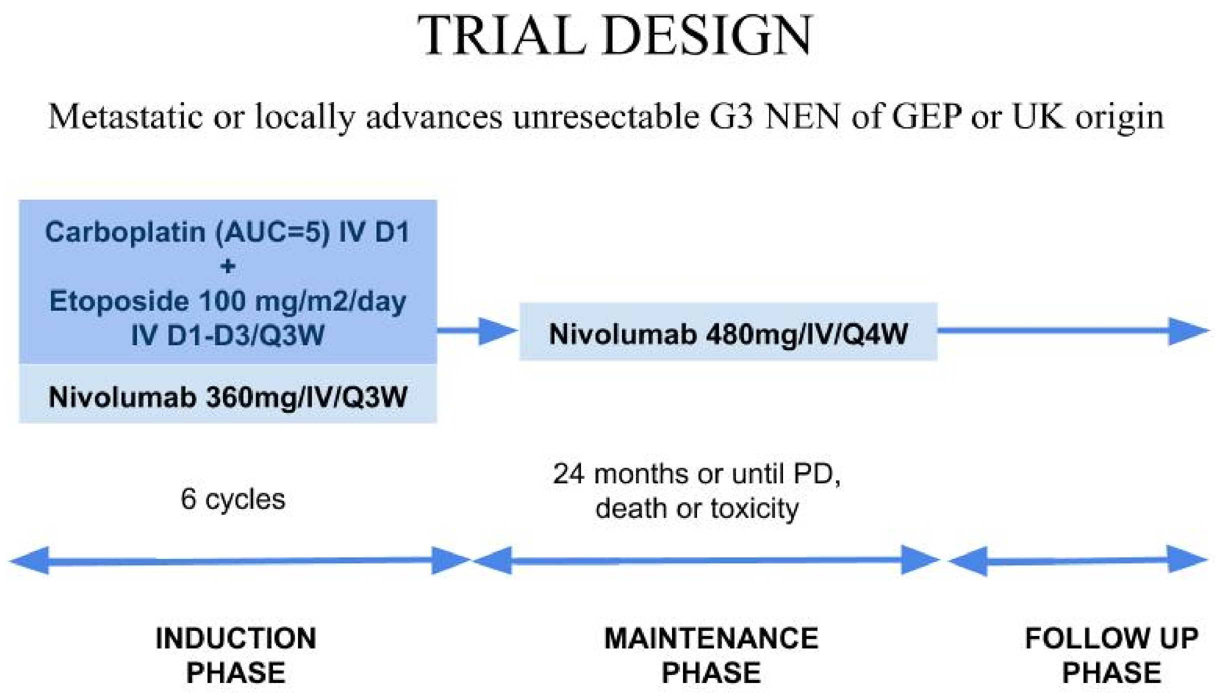

TRIAL DESIGN
Metastatic or locally advances unresectable G3 NEN of GEP or UK origin
Carboplatin (AUC=5) IV D1
+
Etoposide 100 mg/m2/day
IV D1-D3/Q3W
Nivolumab 360mg/IV/Q3W
Nivolumab 480mg/IV/Q4W
6 cycles
24 months or until PD,
death or toxicity
INDUCTION
PHASE
MAINTENANCE
PHASE
FOLLOW UP
PHASE

1. Background and introduction

1.1. Background Disease Information

NENs comprise a heterogeneous family of neoplasms with a wide and complex spectrum of clinical behaviour. They are uncommon tumours with an estimated incidence per year that ranges from 5 to 7 cases per 100,000 in the Caucasian population. The reported incidence has substantially increased over the last decades, al least partially due to improved diagnostic techniques and clinical awareness.[i],[ii]

G3 NENs are defined according to the World Health Organization (WHO) as those with high proliferative index (Ki-67 index greater than 20% or a mitotic rate greater than 20 per 10 high power fields (HPF)), which are generally, but not always, poorly differentiated tumors[iii].

The gastroenteropancreatic tract is the most common site for extra-pulmonary G3 NENs accounting for 35-55% of all NENs outside the lung. Approximately 50-60% of patients present with metastatic disease at the time of diagnosis and have a fateful prognosis. Median OS of patients with advanced disease is 5 months, ranging from less than a month for patients receiving best supportive care only, up to 11 months with best available therapy[iv],[v]. Historically, treatment strategies for extra-pulmonary G3 NENs have been often extrapolated from the treatment paradigm for small cell lung cancer (SCLC) given the histologic similarities between both entities. Therefore, standard front-line therapy involves the administration of a platinum agent (cisplatin or carboplatin) and etoposide, with response rates in the largest recent western series of ~ 30% and median survival of about one year [vi],[vii],[viii],[ix],[x]. The optimal duration of first line (1L) treatment is unclear. However, in the absence of progression, chemotherapy is usually stopped after 4 to 6 cycles due to cumulative toxicity and the lack of evidence demonstrating greater clinical benefit with extended treatment. Progression-free survival after this induction period remains poor, lasting 2- 3 months. Therefore, new therapeutic strategies are needed in this setting to improve response rates and prolong progression free survival without increasing toxicity in these patients. The addition of an immune checkpoint inhibitor to 1L chemotherapy, followed by maintenance immunotherapy could be a valuable option in this scenario.

More recently, a better understanding of the role of the immunological system in tumor control has opened multiple doors to implement different strategies to enhance immune response against cancer cells [xi]. It is well known that tumor cells elude immune response by several mechanisms. The programmed death-1 receptor (PD-1) and its ligand (PD-L1) are key therapeutic targets in the reactivation of the immune response against multiple cancers. PD-1 receptor is expressed on activated T cells and interacts with its ligand (PD-L1), which is expressed in tumor and immune cells, to down-regulate T-cell activation and promote *tumor immune escape*, a mechanism by which tumor cells avoid recognition and elimination by the immune system [xii]. The development of monoclonal antibodies against the PD-1/ PD-L1 pathway, that disrupt PD-1–mediated signalling and restore thereby antitumor immunity, has led to significant antitumor activity in a wide spectrum of neoplastic diseases, inducing increased progression-free-survival (PFS), overall survival (OS) and long lasting responses in different tumor types such as melanoma or lung cancer [xiii],[xiv],[xv],[xvi].

On the other hand, an increasing body of evidence has demonstrated that mutational burden is strongly associated with increased response to immunotherapy. In this regard, high grade NENs are suitable candidates for these treatment strategies as they are highly mutated tumours with high PD-L1

expression. For example, some authors have shown that virus-negative Merkel Cell Carcinomas (MCCs), a type of high grade NEN, harbour more tumor neoantigens than melanomas or non-small cell lung cancers (median of 173, 65, and 111 neoantigens/sample, respectively), two cancers for which immune checkpoint blockade can produce durable clinical responses [xvii].

In summary, the addition of a checkpoint inhibitor could be a valuable treatment option in these patients. The objective of this trial is to determine the one-year overall survival rate of nivolumab in addition to platinum-doublet chemotherapy in advanced G3 NENs without prior therapy.

1.2. Background Therapeutic Information

1.2.1. Nivolumab: mechanism of action

Nivolumab (BMS-936558) is a fully human, IgG4 mAb that binds PD-1 on activated immune cells and disrupts engagement of the receptor with its ligands PD-L1 and PD-L2, abrogating inhibitory signals and augmenting the host antitumor response. The PD-1 receptor-ligand interaction is a major pathway hijacked by tumors to suppress immune control. The normal function of PD-1, expressed on the cell surface of activated T cells under physiological conditions, is to down-modulate unwanted or excessive immune responses, including autoimmune reactions. PD-1 has been shown to negatively regulate antigen receptor signaling upon engagement of its ligands (PD-L1 and/or PD L2)[xviii]. These ligands are constitutively expressed or can be induced in a variety of cell types, including various tumors [xix],[xx],[xxi]. Binding of PD-1 to its ligands inhibits T cell activation triggered through the T-cell receptor. PD-L2 is thought to control immune T-cell activation in lymphoid organs, whereas PD-L1 serves to dampen unwarranted T-cell function in peripheral tissues. A variety of cancers have been shown to express abundant levels of this PD-L1. This suggests that the PD-1/PD-L1 pathway plays a critical role in tumor immune evasion and can be an attractive target for therapeutic intervention.

1.2.2. Preclinical experience with Nivolumab

In vitro, nivolumab (BMS-936558) binds to PD-1 with high affinity (EC50 0.39 - 2.62 nM), and inhibits the binding of PD-1 to its ligands PD-L1 and PD-L2 (IC50: 1 nM). Nivolumab binds specifically to PD-1 and not to related members of the CD28 family such as CD28, ICOS, CTLA-4, and BTLA. Blockade of the PD-1 pathway by nivolumab results in a reproducible enhancement of both proliferation and IFN-γ release in the mixed lymphocyte reaction (MLR). Using a CMV re-stimulation assay with human PBMC, the effect of nivolumab on antigen specific recall response indicates that nivolumab augmented IFN-γ secretion from CMV specific memory T cells in a dose-dependent manner versus isotype-matched control. In vivo blockade of PD-1 by a murine analog of nivolumab enhances the anti-tumor immune response and result in tumor rejection in several immunocompetent mouse tumor models (MC38, SA1/N, and PAN02)[xxii].

1.2.3. Clinical experience with Nivolumab in SCLC

In early clinical trials, nivolumab has demonstrated activity in several tumor types, including melanoma, renal cell cancer (RCC), and NSCLC [xxiii].

Nivolumab is now approved in the U.S. and Europe to treat different tumor types including: melanoma (both adjuvant and metastatic setting), metastatic NSCLC in patients with progression on or after platinum-based chemotherapy, advanced SCLC with progression after platinum-based chemotherapy, advanced renal cell carcinoma, metastatic urothelial cancer and advanced squamous cell carcinoma of head and neck [xxiv].

The approval in SCLC was based on the results of Checkmate-032 [xxv], This was a phase I/II multicentre, multi-arm, open-label trial that included a cohort of 216 patients with SCLC. Patients progressing after at least one platinum-containing therapy were allocated to three treatment arms: nivolumab plus ipilimumab [1 mg/kg + 1 mg/kg iv (n = 3), 1 mg/kg + 3 mg/kg iv (n = 61), and 3 mg/kg + 1 mg/kg iv (n = 54)] versus nivolumab monotherapy (3 mg/kg iv) (n = 98). Nivolumab plus ipilimumab was administered every 3 weeks for four cycles followed by nivolumab 3 mg/kg iv every other week. The primary endpoint was objective response per RECIST v1.1. Objective response was achieved in 14/61 (23%) receiving nivolumab 1 mg/kg plus ipilimumab 3 mg/kg; 10/54 (19%) receiving nivolumab 3 mg/kg plus ipilimumab 1 mg/kg, 1/3 (33%) receiving nivolumab 1 mg/kg plus ipilimumab 1 mg/kg, and 10/98 (10%) patients in the nivolumab monotherapy arm. The median duration of response was 17.9 months for nivolumab monotherapy, and 14.2 months with nivolumab 1 mg/kg plus ipilimumab 3 mg/kg. Patients with ongoing responses at 2 years were 45% for nivolumab monotherapy and 36% in the combination. Responses were observed regardless of platinum sensitivity, line of therapy, or PD-L1 status. Two-year OS rates were 14% for nivolumab monotherapy and 26% in the combination arm, with a median OS of 4.1 (95% CI 3.0–6.8) and 7.8 (95% CI 3.6–14.2), respectively. In a randomized, phase II cohort from CheckMate 032 to further evaluate nivolumab ± ipilimumab, the initial efficacy of 242 patients was consistent with that in the non-randomized cohort [xxvi]. Grade ≥ 3 toxicities occurred in 18/61 (30%) in the nivolumab 1 mg/kg plus ipilimumab 3 mg/kg group, 10/54 (19%) in the nivolumab 3 mg/kg plus ipilimumab 1 mg/kg, and 13/98 (13%) in the nivolumab monotherapy. Six (6%) patients in the nivolumab 3 mg/kg group, seven (11%) in the nivolumab 1 mg/kg plus ipilimumab 3 mg/kg group, and four (7%) in the nivolumab 3 mg/kg plus ipilimumab 1 mg/kg group discontinued treatment due to treatments related adverse events (TRAEs). Four patients who received nivolumab plus ipilimumab died from TRAEs (myasthenia gravis, pneumonitis, encephalitis and hepatitis), and one patient who received nivolumab monotherapy died from treatment-related pneumonitis.

Several trials are currently evaluating nivolumab ± ipilimumab. Initial results from the randomized phase III clinical trial Checkmate 331(NCT02481830) evaluating the role of nivolumab versus chemotherapy (topotecan or amrubicin) in patients with relapsed SCLC have been recently presented showing no statistically significant improvement in OS with nivolumab vs chemotherapy (HR, 0.86 [95% CI, 0.72–1.04]); however OS curves showed delayed separation after month 12. HR for OS with nivolumab vs chemotherapy in patients with platinum-resistant SCLC was 0.71 (95% CI, 0.54–0.94) [xxvii].

Checkmate 451 evaluated the role of nivolumab alone, nivolumab 1 mg/kg in combination with ipilimumab 3 mg/kg, or placebo as consolidation/maintenance therapy after completion of platinum-based first-line chemotherapy in patients with extended disease SCLC. The primary endpoint of OS has not been met [xxviii]. Results from a phase II trial of consolidation with nivolumab and ipilimumab in localized SCLC after chemo-radiotherapy are pending (STIMULI, NCT02046733)[xxix].

In general, nivolumab has been well tolerated to date, with a favorable safety profile relative to anticipated toxicities based on an immunostimulatory mechanism of action.

1.2.4. Immune checkpoint inhibitors in other high grade NENs

Further research of these therapeutic strategies in other types of high grade NENs, such as Merkel cell carcinomas (MCC), has also proven to be highly effective both in chemonaive and pretreated patients. In a multicenter phase 2 trial, patients with advanced MCC who had received no previous systemic therapy were assigned to receive pembrolizumab at a dose of 2 mg per kilogram every 3 weeks. The primary endpoint was the objective response rate (ORR). Twenty-six patients were treated with pembrolizumab, with an ORR of 56% (95% confidence interval [CI], 35 – 76%) and a rate of progression free survival at 6 months of 67% [xxx]. Avelumab, an anti-PD-L1 monoclonal antibody, also showed efficacy in MCC refractory to chemotherapy in a phase II trial that enrolled 88 patients with advanced disease. Responses were achieved in 32% of patients, 82% of which were maintained at 10 months of follow-up time. Grade 3 treatment-related events occurred in only 5% of patients with no grade 4 events described [xxxi]. These promising results represent a new therapeutic option for advanced MCC and open a new path for future clinical trials in G3 NENs. In SCLC, which shares many histopathological and biological features with extrapulmonary high grade NEN, Nivolumab has recently shown clinical activity in a phase I/II study of SCLC patients previously treated with platinum-based chemotherapy [xxxii]. An objective response rate of 18% was documented in 40 patients treated with nivolumab monotherapy. Pembrolizumab has also recently showed promising activity in a phase IB study of 20 patients who had received prior platinum-based combination chemotherapy [xxxiii]. Preliminary results reported objective responses in 35% of the treated patients. Nivolumab has also been tested in a cohort of 25 patients with advanced MCC within the CheckMate 358 trial (NCT02488759)[xxxiv]. Preliminary results have reported an overall response rate of 68% in 22 evaluable patients. Responses occurred in both treatment-naïve (71%) and -experienced (63%) patients, regardless of tumor MCPyV or PD-L1 expression. Expansion cohorts will evaluate combinations of nivolumab with ipilimumab or anti-LAG-3.

More recently, initial results from an open-label, phase 1b trial evaluating the efficacy and safety of JS001 on advanced NENs with high proliferative index (Ki67>10%) who had failed to standard therapy, have been presented. JS001 is a monoclonal humanized IgG4 PD-1 antibody with a hinge S228P mutation that completely blocks PD-1 interactions with PD-L1 and PD-L2. A total of 35 patients were enrolled, with a majority of NECs (n=28). An encouraging 18% overall response rate and 25% disease control rate was achieved in NECs, with a 50% response rate in patients with PD1-positive tumors, and the median OS had not been reached. JS001 was tolerable in patients with NENs with <3% of severe adverse events [xxxv]. Other checkpoint inhibitors have not shown significant activity as monotherapy in G3 heavily pretreated NEN (i.e. pembrolizumab, PDR001). Currently, ongoing trials are evaluating the role of these immunomodulating agents as maintenance treatment at earlier stages of disease, and in combination with different therapeutic strategies including chemotherapy.

1.2.5. Chemotherapy in combination with immune checkpoint inhibitors

There is increasing evidence suggesting that chemotherapy not only has antitumor activity due to cytotoxic effects, but also through immunological effects, including reducing T-regulatory cell activity and enhancing cross-presentation of tumour antigens [xxxvi],[xxxvii]. Chemotherapy has also been shown to induce PD-L1 expression on tumour cells. Therefore, combining immunotherapy and chemotherapy could synergistically improve the anticancer activity of anti-PD-1 and anti-PD-L1 monotherapy.

Results from PD-1 inhibitors in combination with platinum-based chemotherapy in first line phase Ib/II trials have shown promising results in NSCLC. A phase I multicohort study was conducted to explore the efficacy and safety of nivolumab in combination with standard chemotherapy in first-line NSCLC [xxxviii]. 56 patients were assigned by histology to receive nivolumab 10 mg/kg iv every 3 weeks plus gemcitabine and cisplatin (squamous) or pemetrexed plus cisplatin (non-squamous) or nivolumab 5 or 10 mg/kg plus carboplatin-paclitaxel (all histologies). Patients received the combination treatment for four cycles followed by nivolumab alone until progression or unacceptable toxicity. No dose-limiting toxicities (DLTs) occurred during the first 6 weeks of treatment. Consistent with previous reports for immune checkpoint inhibitors monotherapy, grade 3-4 treatment-related adverse events were reported in 45% of patients. ORR were 33%, 47%, 47% and 43% for nivolumab 10 mg/kg plus gemcitabine-cisplatin, nivolumab 10 mg/kg plus pemetrexed-cisplatin, nivolumab 10 mg/kg plus paclitaxel-carboplatin, and nivolumab 5 mg/kg plus paclitaxel-carboplatin respectively. Responses were achieved regardless of tumor programmed death ligand-1 expression. The safety profile of nivolumab was consistent with that expected for individual agents with no particular concerns.

Recently, the international, multicohort, phase 1/2 KEYNOTE-021 study assessed the safety and anti-tumour activity of pembrolizumab added to three different platinum-based chemotherapy doublets in patients with advanced NSCLC [xxxix]. Chemotherapy-naïve NSCLC pts were randomly assigned to pembrolizumab 2 or 10 mg/kg every 3 weeks plus carboplatin (AUC 6) + paclitaxel 200 mg/m2 (any histology) or carboplatin (AUC 6) + paclitaxel 200 mg/m2 + bevacizumab 15 mg/kg (non-squamous) or carboplatin (AUC 5) plus pemetrexed 500 mg/m2 (nonsquamous) for 4 cycles followed by maintenance treatment with pembrolizumab, pembrolizumab + bevacizumab or pembrolizumab plus pemetrexed respectively. Seventy-four pts were included in the study. All combinations showed promising anti-tumour activity irrespective of tumour PD-L1 expression, with manageable safety profiles. One DLT occurred in cycle 1 (grade 3 rash). Grade 3-4 treatment-related AEs occurred in 36%, 46% and 42% of pts in each cohort respectively. Most common AEs were AST elevation (n=3), anemia (n=2), neutropenia (n=2) and febrile neutropenia (n=2). Based on these results, 123 patients with advanced chemotherapy-naïve NSCLC were randomized (1:1) in an open-label phase II trial to four cycles of carboplatin (AUC 5) plus pemetrexed 500 mg/m2 iv every three weeks alone or in combination with pembrolizumab 200 mg iv, flat dose, every three weeks, followed by maintenance with pembrolizumab for 24 months and pemetrexed until progressive disease or unacceptable toxicity. Patients were stratified according to PD-L1 expression (<1% vs ≥ 1%). 55% of patients in the pembrolizumab plus chemotherapy group achieved an objective response compared with 29% in the chemotherapy alone group (p=0.0016). The incidence of grade 3 or worse treatment-related adverse events was similar between both arms occurring in 23 pts (39%) in the pembrolizumab plus chemotherapy arm and 16 (26%) in the chemotherapy alone arm. Most common grade 3 or worse treatment-related adverse events were anaemia (12% in the pembrolizumab plus chemotherapy group vs 15% in the chemotherapy alone group), decreased neutrophil count (5% vs 3%), thrombocytopenia (3% in both arms) and decreased lymphocyte count, neutropenia, and sepsis (3% vs 2% each). The incidence of adverse events based on a presumed immunological mechanism of action, was 22% in the pembrolizumab plus chemotherapy group and 11% in the chemotherapy group. The only events of grade 3 or worse were one infusion reaction (2% in the pembrolizumab plus chemotherapy group vs none in the chemotherapy alone group), grade 3 skin reaction (one [2%] in the pembrolizumab plus chemotherapy group vs one [2%] in the chemotherapy group, and grade 3 pneumonitis (one [2%] in the pembrolizumab plus chemotherapy group). Deaths attributed to study treatment occurred in one patient (1%) in the pembrolizumab plus chemotherapy group due to sepsis

and two patients (3%) in the chemotherapy alone group (pancytopenia and sepsis). This study showed that the combination of pembrolizumab and platinum chemotherapy could be an effective and tolerable first-line treatment option for patients with NSCLC [xl].

Furthermore, a recently published clinical trial assessing atezolizumab in combination with different platinum-doublets in patients with newly diagnosed metastatic NSCLC showed encouraging efficacy. Seventy-six NSCLC patients were enrolled and assigned to three different treatment arms: atezolizumab plus carboplatin and paclitaxel (Arm C), atezolizumab plus carboplatin and pemetrexed (Arm D) or atezolizumab plus carboplatin and nab-paclitaxel (Arm E). Common treatment-related grade 3-4 adverse events were neutropenia (36% Arm C, 36% Arm D, 42% Arm E) and anaemia (16% Arm C, 16% Arm D, 31% Arm E). Confirmed ORRs were 36% Arm C, 68% Arm D (one CR) and 46% Arm E (four CRs). Median PFS was 7.1 months, (95% CI: 4.2-8.3), 8.4 months (95% CI: 4.7-11) and 5.7 months (95% CI: 4.4-14.8), respectively. Median OS was 12.9 months (95% CI: 8.8-21.3), 18.9 months (95% CI: 9.9-27.4) and 17.0 months (95% CI: 12.7-not evaluable), respectively. Atezolizumab was well tolerated in combination with all chemotherapy regimens tested[xli],[xlii].

Currently several phase Ib/II trials evaluating the combination of immune checkpoint inhibitors plus chemotherapy are ongoing.

1.2.6. Potential for drug-drug interactions

Given nivolumab is a therapeutic monoclonal antibody, it is not anticipated to be directly eliminated through hepatic/renal metabolism to compete with the elimination of platinum-doublet chemotherapy agents, which are mainly eliminated through metabolism and renal excretion.

As nivolumab is not considered a cytokine modulator, it is unlikely to have an effect on drug metabolizing enzymes or transporters in terms of inhibition or induction such as cytochrome P450 (CYP) enzymes. Therefore the risk of drug-drug interactions between nivolumab and platinum-doublet chemotherapy agents is anticipated to be low.

2. Objectives and Endpoints of the trial

The objectives and endpoints of the trial are summarized in Table 2-1.

2.1. Primary objective

- To determine the efficacy of nivolumab in combination with platinum-chemotherapy in patients with high grade neuroendocrine neoplasms, evaluated as one-year overall survival (OS) rate calculated from the date of treatment initiation until the date of death from any cause.

2.2. Secondary Objectives

- To determine overall response rate (ORR) as per Response Evaluation Criteria in Solid Tumors version 1.1 (RECIST v1.1) and the immune-related RECIST (irRECIST).
- To determine progression free survival (PFS) calculated from the date of treatment initiation with first-line chemotherapy and nivolumab until the date of first documentation of progressive disease as per RECIST v1.1 or death.
- To determine the OS calculated from the date of treatment initiation with nivolumab and platinum-based chemotherapy until the date of death from any cause.

- To assess biochemical response in patients with baseline elevation of chromogranin A and/or enolase, and correlate it with clinical outcome.
- To assess the safety and toxicity profile of nivolumab in combination with platinum-doublets in this patient population according to the Common Terminology Criteria for Adverse Events v.5.0 (CTCAE).
- To explore potential predictive or prognostic blood or tissue biomarkers.

Objectives	Endpoints
**Primary**<br>● To determine the overall survival patients with advanced G3 NENs treated with nivolumab + platinum-based chemotherapy.	● 1 year-OS rate
**Secondary**<br>● To determine other efficacy outcomes of nivolumab + platinum-based chemotherapy in patients with advanced G3 NENs.	● ORR<br>● Duration of response<br>● PFS<br>● OS
● To evaluate the safety and tolerability of nivolumab + platinum-based chemotherapy in this patient population.	● Incidence of AEs, SAEs and selected AEs
● To evaluate biochemical response as predictive biomarker of efficacy of nivolumab + chemotherapy in this patient population..	● Chromogranin A and enolase values and their association with ORR, PFS and OS.
● To explore potential predictive and prognostic biomarkers.	● Mutational burden, gene expression signature, soluble factors and other molecular markers in peripheral blood and their association with clinical outcomes.

3. Study Design

3.1. Overall Design

This is a multi-center phase II non-randomized, open-label trial investigating platinum-doublet chemotherapy in combination with nivolumab in patients with advanced G3 NENs of GEP or UK origin followed by maintenance treatment with nivolumab for up to 2 years in patients who have

achieved complete response (CR), partial response (PR) or stable disease (SD) after 6 months of first line platinum-based induction chemotherapy with nivolumab.

All participants will be treated as described below:

- 6 cycles of chemotherapy (carboplatin (AUC= 5) iv on day 1 and etoposide 100 mg/m2/day iv on days 1-3, administered every 3 weeks) in combination with nivolumab 360 mg iv q3w (flat dose).
- In the absence of disease progression, nivolumab treatment will be continued until disease progression, unacceptable toxicity, death, withdrawal of consent, or a maximum treatment duration of 2 years, whichever occurs first. Participants will be permitted to continue on nivolumab beyond initial RECIST 1.1 defined progression, as long as they meet the criteria described in *section 5.3.1*

The study design schematic is presented in Figure 3.1-1

Figure 3.1-1

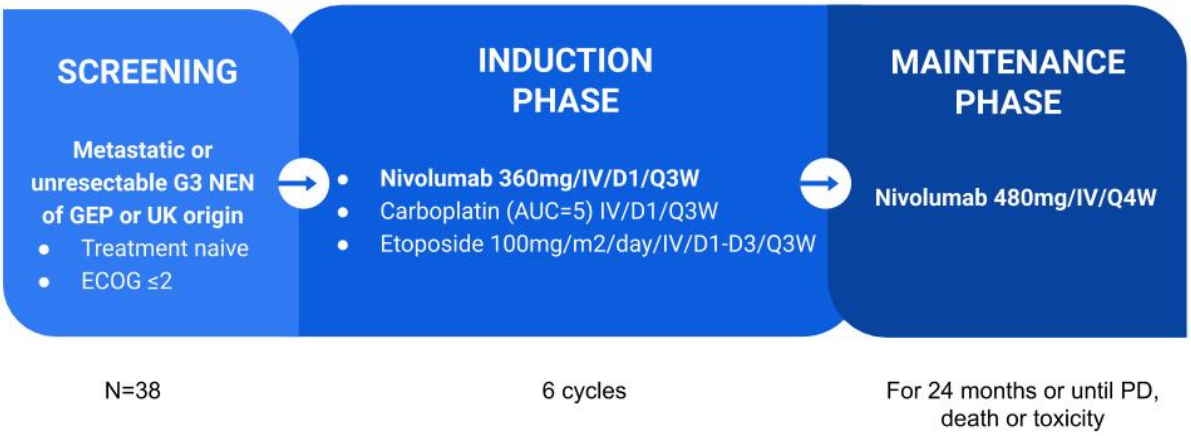

<u>3.1.1. Data Monitoring Committee and Other External Committees</u>

A Data Monitoring Committee (DMC) will not be required for this study, considering the following:

1. This study is an open-label study.
2. Data from the Checkmate-012 study has demonstrated a favourable and manageable safety profile that is consistent with prior experience with these agents.
3. Safety data will be closely monitored by BMS and the pharmacovigilance team of MFAR, with real time review and assessment of SAEs as they are received, and periodic review of all adverse events data for potential new safety signals.

3.2. Number of participants

The planned sample size is 38 patients with G3 NENs of gastroenteropancreatic or unknown primary origin.

Participating centres will include 10 academic hospitals selected from the GETNE Spanish network (Please see *Section 9*, statistical considerations, for specific details).

3.3. End of Study Definition

The duration of the study from start of enrollment to the last participant inclusion is expected to be 18 months. The study will end once additional survival follow-up of 12 months has concluded.

3.4. Study Calendar

- Study start (estimated)	3Q 2019
- First patient First visit (estimated)	4Q 2019
- End or recruitment (estimated)	2Q 2021
- Last patient Last visit (estimated)	2Q 2022
- End of study (estimation)	4Q 2022

4. Patient selection criteria

The following eligibility criteria are designed to select subjects for whom protocol treatment is considered appropriate. All relevant medical and non-medical conditions should be taken into consideration when deciding whether this protocol is suitable for a particular subject.

4.1. Inclusion Criteria

Patients should meet all of the following inclusion criteria:

1. Histologically confirmed G3 NENs originated in the gastroenteropancreatic tract (WHO 2010 classification). Patients with a G3 NEN of unknown primary will also be eligible for this trial.
2. Ki-67 >20% or mitotic rate > 20 per 10 HPF.
3. Metastatic or locally advanced unresectable disease not amenable to treatment with curative intent.
4. No prior systemic treatment for advanced disease nor as adjuvant therapy.
5. Availability of fresh or archive formalin-fixed, paraffin-embedded tumor tissue for biomarker assessment.
6. Patients must have clinically and/or radiographically documented measurable disease. At least one site of disease must be unidimensionally measurable as per RECIST 1.1.
7. Adequate organ function as defined by the following criteria (within 7 days prior to enrollment):
    a. absolute neutrophil count (ANC) ≥1500 cells/mm3
    b. platelets ≥100,000 cells/mm3
    c. hemoglobin ≥9.0 g/dL
    d. AST and ALT ≤2.5 x upper limit of normal (ULN); in patients with liver metastases AST and ALT ≤5.0 x ULN
    e. total bilirubin ≤1.5 x ULN
    f. serum creatinine ≤1.5 x ULN or calculated creatinine clearance ≥60 mL/min.
8. Male or female, age ≥18 years.
9. ECOG performance status of 0-2.
10. Life expectancy of ≥12 weeks.

11. Women of childbearing potential must have a negative serum or urine pregnancy test within 7 days prior to treatment initiation.
12. Highly effective contraception (i.e. methods with a failure rate of less than 1 % per year) for both fertile, sexually active male and female subjects. Highly effective contraception must be used 28 days prior to first trial treatment administration, for the duration of trial treatment, and at least for 60 days after stopping trial treatment.
13. Signed and dated informed consent document must be given according to ICH/GCP, and national/local regulations indicating that the patient (or legally acceptable representative) has been informed of all pertinent aspects of the trial prior to enrolment.

4.2. Exclusion Criteria

Subjects shall not meet any of the following exclusion criteria:

1. The following endocrine tumor types may not be included: paraganglioma, adrenal, thyroid parathyroid or pituitary endocrine tumors. Large or small cell lung neuroendocrine carcinoma of the lung will also be excluded.
2. Prior therapy with any immune checkpoint inhibitor.
3. Major surgery, except diagnostic biopsy, in <4 weeks or radiation therapy <2 weeks prior to starting study treatment. Prior palliative radiotherapy to metastatic lesion(s) is permitted, provided there is at least one measurable lesion that has not been irradiated.
4. Prior organ transplantation, including allogeneic stem-cell transplantation.
5. Prior history of non-infectious pneumonitis requiring steroids or current pneumonitis.
6. Systemic chronic steroid therapy (> 10 mg/day prednisone or equivalent) or other immunosuppressive agents or use of any investigational drug within 28 days before the start of trial treatment. Short-term administration of steroids for allergic reactions or management of immune-related adverse events is allowed. Topical, inhaled, nasal and ophthalmic steroids are also allowed.
7. Use of any live vaccines against infectious diseases within 4 weeks of initiation of study treatment.
8. Known history of positive testing for Human Immunodeficiency Virus (HIV) infection, known history of or positive tests for Hepatitis B virus surface antigen (HBVsAg) or Hepatitis C ribonucleic acid (HCV RNA) indicating acute or chronic infection or other significant acute or chronic infections requiring medication at study entry.
9. Active, known or suspected autoimmune disease or a documented history of autoimmune disease, including ulcerative colitis and Crohn’s disease. (Patients with vitiligo, type I diabetes mellitus, residual hypothyroidism due to autoimmune condition only requiring hormone replacement, psoriasis not requiring systemic treatment, or conditions not expected to recur in the absence of an external trigger are permitted to enroll).
10. Active seizure disorder or evidence of brain metastases, spinal cord compression, or carcinomatous meningitis.
11. A serious uncontrolled medical disorder or active infection that would impair their ability to receive study treatment will not be allowed to enter the study. Any of the following within the 12 months prior to study drug administration: myocardial infarction, uncontrolled angina, coronary/peripheral artery bypass graft, NYHA class ≥ III congestive heart failure, cerebrovascular accident or transient ischemic attack and 6 months for deep vein thrombosis or pulmonary embolism.

12. Known hypersensitivity reactions to monoclonal antibodies (≥ grade 3 according to NCI Common Terminology Criteria for Adverse Events (CTCAE) v 5.0 [xliii] or any past medical history of anaphylaxis or uncontrolled asthma (i.e., 3 or more asthma characteristics partially controlled).
13. Any other prior malignancy within 5 years of study entry, with the exception of adequately treated in-situ carcinoma of the cervix, breast or uteri, or non-melanomatous skin cancer.
14. Any psychological, familial, sociological or geographical condition potentially hampering compliance with the study protocol and follow-up schedule; those conditions should be discussed with the patient before registration in the trial.
15. Dementia or significantly altered mental status that would prohibit the understanding or rendering of informed consent and compliance with the requirements of this protocol.
16. Female patients who are pregnant or lactating, or men and women of reproductive potential not willing or not able to employ an effective method of birth control/contraception to prevent pregnancy during treatment and for 6 months after discontinuing study treatment The definition of effective contraception should be in agreement with local regulation and based on the judgment of the principal investigator or a designated associate.
17. Other severe acute or chronic medical or psychiatric condition, or laboratory abnormality that may increase the risk associated with study participation or study drug administration, or may interfere with the interpretation of study results, and in the judgment of the investigator would make the patient inappropriate for study entry.

4.3. Screening Failures

Screening failures are defined as participants who consent to participate in the clinical study but are not subsequently randomized. A minimal set of screen failure information is required to ensure transparent reporting of screen failure participants, to meet the Consolidated Standards of Reporting Trials (CONSORT) publishing requirements, and to respond to queries from regulatory authorities. Minimal information includes date of consent, demography, screen failure details, eligibility criteria, and any serious AEs.

4.3.1. Retesting during Screening Period

This study permits the re-enrollment of a participant that has discontinued the study as a pre-treatment failure (ie, participant has not been treated). If re-enrolled, the participant must be re-consented. Retesting of laboratory parameters and/or other assessments within any single screening will be permitted (in addition to any parameters that require a confirmatory value).

5. Therapeutic Regimens, Expected Toxicity, Dose Modifications

Study treatment is defined as any investigational treatment(s), marketed product(s), placebo or medical device intended to be administered to a study participant according to the study treatment allocation.

Study treatment includes both Investigational [Medicinal] Product (IP/IMP) and Non-investigational [Medicinal] Product (Non-IP/Non-IMP) and consist of the following:

- Nivolumab
- Carboplatin
- Etoposide

Other medications used as support or escape medication for preventative, diagnostic, or therapeutic reasons, as components of the standard of care for a given diagnosis, may be considered as non-investigational products.

Study Treatments for Study CA209- 73D

Product description and dosage Form	Potency	IP/Non-IP	Blinded or Open Label	Primary Packaging (Volume)/Label Type and Secondary Packaging (Qty)/Label Type	Appearance	Storage Conditions (per label)
BMS-936558-01 Nivolumab Solution for Injection[a]	100 mg/vial (10 mg/mL)	IP	Open Label	10 mL per vial and 5 or 10 vials per carton	Clear to opalescent colorless to pale yellow liquid. May contain particles	2 to 8 C. Protect from light and freezing
Carboplatin Solution for injection[b]	450 mg/vial (10 mg/mL)	IP	Open Label	Various packaging configuration	Clear, colorless or slightly yellow solution.	Product should be stored as per market product conditions.
Etoposide	100 mg/ vial (20mg/ mL)	IP	Open Label	Various packaging configuration	Powder, white to white with a yellow cast.	Product should be stored as per market product conditions.

NOTES: [a] May be labeled as either “BMS-936558-01” or “Nivolumab”

5.1. Treatments Administered

The treatments that will be used in this trial are outlined below (Table-5.1). They will be administered in the order presented.

All trial treatments may be administered on an outpatient basis.

Table-5.1. Trial treatments

Drug	Drug	Frequency	Administration	Treatment period	Use
Nivolumab	360 mg	Q3W	IV infusion	Day 1 of each cycle	Experimental
Carboplatin	AUC5	Q3W	IV infusion	Day 1 of each cycle	Standard
Etoposide	100 $mg/m^2/day$	Q3W	IV infusion	Days 1-3 of each cycle	Standard

Nivolumab will be administered first as a 30 minute IV infusion, followed by the carboplatin infusion and then the etoposide infusion. Platinum-doublet will start at least 30 minutes after completion of the nivolumab infusion. At the investigator's discretion, nivolumab may be administered over a longer infusion time (60 minutes) if the participant developed a prior infusion reaction.

Nivolumab treatment will continue until disease progression, discontinuation due to unacceptable toxicity, or withdrawal of consent. Participants will be treated up to 24 months in the absence of disease progression or unacceptable toxicity.

All participants will be monitored continuously for adverse events (AEs) while on study treatment. Treatment modifications (eg, dose delay, reduction, retreatment, or discontinuation) will be based on specific laboratory and adverse event criteria, as described in *sections 5.2-5.3.*

5.1.1. Dosing

5.1.1.1. Nivolumab

Participants will receive nivolumab, followed by chemotherapy on day 1 of every 3 weeks cycle for 6 cycles (*induction phase*). At the time of completion of 6 cycles of chemotherapy and nivolumab, participants who have not experienced disease progression will continue to receive nivolumab at a dose of 480 mg as 30 minute infusion every 4 weeks for up to 2 years (*maintenance phase*).

Drug	Dose	Frequency	Administration	Treatment period	Use
**Induction phase**					
Nivolumab	360 mg	Q3W	IV infusion	Day 1 of each cycle	Experimental
**Maintenance phase**					
Nivolumab	480 mg	Q4W	IV infusion	Day 1 of each cycle	Experimental

Treatment will continue until progression, unacceptable toxicity, withdrawal of consent, whichever occurs first. Treatment with nivolumab will be given for up to 24 months including the induction phase (a total of 34 cycles) in the absence of disease progression or unacceptable toxicity.

The assessment for discontinuation of nivolumab should be made separately from chemotherapy. If criteria for discontinuation for nivolumab are met, platinum-based doublet chemotherapy may continue until 6 cycles have been completed. If a participant meets criteria for discontinuation and investigator is unable to determine whether the event is related to all or one study drug, the participant should discontinue all study drugs and be taken off the study.

5.1.1.2 Chemotherapy dosing

6 cycles of carboplatin in combination with etoposide will be administered every 3 weeks (Q3W).

All chemotherapy agents' preparation, premedication, administration, monitoring, and management of complications are to follow local prescription guidelines and regulations. The dose of chemotherapy may be capped per local standards.

- ❑ Carboplatin:

Carboplatin (AUC=5) will be administered as a 15-60 minute IV infusion per site's standard practice Q3W on day 1 of each treatment cycle. Carboplatin will be administered to participants at least 30 minutes following the end of the nivolumab infusion.
The dose of carboplatin will be calculated using the *Calvert formula* as follows:

Carboplatin dose (mg)= AUC target x ([CrCl (ml/min) + 25]

Creatinine clearance (CrCl) calculation is based on the *Cockcroft-Gault formula* and should include the most recent serum creatinine and the most recent weight.

NOTE: If calculation of the CrCl by the Cockcroft-Gault formula yields a result of > 125 mL/min, then a CrCl should be calculated by an alternative formula per institutional standards or capped at 125 mL/min.
The dose of carboplatin may be capped per local standards.

- ❑ Etoposide:

Etoposide 100 mg/m^2/day will be administered as a 30-60 minute IV infusion per site's standard practice Q3W on days 1, 2 and 3 of each treatment cycle. Etoposide will be administered following the end of carboplatin infusion.

5.1.1.3 Premedication

- ❑ Nivolumab

Patients should not receive pre-medication to prevent infusion reaction before the first infusion of nivolumab. If a patient experiences an infusion reaction, he/she may receive premedication prior to subsequent dosing days (*Table 5.1.3).*

- ❑ Carboplatin and Etoposide:

Antiemetic premedication will be administered according to local standards. Recommended antiemetic treatments are:

- ❑ Dexamethasone (dosing according to local standards; an equivalent dose of another corticosteroid may be administered).
- ❑ A 5-HT3 receptor antagonist (type per investigator discretion and local standards-of-care).

Additional use of antiemetic premedications may be employed at the discretion of the Investigator. Please refer to the product label or local standards of care for carboplatin and etoposide supportive measures.

5.1.2. Patient Monitoring

Vital signs including temperature, pulse, respiratory rate, weight and blood pressure prior to the administration of each dose of trial treatment will be measured as specified in *section 7.4.*

5.1.3. Treatment of Nivolumab Infusion Reactions

Since nivolumab contains only human immunoglobulin protein sequences, it is unlikely to be immunogenic and induce infusion or hypersensitivity reactions. However, if such a reaction were to occur, it might manifest with fever, chills, rigors, headache, rash, pruritus, arthralgias, hypo- or hypertension, bronchospasm, or other symptoms. All Grade 3 or 4 infusion reactions should be reported within 24 hours to the BMS Medical Monitor and reported as a SAE if criteria are met. Infusion reactions should be graded according to NCI CTCAE (Version 5.0) guidelines.

Treatment recommendations are provided below and may be modified based on local treatment standards and guidelines, as appropriate:

For Grade 1 symptoms: (mild reaction; infusion interruption not indicated; intervention not indicated)

- Remain at bedside and monitor the patient until recovery from symptoms. The following prophylactic premedications are recommended for future infusions: diphenhydramine 50 mg (or equivalent) and/or acetaminophen/paracetamol 325 to 1000 mg at least 30 minutes before additional nivolumab administration.

For Grade 2 symptoms: (moderate reaction requires therapy or infusion interruption but responds promptly to symptomatic treatment [eg, antihistamines, non-steroidal anti-inflammatory drugs, narcotics, corticosteroids, bronchodilators, IV fluids]; prophylactic medications indicated for < 24 hours)

- Stop the nivolumab infusion, begin an IV infusion of normal saline, and treat the participant with diphenhydramine 50 mg IV (or equivalent) and/or acetaminophen/paracetamol 325 to 1000 mg; remain at bedside and monitor participant until resolution of symptoms. Corticosteroid and/or bronchodilator therapy may also be administered as appropriate. If the infusion is interrupted, then restart the infusion at 50% of the original infusion rate when symptoms resolve; if no further complications ensue after 30 minutes, the rate may be increased to 100% of the original infusion rate. Monitor participant closely. If symptoms recur, then no further nivolumab will be administered at that visit. Administer diphenhydramine 50 mg IV, and remain at bedside and monitor the participant until resolution of symptoms. The amount of study drug infused must be recorded on the electronic case report form (eCRF).
- For future infusions, the following prophylactic premedications are recommended: diphenhydramine 50 mg (or equivalent) and/or acetaminophen/paracetamol 325 to 1000 mg should be administered at least 30 minutes before nivolumab infusion. If necessary, corticosteroids (up to 25 mg of hydrocortisone or equivalent) may be used.

For Grade 3 or 4 symptoms: (severe reaction, Grade 3: prolonged [ie, not rapidly responsive to symptomatic medication and/or brief interruption of infusion]; recurrence of symptoms following initial improvement; hospitalization indicated for other clinical sequelae [eg, renal impairment, pulmonary infiltrates]. Grade 4: Life threatening; pressor or ventilator support indicated)

- Immediately discontinue infusion of nivolumab. Begin an IV infusion of normal saline and treat the participant as follows: Recommend bronchodilators, epinephrine 0.2 to 1mg of a 1:1000 solution for subcutaneous administration or 0.1 to 0.25 mg of a 1:10.000 solution injected slowly for IV administration, and/or diphenhydramine 50 mg IV with methylprednisolone 100 mg IV (or equivalent), as needed. Participant should be monitored

until the investigator is comfortable that the symptoms will not recur. Nivolumab will be permanently discontinued. Investigators should follow their institutional guidelines for the treatment of anaphylaxis. Remain at bedside and monitor participant until recovery of the symptoms.

In case of late-occurring hypersensitivity symptoms (eg, appearance of a localized or generalized pruritus within 1 week after treatment), symptomatic treatment may be given (eg, oral antihistamine or corticosteroids).

Table 5.1.3 Infusion reaction guidelines

Grade	Treatment	Premedication at subsequent dosing
Grade 1	Increase monitoring of vital signs until the subject is deemed medically stable in the opinion of the investigator.	Subject may be premedicated at least 30 minutes prior to nivolumab infusion with:<br>● Diphenhydramine 50 mg po (or equivalent dose of antihistamine).<br>● Acetaminophen 325-1000 mg po.
Grade 2	**Stop Infusion and monitor symptoms.**<br>Treat the participant with:<br>● IV infusion of normal saline<br>● Diphenhydramine 50 mg IV<br>● Acetaminophen/ paracetamol 325-1000mg.<br>● Increase monitoring of vital signs as medically indicated until the subject is deemed medically stable in the opinion of the investigator.<br>● If symptoms resolve within one hour of stopping drug infusion, the infusion may be restarted at 50% of the original infusion rate (e.g., from 100 mL/hr to 50 mL/hr). Otherwise dosing will be held until symptoms resolve and the subject should be premedicated for the next scheduled dose.	Subject should be premedicated at least 30 minutes prior to nivolumab infusion with:<br>● Diphenhydramine 50 mg po (or equivalent dose of antihistamine).<br>● Acetaminophen 325-1000 mg po.<br>● If necessary corticosteroids may be used (up to 25 mg of hydrocortisone or equivalent).
Grade 3-4	**Stop Infusion.**<br>Treat the participant with:<br>● IV infusion of normal saline.<br>● Bronchodilators if required.<br>● Epinephrine 0.2 to 1 mg of a 1:1000 solution sc.<br>● Diphenhydramine 50 mg IV.<br>● Methylprednisolone 100 mg IV (or equivalent).<br>● Remain at bedside and monitor participant until recovery of the symptoms and stable in the opinion of the investigator.<br>● Hospitalization may be indicated.<br><br>**Nivolumab permanently discontinued**	**No subsequent dosing**
Appropriate resuscitation equipment should be available in the room and a physician readily available during the period of drug administration.		

5.2. Dose Adjustments

- The Investigator may attribute each toxicity event to carboplatin, etoposide or nivolumab alone and use a dose reduction according to *Tables 5.2, 5.1.3.* For individual subjects requiring a dose modification, treatment for each new cycle may be delayed if the scheduled off-drug periods are not adequate to allow for recovery to Grade ≤1 or the baseline status of the subject.
- Nivolumab dose reductions are not permitted. Nivolumab treatment may be interrupted or discontinued due to toxicity for up to 6 weeks (longer interruptions shall be discussed and approved by the medical monitor on an individual basis). If toxicity has not recovered to at least a grade 1 the patient shall be permanently discontinued from the study. Longer dose interruptions.
- If a dose reduction for toxicity occurs with any agent, the dose may not be re-escalated. Subjects can have a maximum of 2 dose modifications to each of the components of study therapy throughout the course of the study for toxicities. If a subject experiences several toxicities and there are conflicting recommendations, dose reduction will be done according to the most severe toxicity. Subjects who require a 3rd dose modification to any particular component will have that agent discontinued.
- Reduction of one chemotherapy agent and not the other agent is appropriate if, in the opinion of the investigator, the toxicity is clearly related to one of the treatments. If, in the opinion of the investigator, the toxicity is related to the combination of both chemotherapy agents, both drugs should be reduced according to recommended dose modifications. If the toxicity is related to the combination of three agents, chemotherapy should be reduced, interrupted or discontinued and nivolumab should be interrupted or discontinued according to the recommended dose modifications.

Table 5.2. Dose modifications for trial medications

Drug	Dose level 0	Dose level -1	Dose level -2	Dose level -3
Carboplatin	AUC 5	AUC 4	AUC 3	Discontinue
Etoposide	100 mg/m2	80 mg/m2	60 mg/m2	Discontinue
Nivolumab	360 mg/m2	Dose reductions not permitted		

5.2.1. Dose modifications for Nivolumab

There will be no dose reductions for nivolumab. Adverse events (AEs), both non-serious and serious, associated with nivolumab exposure may represent an immunologic etiology. These adverse events may occur shortly after the first dose or several months after the last dose of treatment. AEs can differ in severity and duration from AEs caused by other therapeutic classes. Early recognition and management of AEs associated with immune-oncology agents may mitigate severe toxicity.

Nivolumab must be withheld for drug-related toxicities and severe or life-threatening AEs as per *Table 5.2.1* below.

Table 5.2.1 Nivolumab guidelines for drug-related adverse events.

Toxicity	Grade of event	Management	Follow-up
**Diarrhea/Colitis**	1	Continue immunotherapy per protocol.<br>Symptomatic treatment	-Close monitoring for worsening symptoms.<br>-Educate patient to report worsening immediately.<br>If worsens:<br>-Treat as Grade 2 or 3/4.
	2	Hold treatment until toxicity resolves to grade 0-1.<br>Symptomatic treatment.	If improves to grade 1:<br>-resume therapy per protocol<br>If persists> 5-7 days or recur:<br>-0.5-1.0 mg/kg/day methylprednisolone or equivalent.<br>-When symptoms improve to grade 1, taper steroids over at least 1 month, consider prophylactic antibiotic for opportunistic infections, and resume immunotherapy per protocol.<br>If worsens or persists>3-5 days with oral steroids:<br>-Treat as grade 3-4.
	3-4	-Discontinue treatment.<br>-1.0 to 2.0 mg/kg/day methylprednisolone IV or equivalent.<br>- Add prophylactic antibiotics for opportunistic infections.<br>-Consider lower endoscopy	If improves:<br>-Continue steroids until grade 1, then taper over at least 1 month.<br>If persists >3-5 days or recurs after improvement:<br>-Add infliximab 5mg/kg (if no contraindication).<br>Note: infliximab should not be used in cases of perforation or sepsis.
**AST, ALT, Bilirubin**	1	Continue treatment per protocol	Continue LFT monitoring per protocol.<br>If worsens:<br>-Treat as Grade 2 or 3-4.
	2	-Hold treatment until toxicity resolves to grade 0-1<br>-Increase frequency of monitoring to every 3 days.	If returns to baseline:<br>-Resume routine monitoring, resume therapy per protocol.<br>If elevations persist >5-7 days or worsen:<br>-0.5-1 mg/kg/day methylprednisolone or oral equivalent and when LFT returns to grade 1 or baseline, taper steroids over at least 1 month, consider prophylactic antibiotics for opportunistic infections and resumen therapy per protocol.

Toxicity	Grade of event	Management	Follow-up
**AST, ALT, Bilirubin**	3-4	-Discontinue nivolumab [1]<br>-Increase frequency of monitoring to every 1-2 days.<br>-1.0 to 2.0 mg/kg/day methylprednisolone IV or IV equivalent [2]	If returns to grade 2:<br>-Taper steroids over at least 1 month.<br>If does not improve in >3-5 days, worsens or rebounds:<br>-Add mycophenolate mofetil 1 g BID.<br>-If no response within an additional 3-5 days, consider other immunosuppressants per local guidelines.
**Skin Rash**	1-2	-Symptomatic therapy (eg. antihistamines, topical steroids).<br>-Continue nivolumab per protocol.	If persists >1-2 weeks or recurs:<br>-Consider skin biopsy.<br>-Delay nivolumab per protocol.<br>-Consider 0.5-1.0 mg/kg/day methylprednisolone IV or oral equivalent. Once improving, taper steroids over at least 1 month, consider prophylactic antibiotics for opportunistic infections, and resume nivolumab per protocol.<br>If worsens:<br>-Treat as Grade 3-4.
	3-4	-Hold or discontinue nivolumab per protocol.<br>-Consider skin biopsy.<br>-Dermatology consult.<br>-1.0 to 2.0 mg/kg/day methylprednisolone IV or IV equivalent.	If it improves to Grade 1:<br>-Taper steroids over at least 1 month and add prophylactic antibiotics for opportunistic infections.<br>- Resume nivolumab per protocol.
**Pneumonitis**	1	-Consider delay of nivolumab.<br>-Monitor for symptoms every 2-3 days.<br>-Consider Pulmonary and ID consults.	-Re-image at least every 3 weeks.<br>If worsens:<br>-Treat as Grade 2 or 3-4
	2	-Delay of nivolumab per protocol.<br>-Monitor for symptoms daily,consider hospitalization.<br>- Pulmonary and ID consults.<br>-1.0 mg/kg/day methylprednisolone IV or oral equivalent.<br>- Consider bronchoscopy, lung biopsy.	-Re-image every 1-3 days.<br>If improves:<br>-When symptoms return to near baseline, taper steroids over at least 1 month and then resume nivolumab per protocol and consider prophylactic antibiotics.<br>If not improving after 2 weeks or worsening:<br>-Treat as Grade 3-4.

Toxicity	Grade of event	Management	Follow-up
**Pneumonitis**	3-4	-Discontinue nivolumab per protocol.<br>-Hospitalize.<br>-Pulmonary and ID consults.<br>-2-4 mg/kg/day methylprednisolone IV or equivalent.<br>-Add prophylactic antibiotics for opportunistic infections.<br>- Consider bronchoscopy, lung biopsy.	<u>If improves to baseline:</u><br>-Taper steroids over at least 6 weeks.<br><u>If not improving after 48 hours or worsening</u>:<br>-Add additional immunosuppression.
**Renal failure or nephritis**	1	-Continue nivolumab per protocol.<br>-Monitor creatinine weekly	<u>If returns to baseline:</u><br>-Resume routine creatinine monitoring per protocol.<br><u>If worsens:</u><br>-Treat as Grade 2 or 3-4.
	2-3	-Hold nivolumab per protocol.<br>-Monitor creatinine every 2-3 days.<br>0.5-1 mg/kg/day methylprednisolone IV or equivalent.<br>-Consider renal biopsy with nephrology consult.	<u>If returns to Grade 1</u>:<br>-Taper steroids over at least 1 month, consider prophylactic antibiotics for opportunistic infections, and resume nivolumab and routine creatinine monitoring per protocol.<br><br><u>If elevations persist >7 days or worsen:</u><br>-Treat as Grade 4.
	4	-Discontinue nivolumab per protocol.<br>-Monitor creatinine daily.<br>-1.0-2.0mg/kg/day methylprednisolone IV or IV equivalent.<br>-Consider renal biopsy.<br>- Consult nephrologist.	<u>If returns to Grade 1</u>:<br>-Taper steroids over at least 1 month, consider prophylactic antibiotics for opportunistic infections.
	**Management**		**Follow-up**
**Asymptomatic TSH elevation**	-Continue nivolumab per protocol.<br>-If TSH<0.5 X LLN, or TSH>2 x ULN, or consistently out of range in 2 subsequent measurements :include fT4 at subsequent cycles as clinically indicated; consider endocrinology consult.		

Toxicity	Management	Follow-up
**Symptomatic endocrinopathy**	-Evaluate endocrine function.<br>-Consider pituitary scan.<br>Symptomatic with abnormal lab/pituitary scan:<br>-Delay nivolumab per protocol.<br>-1-2 mg/kg/day methylprednisolone or PO equivalent.<br>-Initiate appropriate hormone therapy.<br>No abnormal lab/pituitary MRI scan but symptoms persist:<br>-Repeat labs in 1-3 weeks/MRI in 1 month.	If improves (with or without hormone replacement):<br>-Taper steroids over at least 1 month, consider prophylactic antibiotics for opportunistic infections.<br>-Resume nivolumab per protocol.<br>- Patients with adrenal insufficiency may need to continue steroids with mineralocorticoid component.
**Suspicion of adrenal crisis (e.g. severe dehydration, hypotension, shock out of proportion to current illness)**	-Delay or Discontinue nivolumab therapy per protocol.<br>-Rule out sepsis.<br>-Stress dose of IV steroids with mineralocorticoid activity.<br>-IV fluids.<br>-Consult endocrinologist.<br>-If adrenal crisis ruled out, then trat as above for symptomatic endocrinopathy.	
[1] Nivolumab may be delayed rather than discontinued if AST/ALT ≤ 8 X ULN or Total Bilirubin ≤ 5 x ULN.<br>[2] The recommended starting dose for grade 4 hepatitis is 2 mg/kg/day methylprednisolone IV.<br>[3] Patients with intolerable or persistent Grade 2 drug-related AE may hold study medication at physician discretion. Permanently discontinue study drug for persistent Grade 2 adverse reactions for which treatment with study drug has been held, that do not recover to Grade 0-1 within 12 weeks of the last dose.		

5.2.2. Dose modifications for Chemotherapy

Dose reductions of chemotherapy may be required, and will be performed according to *Table 5.2.2.* Chemotherapy dose reductions are permanent; once the dose of any chemotherapy agent is reduced, it may not be re-escalated in subsequent cycles. The dose reductions for each agent in the platinum-based doublet chemotherapy regimen are not linked and may be adjusted independently as summarized below.

Table 5.2.2 Dose modifications for Chemotherapeutic Agents

Drug	Dose level 0	Dose level -1	Dose level -2	Dose level -3
Carboplatin	AUC 5	AUC 4	AUC 3	Discontinue
Etoposide	100 mg/m2	80 mg/m2	60 mg/m2	Discontinue

Any participants with two prior dose reductions for one agent who experiences a toxicity that would cause a third dose reduction must be discontinued from that agent.

5.2.2.1 Dose Reductions for Hematologic Adverse Events

Dose modifications for hematologic toxicities (according to CTCAE version 5.0) are summarized in *Table-5.2.2.1*.

Dose adjustments are based on nadir blood counts (assessed as per local standards) since the preceding drug administration. Dose level adjustments for platinum-based doublet chemotherapy are relative to that of the preceding administration. Generally, both chemotherapy agents in the platinum-based doublet chemotherapy regimen should be dose reduced together for hematologic toxicity. After the first cycle, growth factors may be used to assist hematologic recovery. Use local standards of care in the use of these supportive measures. Additionally, prophylactic antibiotics may be used according to local standards of care. Please report any antibiotic or growth factor use on the eCRF.

Table 5.2.2.1 Dose modifications for carboplatin/etoposide hematologic adverse events

Toxicity	Grade	Action	Treatment Restart	Treatment discontinuation
Neutropenia	3	Hold treatment until Neutrophil count resolves to >1,500/mm3	No dose reduction required if neutropenia does not induce treatment delay. If neutropenia induces treatment delay consider G-CSF support. If already con G-CSF support reduce by 1 DL (at the discretion of the investigator)	Toxicity does not resolve within 6 weeks of last infusion or if > 2 Dose Level reductions are exceeded
	4	Hold treatment until neutrophil count resolves to >1,500/mm^3	Reduce by 1 DL *consider G-CSF	
Febrile Neutropenia	3-4	Hold treatment until toxicity resolves to Grade 0-1	Reduce by 1 DL	Toxicity does not resolve within 6 weeks of last infusion or if > 2 Dose Level reductions are exceeded
Thrombocytopenia	3-4	Platelet count resolves to >100,000/mm3	Reduce by 1 DL	Toxicity does not resolve within 6 weeks of last infusion or if > 2 Dose Level reductions exceeded

5.2.2.2. Dose Reductions for Non-Hematologic Adverse Events

Dose adjustments for chemotherapy for non-hematologic toxicities during treatment are described in Section 5.2.2. All dose reductions should be made based on the worst grade toxicity during the prior cycle. Participants experiencing any toxicity during the previous cycle should have their chemotherapy delayed up to 3 weeks (6 weeks since the administration of the last chemotherapy infusion) until retreatment criteria are met and then reduced for all subsequent cycles by 1 dose level

or discontinued as appropriate. Dose levels for the two drugs in the platinum-doublet chemotherapy regimen are not linked and may be reduced independently, as summarized in *Table-5.2.2.2.*

Table 5.2.2.2. Dose modifications for non-hematologic adverse events

Toxicity	Grade	Action	Treatment Restart
Creatinine increased	2	Hold treatment until toxicity resolves to Grade 0-1	Reduce cisplatin by 1 DL
	3-4[1]	Discontinue carboplatin if creatinine clearance <20 ml/min	Discontinue treatment
Ototoxicity or sensory neuropathy	2	Hold treatment until toxicity resolves to Grade 0-1	Reduce cisplatin by 1 DL
	≥ 3	Discontinue chemotherapy	Discontinue chemotherapy
Allergic reaction [a]	≥ 3	Discontinue chemotherapy	Discontinue chemotherapy
All other non-hematological toxicities	3-4	Hold treatment until toxicity resolves to Grade 0-1.	Reduce by 1 DL

[a] Only the drug(s) causing the hypersensitivity reaction or acute infusion reaction (≥ Grade 3) require discontinuation. All other drugs may be continued.

5.2.3. Dose Delay Criteria

5.2.3.1. Dose delay Criteria for Nivolumab

Tumor assessments for all participants should continue as per protocol even if dosing is delayed.
Nivolumab administration should be delayed for the following:

- Any Grade ≥ 2 non-skin, drug-related adverse event, except for fatigue and laboratory abnormalities.
- Any Grade ≥ 3 skin drug-related AE.
- Any Grade ≥ 3 drug-related laboratory abnormality with the following exceptions for lymphopenia, AST, ALT, or total bilirubin or asymptomatic amylase or lipase:
  - Grade 3 lymphopenia does not require a dose delay

If a participant has a baseline AST, ALT, or total bilirubin that is within normal limits, delay dosing for drug-related Grade 2 toxicity.

If a participant has baseline AST, ALT, or total bilirubin within the Grade 1 toxicity range, delay dosing for drug-related Grade ≥ 3 toxicity.

- Any Grade ≥ 3 drug-related amylase or lipase abnormality that is not associated with symptoms or clinical manifestations of pancreatitis does not require dose delay.
- Any AE, laboratory abnormality or intercurrent illness, which, in the judgment of the investigator, warrants delaying the dose of study medication.

Participants who require delay should be re-evaluated weekly or more frequently if clinically indicated. Participants should resume dosing when re-treatment criteria are met.

5.2.3.2. Dose delay Criteria for Chemotherapy

Chemotherapy drugs should be delayed for any of the following on the Day 1 of each cycle:

- Absolute neutrophil count (ANC) < 1500/L
- Platelets < 100,000/mm3
- Any Grade ≥ 2 non-skin, non-hematologic, drug-related adverse event (excluding Grade 2 alopecia, Grade 2 fatigue, and Grade 2 laboratory abnormalities)
- Any Grade ≥ 3 skin, drug-related adverse event
- Any Grade ≥ 3 drug-related laboratory abnormality, with the following exceptions for lymphopenia, AST, ALT, or total bilirubin:
    - Grade 3 lymphopenia does not require dose delay.
    - If a participant has a baseline AST, ALT or total bilirubin that is within normal limits, delay dosing for drug-related Grade ≥ 2 toxicity.
    - o If a participant has baseline AST, ALT, or total bilirubin within the Grade 1 toxicity range, delay dosing for drug-related Grade ≥ 3 toxicity.
- Any adverse event, laboratory abnormality, or intercurrent illness, which, in the judgment of the investigator, warrants delaying the dose of study medication.

Investigators should consult local labeling for the chemotherapy drugs being administered to any given participant for additional guidance on dose delays.

If any non-hematologic adverse event meeting the dose delay criteria above is felt to be related to only one particular agent in the platinum-based doublet chemotherapy regimen, then that agent alone may be omitted for that cycle while the other agent is given. In order to maintain synchronized dosing of the regimen, the omitted agent should be resumed with the next scheduled cycle once the AE has improved and retreatment criteria are met. Please refer to *Section 5.2.2* to determine if dose reduction of the resumed agent is required.

If both drugs in the platinum-based doublet chemotherapy regimen are delayed, then the participant should be re-evaluated weekly or more frequently if clinically indicated until retreatment criteria are met (as per *Section 5.2.4*).

<u>5.2.4. Criteria to Resume Dosing</u>

5.2.4.1. Criteria to Resume Nivolumab Dosing

Participants may resume treatment with nivolumab when the drug-related AE(s) resolve(s) to Grade ≤ 1 or baseline, with the following exceptions:

- Participants may resume treatment in the presence of Grade 2 fatigue.
- Participants who have not experienced a Grade 3 drug-related skin AE may resume treatment in the presence of Grade 2 skin toxicity.
- Participants with baseline Grade 1 AST/ALT or total bilirubin who require dose delays for reasons other than a 2-grade shift in AST/ALT or total bilirubin may resume treatment in the presence of Grade 2 AST/ALT OR total bilirubin.
- Participants with combined Grade 2 AST/ALT and total bilirubin values meeting discontinuation parameters (*Section 6.1.1*) should have treatment permanently discontinued.
- Drug-related pulmonary toxicity, diarrhea, or colitis must have resolved to baseline before treatment is resumed. Participants with persistent Grade 1 pneumonitis after completion of a

steroid taper over at least 1 month may be eligible for retreatment if discussed with and approved by the BMS Medical Monitor.

- Participants who received systemic corticosteroids for management of any drug-related toxicity must be off corticosteroids or have tapered down to an equivalent dose of prednisone ≤ 10 mg/day.
- Drug-related endocrinopathies adequately controlled with only physiologic hormone replacement may resume treatment.
- Dose delay of nivolumab which results in treatment interruption of > 6 weeks requires treatment discontinuation, with exceptions as noted in *Section 6.1.1*

5.2.4.2. Criteria to Resume Treatment with Chemotherapy

- Participants may resume treatment with chemotherapy when the ANC returns to 1500/µl, the platelet count returns to 100,000/mm3, and all other drug-related toxicities have returned to baseline or Grade 1 (or Grade 2 for alopecia and fatigue).
- If a participant fails to meet criteria for re-treatment, then re-treatment should be delayed, and the participant should be re-evaluated weekly or more frequently as clinically indicated. Any participant who fails to recover from toxicity attributable to chemotherapy to baseline or Grade 1 (except Grade 2 alopecia and fatigue) within 6 weeks from the last dose given should discontinue the drug(s) that caused the delay.
- When resuming chemotherapy treatment, please follow the dose reduction recommendations in *Section 5.2.2.*

5.3. Duration of Therapy

- During the "induction phase" six cycles of chemotherapy in combination with nivolumab will be given unless disease progression (see definition Section 7) or unacceptable toxicity are encountered.
- If no progression or unacceptable toxicity are observed after the induction phase, the patient will continue to receive nivolumab 480 mg every 4 weeks. The maximum treatment duration is 24 months or 34 cycles including the induction phase.
- <u>End of treatment</u>
  Patients will continue study treatment until disease progression per RECIST 1.1 or, unacceptable toxicity, withdrawal of consent, physician's decision, subject/guardian decision, lost to follow-up, death, study treatment completion (24 months) or the study is terminated by the Sponsor.

<u>5.3.1. Treatment Beyond Disease Progression</u>

Accumulating evidence indicates a minority of participants treated with immunotherapy may derive clinical benefit despite initial evidence of PD.

Participants will be permitted to continue on nivolumab for treatment beyond initial RECIST 1.1 defined PD as long as they meet the following criteria:

- Investigator-assessed clinical benefit and no rapid disease progression.
- Participant is tolerating study treatment.
- Stable performance status.

- Treatment beyond progression will not delay an imminent intervention to prevent serious complications of disease progression (eg, CNS metastases).
- Participant provides written informed consent prior to receiving additional nivolumab treatment, using an ICF describing any reasonably foreseeable risks or discomforts, or other alternative treatment options.

The decision to continue treatment beyond initial investigator-assessed progression should be discussed with the BMS Medical Monitor and documented in the study records. A follow-up scan should be performed within six weeks +/- 7 days of original PD to determine whether there has been a decrease in the tumor size, or continued progression of disease. Subsequent scans should be performed per protocol defined schedule +/- 7 days until further progression is determined.
If the investigator feels that the participant continues to achieve clinical benefit by continuing treatment, the participant should remain on the trial and continue to receive monitoring according to the Time and Events Schedule in Section *7.2*.

For the participants who continue study therapy beyond progression, further progression is defined as an additional 10% increase in tumor burden from time of initial PD. This includes an increase in the sum of diameters of all target lesions and/or the diameters of new measurable lesions compared to the time of initial PD. Nivolumab treatment should be discontinued permanently upon documentation of further progression.

New lesions are considered measurable at the time of initial progression if the longest diameter is at least 10 mm (except for pathological lymph nodes which must have a short axis of at least 15 mm).
Any new lesion considered non-measurable at the time of initial progression may become measurable and therefore included in the tumor burden if the longest diameter increases to at least 10 mm (except for pathological lymph nodes which must have a short axis of at least 15 mm). In situations where the relative increase in total tumor burden by 10% is solely due to inclusion of new lesions which become measurable, these new lesions must demonstrate an absolute increase of at least 5 mm.

5.4. Preparation/Handling/Storage/ Accountability

The investigational product should be stored in a secure area according to local regulations. It is the responsibility of the investigator to ensure that investigational product is only dispensed to study participants. The investigational product must be dispensed only from official study sites by authorized personnel according to local regulations.

The product storage manager should ensure that the study treatment is stored in accordance with the environmental conditions (temperature, light, and humidity) as determined by BMS. If concerns regarding the quality or appearance of the study treatment arise, the study treatment should not be dispensed and contact BMS immediately.

Study treatment not supplied by BMS will be stored in accordance with the package insert.

Investigational product documentation (whether supplied by BMS or not) must be maintained that includes all processes required to ensure drug is accurately administered. This includes documentation of drug storage, administration and, as applicable, storage temperatures, reconstitution, and use of required processes (eg, required diluents, administration sets).

5.5. Concomitant therapy

5.5.1. Prohibited and/or Restricted Therapy

Subjects are prohibited from receiving the following therapies during screening to the end of treatment of this trial:

- Antineoplastic systemic chemotherapy or biological therapy not specified in the protocol
- Immunotherapy not specified in this protocol.
- Investigational agents other than nivolumab.
- Radiation therapy; palliative radiation therapy to a symptomatic lesion (e.g. bony metastasis), or to the brain may be permitted.
- Live vaccines within 30 days prior to the first dose of trial treatment and while participating in the trial. Live vaccines include, but are not limited to, the following: measles, mumps, rubella, chicken pox, yellow fever, rabies, BCG, and typhoid (oral) vaccine. The killed virus vaccines used for seasonal influenza vaccines for injection are allowed; however live attenuated intranasal influenza vaccines (e.g. Flu - Mist®) are not allowed.
- The use of systemic steroid therapy and other immunosuppressive drugs is not allowed except for the treatment of infusion reaction, irAEs, and for prophylaxis against imaging contrast dye allergy or replacement-dose steroids in the setting of adrenal insufficiency (providing this is <10 mg/day prednisone or equivalent), or transient exacerbations of other underlying diseases such as COPD requiring treatment for ≤ 3 weeks. If systemic corticosteroids are required for the control of infusion reactions or irAEs, it must be tapered and be at non immunosuppressive doses (< 10 mg/day of prednisone or equivalent) before the next administration of study treatment. If the dose of prednisone or equivalent cannot be reduced to less than 10 mg/day before the administration of next dose of study treatment then nivolumab must be discontinued.

Caution should be used regarding the use of herbal medications as there may be yet unknown interactions with nivolumab. Discontinuation of the use of herbal medications prior to study enrollment is encouraged. Except for the permitted procedures specified as palliative local therapies (Section 5.5.3), all other radiation therapy or surgery to any tumor lesion is not permitted during study treatment.

There are no prohibited therapies during the post-treatment follow-up phase.

5.5.2. Permitted Therapy

All treatments that the investigator considers necessary for a subject’s welfare may be administered at the discretion of the investigator in keeping with the standards of medical care.

Participants are permitted the use of topical, ocular, intra-articular, intranasal, and inhalational corticosteroids (with minimal systemic absorption). Adrenal replacement steroid doses >10 mg daily prednisone are permitted. A brief (less than 3 weeks) course of corticosteroids for prophylaxis (eg, contrast dye allergy) or for treatment of non-autoimmune conditions (eg, delayed-type hypersensitivity reaction caused by a contact allergen) is permitted.

All concomitant medication will be recorded on the case report form (CRF) including all prescription, over-the-counter (OTC), herbal supplements, and IV medications and fluids.

If changes occur during the trial period, documentation of drug dosage, frequency, route, and date may also be included on the CRF.

All concomitant medications received during the trial through 30 days after the last dose of trial treatment should be recorded.

5.5.3. Palliative Local Therapy

Palliative local therapy, including palliative radiation therapy and palliative surgical resection, to symptomatic non-target bone lesions, skin lesions, or CNS lesions is permitted prior to discontinuation of study treatment for participants who do not have evidence of overall clinical or radiographic progression per RECIST 1.1.

Palliative local therapy to lesions causing gastrointestinal bleeding may also be permitted prior to discontinuation of study treatment in participants who do not have evidence of overall clinical or radiographic progression per RECIST 1.1, provided that the lesions undergoing palliative local therapy are not the only sites of measurable disease and the case is discussed with and approved by the BMS Medical Monitor.

Participants requiring palliative local therapy should be evaluated for objective evidence of disease progression prior to the initiation of such therapy, particularly if the most recent tumor assessment was more than 4 weeks prior to the start of local therapy. If progression per RECIST 1.1 is identified on any tumor assessments prior to the initiation of palliative local therapy, then participants must either discontinue study drug treatment or they must meet criteria to continue treatment beyond progression (*Section 5.3.1*) in order to resume immunotherapy after palliative local therapy.

The potential for overlapping toxicities with radiotherapy and nivolumab currently is not known; however, anecdotal data suggests that it is tolerable. As concurrent radiotherapy and nivolumab have not been formally evaluated, in cases where palliative radiotherapy is required for a tumor lesion, then nivolumab should be withheld for at least 1 week before, during, and 1 week after radiation. Participants should be closely monitored for any potential toxicity during and after receiving radiotherapy, and AEs should resolve to Grade ≤ 1 prior to resuming nivolumab.

5.5.4. Imaging Restrictions and Precautions

It is the local imaging facility’s responsibility to determine, based on participant attributes (eg, allergy history, diabetic history and renal status), the appropriate imaging modality and contrast regimen for each participant. Imaging contraindications and contrast risks should be considered in this assessment. Participants with renal insufficiency should be assessed as to whether or not they should receive contrast and if so, what type and dose of contrast is appropriate. Should a participant have a contraindication for CT IV contrast, a non-contrast CT of the chest and a contrast enhanced MRI of the abdomen and pelvis may be obtained.

Specific to MRI, participants with severe renal insufficiency (ie, estimated glomerular filtration rate (eGFR) < 30 mL/min/1.73 m2) are at increased risk of nephrogenic systemic fibrosis. MRI contrast should not be given to this participant population. In addition, participants are excluded from MRI if they have tattoos, metallic implants, pacemakers, etc. The ultimate decision to perform MRI in an individual participant in this study rests with the site radiologist, the investigator and the standard set by the local Ethics Committee.

6. Discontinuation Criteria

6.1. Discontinuation from Study Treatment

For all participants, global deterioration of health status requiring discontinuation of treatment without objective evidence of disease progression at that time should be reported as ‘symptomatic deterioration’ in the source data and in the case report form.

Tumor assessments for participants, who discontinue study treatment without radiographic progression, should continue as per protocol until radiographic progression is determined.

Chemotherapy dose reduction is allowed on study. Any participant with two prior dose reductions to one agent who experiences a toxicity that would cause a third dose reduction must be discontinued from that agent. A participant who is discontinued from the chemotherapy treatment will remain on the study and receive nivolumab.

6.1.1. Nivolumab Dose Discontinuation

Treatment with nivolumab should be permanently discontinued for any of the following:

- Any Grade ≥ 2 drug-related uveitis or eye pain or blurred vision that does not respond to topical therapy and does not improve to Grade 1 severity within the re-treatment period OR requires systemic treatment.
- Any Grade ≥ 2 drug-related pneumonitis or interstitial lung disease that does not resolve after a dose delay and systemic steroids (also see *Table 5.2.1).*
- Any Grade 3 drug-related bronchospasm, hypersensitivity reaction, or infusion reaction, regardless of duration;
- Any Grade 3 non-skin, drug-related adverse event lasting > 7 days, with the following exceptions for uveitis, pneumonitis, bronchospasm, diarrhea, colitis, neurologic toxicity, hypersensitivity reactions, infusion reactions, endocrinopathies, and laboratory abnormalities:
    - Grade 3 drug-related uveitis, pneumonitis, bronchospasm, diarrhea, colitis, neurologic toxicity, hypersensitivity reaction, or infusion reaction of any duration requires discontinuation.
    - Grade 3 drug-related endocrinopathies adequately controlled with only physiologic hormone replacement do not require discontinuation.
    - Grade 3 drug-related laboratory abnormalities do not require treatment discontinuation except:
        - Grade 3 drug-related thrombocytopenia > 7 days or associated with bleeding requires discontinuation.
        - Any drug-related liver function test (LFT) abnormality that meets the following criteria require discontinuation: Grade ≥ 3 drug-related AST, ALT or Total Bilirubin requires discontinuation.*

    * In most cases of Grade 3 AST or ALT elevation, study drugs(s) will be permanently discontinued. If the investigator determines a possible favorable benefit/risk ratio that warrants continuation of study drugs(s), a discussion between the investigator and the BMS medical monitor or designee must occur.

    Concurrent AST or ALT > 3x ULN and total bilirubin > 2x ULN
- Any Grade 4 drug-related adverse event or laboratory abnormality, except for the following events, which do not require discontinuation:
    - Grade 4 neutropenia ≤ 7 days.

    - Grade 4 lymphopenia or leukopenia.
    - Isolated Grade 4 amylase or lipase abnormalities that are not associated with symptoms or clinical manifestations of pancreatitis and decrease to < Grade 4 within 1 week of onset.
    - Isolated Grade 4 electrolyte imbalances/abnormalities that are not associated with clinical sequelae and are corrected with supplementation/appropriate management within 72 hours of their onset.
- Grade 4 drug-related endocrinopathy adverse events such as adrenal insufficiency, ACTH deficiency, hyper- or hypothyroidism, or glucose intolerance, which resolve or are adequately controlled with physiologic hormone replacement (corticosteroids, thyroid hormones) or glucose controlling agents, respectively, may not require discontinuation after discussion with and approval from the BMS Medical Monitor.
- Dosing delays lasting > 6 weeks from the previous dose that occur for non-drug-related reasons may be allowed if approved by the BMS medical monitor. Prior to re-initiating treatment in a participant with a dosing delay lasting > 6 weeks, the BMS medical monitor must be consulted. Tumor assessments should continue as per protocol even if dosing is delayed. Periodic study visits to assess safety and laboratory studies should also continue every 6 weeks or more frequently if clinically indicated during such dosing delays.
- Any adverse event, laboratory abnormality, or intercurrent illness which, in the judgment of the Investigator, presents a substantial clinical risk to the participant with continued nivolumab dosing.

The assessment for discontinuation of nivolumab should be made separately from the assessment made for discontinuation of chemotherapy.

If a participant meets criteria for discontinuation and investigator is unable to determine whether the event is related to nivolumab or chemotherapy, the participant should discontinue all treatment.

The assessment for discontinuation of nivolumab should be made separately from the assessment made for discontinuation of chemotherapy doublet. If criteria for discontinuation for nivolumab are met before the nivolumab plus platinum-based doublet chemotherapy cycles have been completed, platinum-based doublet chemotherapy may continue until 6 cycles have been completed.

6.1.2. Chemotherapy Dose Discontinuation

Except where specified below, chemotherapy drugs in the platinum-based doublet chemotherapy regimen should be discontinued for any of the following:

- Any Grade ≥ 3 peripheral neuropathy
- Any drug-related liver function test (LFT) abnormality that meets the following criteria requires discontinuation:
    - AST or ALT > 5-10 x ULN for > 2 weeks
    - AST or ALT > 10 x ULN
    - Total bilirubin > 5 x ULN
    - Concurrent AST or ALT > 3x ULN and total bilirubin > 2 x ULN
- Any drug-related adverse event which recurs after two prior dose reductions for the same drug-related adverse event requires discontinuation of the drug(s) which was/were previously dose reduced.

- Any Grade ≥ 3 drug-related hypersensitivity reaction or infusion reaction requires discontinuation of the drug(s) felt to be causing the reaction. The drug not felt to be related to the hypersensitivity reaction or infusion reaction may be continued.
- Any Grade 4 drug-related adverse event which the investigator deems is inappropriate to be managed by dose reduction(s) requires discontinuation of the drug(s) felt to be causing the event. The drug not felt to be related to the event may be continued.
- Any event that leads to delay in dosing of any study drug(s) for > 6 weeks from the previous dose requires discontinuation of that drug(s) with the following exception:
    - Dosing delays lasting > 6 weeks from the previous dose that occur for non-drug-related reasons may be allowed if approved by the BMS medical monitor. Prior to re-initiating treatment in a participant with a dosing delay lasting > 6 weeks, the BMS medical monitor must be consulted. Periodic study visits to assess safety and laboratory studies should also continue every 6 weeks or more frequently if clinically indicated during such dosing delays.
- Any adverse event, laboratory abnormality, or intercurrent illness which, in the judgment of the Investigator, presents a substantial clinical risk to the participant with continued platinum-based doublet chemotherapy dosing. Investigators should consult local labeling for the chemotherapy drugs being administered to any given participant for additional guidance on dose discontinuation.

6.2. Discontinuation from Study

Participants who request to discontinue study treatment will remain in the study and must continue to be followed for protocol specified follow-up procedures. The only exception to this is when a participant specifically withdraws consent for any further contact with him/her or persons previously authorized by participant to provide this information.

- Participants should notify the investigator of the decision to withdraw consent from future follow-up in writing, whenever possible.
- The withdrawal of consent should be explained in detail in the medical records by the investigator, as to whether the withdrawal is from further treatment with study treatment only or also from study procedures and/or post treatment study follow-up, and entered on the appropriate CRF page.
- In the event that vital status (whether the participant is alive or dead) is being measured, publicly available information should be used to determine vital status only as appropriately directed in accordance with local law.
- If the participant withdraws consent for disclosure of future information, the sponsor may retain and continue to use any data collected before such a withdrawal of consent.

In the case of pregnancy, the investigator must immediately notify the BMS Medical Monitor/designee of this event. In the event a normal healthy female participant becomes pregnant during a clinical trial, the study treatment must be discontinued immediately. In most cases, the study treatment will be permanently discontinued in an appropriate manner (eg, dose tapering if necessary for participant safety). Please call the BMS Medical Monitor within 24 hours of awareness of the pregnancy. If the investigator determines a possible favorable benefit/risk ratio that warrants

continuation of study treatment, a discussion between the investigator and the BMS Medical Monitor/designee must occur.

All participants who discontinue study treatment should comply with protocol specified follow-up procedures as outlined in *Section 7*. The only exception to this requirement is when a participant withdraws consent for all study procedures including post-treatment study follow-up or loses the ability to consent freely (ie, is imprisoned or involuntarily incarcerated for the treatment of either a psychiatric or physical illness).

If study treatment is discontinued prior to the participant's completion of the study, the reason for the discontinuation must be documented in the participant's medical records and entered on the appropriate case report form (CRF) page.

6.2.1. Post Study Treatment Study Follow-up

In this study, OS is a key endpoint of the study. Post study follow-up is of critical importance and is essential to preserving participant safety and the integrity of the study. Participants who discontinue study treatment must continue to be followed for collection of outcome and/or survival follow-up data as required and in line with *Section 7* until death or the conclusion of the study.

6.3. Lost to Follow-up

All reasonable efforts must be made to locate participants to determine and report their ongoing status. This includes follow-up with persons authorized by the participant.

Lost to follow-up is defined by the inability to reach the participant after a minimum of three documented phone calls, faxes, or emails as well as lack of response by participant to one registered mail letter. All attempts should be documented in the participant's medical records.

If it is determined that the participant has died, the site will use permissible local methods to obtain date and cause of death.

If investigator's use of third party representative to assist in the follow-up portion of the study has been included in the participant's informed consent, then the investigator may use a Sponsor retained third party representative to assist site staff with obtaining participant's contact information or other public vital status data necessary to complete the follow-up portion of the study.

The site staff and representative will consult publicly available sources, such as public health registries and databases, in order to obtain updated contact information.

If after all attempts, the participant remains lost to follow-up, then the last known alive date as determined by the investigator should be reported and documented in the participant's medical records.

7. Clinical evaluation, laboratory tests, follow-up

Study procedures and timing are summarized in the Schedule of Activities.

- All immediate safety concerns must be discussed with the Sponsor immediately upon occurrence or awareness to determine if the participant should continue or discontinue treatment.
- Adherence to the study design requirements, including those specified in the Schedule of Activities, is essential and required for study conduct.
- All screening evaluations must be completed and reviewed to confirm that potential participants meet all eligibility criteria before randomization. The investigator will maintain a screening log to record details of all participants screened and to confirm eligibility or record reasons for screening failure, as applicable.

7.1. Before treatment start

7.1.1. Informed Consent

The investigator or qualified designee must obtain documented consent from each potential subject or each subject's legally acceptable representative prior to participating in a clinical trial or Future Biomedical Research.

7.1.1.1. General Informed Consent

- Consent must be documented by the subject's dated signature or by the subject's legally acceptable representative dated signature on a consent form along with the dated signature of the person conducting the consent discussion.
- A copy of the signed and dated consent form should be given to the subject before participation in the trial. The initial informed consent form, any subsequent revised written informed consent form and any written information provided to the subject must receive the IRB/ERC's approval/favorable opinion in advance of use.
- The subject or his/her legally acceptable representative should be informed in a timely manner if new information becomes available that may be relevant to the subject's willingness to continue participation in the trial. The communication of this information will be provided and documented via a revised consent form or addendum to the original consent form that captures the subject's dated signature or by the subject's legally acceptable representative dated signature.
- The informed consent will adhere to IRB/ERC requirements, applicable laws and regulations.

7.1.1.2. Consent and Collection of Specimens for Future Biomedical Research

- The investigator or qualified designee will explain the Future Biomedical Research consent to the subject, answer all of his/her questions, and obtain written informed consent before performing any procedure related to the Future Biomedical Research sub-trial.
- A copy of the informed consent will be given to the subject.

7.1.2. Inclusion/Exclusion Criteria Assessment

All inclusion and exclusion criteria will be reviewed by the investigator or qualified designee to ensure that the subject qualifies for the trial. More details on criteria for patients eligibility section 4.1 and 4.2.

7.1.3. Medical History

- A medical history will be obtained by the investigator or qualified designee. Medical history will include all active conditions, and any condition diagnosed within the prior 10 years that are considered to be clinically significant by the Investigator.
- Any autoimmune disorders, regardless of onset date, should be recorded.

7.1.4. Prior and concomitant medications review

7.1.4.1. Prior Medications

The investigator or qualified designee will review prior medication use, including any protocol-specified washout requirement, and record prior medication taken by the subject within 30 days of first dose.

7.1.4.2. Concomitant Medications

- The investigator or qualified designee will record medication, if any, taken by the subject during the trial from the time of signing the informed consent form until the Safety Follow-up Visit.
- All medications related to reportable SAEs should be recorded.

7.1.5. Assignment of Screening Number

- All consented subjects will be given a unique screening number that will be used to identify the subject for all procedures that occur.
- Each subject will be assigned only one screening number. Screening numbers must not be re-used for different subjects. Any subject who is screened multiple times will retain the original screening number assigned at the initial screening visit.

7.1.6. Subject Identification Card

- All subjects will be given a Subject Identification Card identifying them as participants in a research trial. The card will contain trial site contact information (including direct telephone numbers) to be utilized in the event of an emergency.
- The investigator or qualified designee will provide the subject with a Subject Identification Card immediately after the subject provides written informed consent.

7.1.7. Submitting Tumor Sample

- Tumor tissue sample is mandatory to participate in this trial.
- Either pre-existing archived or newly-obtained (fresh tissue) biopsy specimens from either primary or metastatic tumor, whichever is most recent.
    - Newly-obtained (fresh tissue) is defined as a specimen obtained up to 42 days prior to administration of study treatment on Day 1 of Cycle 1, and no additional anti-cancer treatment has been given after the specimen was obtained.
    - Pre-existing, archived tissue must be obtained prior to time point that any anti-cancer treatment was given.

- A fine needle aspirate (FNA) or cytologic specimen will not be acceptable. In the event the most recent available tumor tissue specimen is an FNA or cytologic specimen, a previous specimen obtained prior to any anti-cancer therapy was given may be submitted (provided it is not an FNA or cytologic specimen).
- Where available, both newly-obtained (fresh tissue) and pre-existing archived tissues are requested.
- Tumor tissue specimen submitted in either formalin solution or FFPE block is acceptable.

7.1.8. Tumor Imaging

- To meet screening criteria, initial tumor imaging must be performed within 28 days prior to the beginning of the treatment. This scan will be considered the baseline assessment for the study. Scans performed as part of routine clinical management are acceptable for use as the baseline scan if they are of diagnostic quality, include all required anatomy, and performed within 28 days prior to the beginning of the treatment.
- The site study team must review pre-trial images to confirm the subject has at least one target lesion (i.e. meets measurability requirements) per RECIST 1.1.
- The following assessments are required at screening:

7.1.8.1. Computed (CT) with IV contrast or Magnetic Resonance Imaging (MRI) of chest, abdomen and pelvis.

- The preferred radiologic technique is CT with intravenous (IV) contrast. If a patient is known to have a contraindication to CT contrast or develops a contraindication during the trial, a non-contrast CT of the chest (MRI is not recommended due to respiratory artifacts) plus a contrast-enhanced MRI (if possible) of the abdomen and pelvis should be performed.

7.1.8.2. Brain CT with IV contrast or MRI scan

Patients with known baseline brain lesions are not eligible for this study, but brain CT scan or MRI is not required neither at baseline nor in the follow-up in the absence of neurological signs or symptoms.

7.1.8.3. Whole body bone scan

This is not required at any time point unless clinically indicated.

7.1.9. Laboratory Assessments

- Laboratory tests for screening should be performed within 7 days prior to study enrollment. If performed < 7 days prior to the first dose of trial therapy the screening laboratory tests will serve as cycle 1 day 1 laboratory tests. If not, laboratory tests will need to be performed again on day 1 of cycle 1.
    - Serum and plasma samples for optional biomarker studies will be collected prior to the beginning of treatment.

A summary of the procedures required is summarized in Table 1 and flow chart- Section 7.4.

7.1.10. 12-Lead Electrocardiogram (ECG)

A standard 12-lead ECG will be performed using local standard procedures once at screening. Clinically significant abnormal findings should be recorded as medical history and monitored if needed. No new ECGs will be performed unless clinically indicated.

7.2. During treatment

7.2.1. Adverse Event (AE) Monitoring

- The investigator or qualified designee will assess each subject to evaluate for potential new or worsening AEs as specified in the Trial Flow Chart and more frequently if clinically indicated.
- Adverse events will be graded and recorded throughout the trial and during the follow-up period according to NCI CTCAE Version 5.0. Toxicities will be characterized in terms regarding seriousness, causality, toxicity grading, and action taken with regard to trial treatment.
- All AEs of unknown etiology associated with nivolumab exposure should be evaluated to determine if it is possibly an ECI of a potentially immunologic etiology (termed immune-related adverse events, or irAEs).

7.2.2. Physical Exam

The investigator or clinical designee will perform a complete physical exam during the screening period. Clinically significant abnormal findings should be recorded as medical history.
Additional full physical exams should be performed as specified in the Trial Flow Chart-Section 7.4. After the first dose of trial treatment new clinically significant abnormal findings should be recorded as AEs.

7.2.3. Vital Signs

- The investigator or qualified designee will take vital signs at screening, prior to the administration of each dose of trial treatment and at treatment discontinuation as specified in the Trial Flow Chart-Section 7.4.
- Vital signs should include temperature, pulse, respiratory rate, weight and blood pressure. Height will be measured at screening only.

7.2.4. 12-Lead Electrocardiogram (ECG)

No ECGs will be performed unless clinically indicated.

7.2.5. Eastern Cooperative Oncology Group (ECOG) Performance Status

The investigator or qualified designee will assess ECOG status (see appendix) at screening, prior to dosing on day 1 of each treatment cycle and at discontinuation of trial treatment as specified in the Trial Flow Chart.

7.2.6. Tumor Imaging and Assessment of Disease

- Tumor imaging will be performed by whole body contrast-enhanced computed tomography (CT; preferred).
- The first on-study imaging assessment should be performed at 8 weeks (± 7 days) after the first dose of trial treatment. Subsequent imaging should be performed every 8 weeks (± 7 days) during the first 12 months and every 12 weeks thereafter until PD. Imaging may be more frequently performed if clinically indicated.

7.2.7. Laboratory Assessments

- After Cycle 1, pre-dose laboratory procedures can be conducted within 72 hours prior to dosing. Results must be reviewed by the investigator or qualified designee and found to be acceptable prior to each dose of trial treatment.
- The main laboratory determinations are summarized in Table 7

Table 7. Laboratory determinations required during the study

Test category	Determinations
Hematology	Hematocrit, Hemoglobin, Platelets, Red blood cells, White blood cells, Differential (Basophils, Eosinophils, Lymphocytes, Monocytes, Neutrophils, Bands, Other).
Chemistry	Albumin, Alkaline phosphatase, ALT, AST, Gamma-glutamyl-transferase (GGT), Lactate Dehydrogenase (LDH), Calcium, Magnesium, Phosphorus,, Sodium, Potassium, Creatinine, Creatine kinase, Direct Bilirubin, Indirect Bilirubin, Total Bilirubin,,, Total Protein, Blood Urea Nitrogen (BUN) or Urea, Uric Acid, Amylase, Lipase, Glucose.
Coagulation	**At Screening:** Prothrombin time (PT), International normalized ratio (INR]), Activated partial thromboplastin time (APTT).
Thyroid	**At screening**: TSH (Thyroid Stimulating Hormone), Free T3 and Free T4.<br>At the subsequent visits as indicated in the flow chart: TSH only.<br>If TSH is abnormal, Free T3 and T4 shall be tested.
Hepatitis markers	**At screening**: HBV-DNA, HBsAg, HBsAb, HBcAb, HCV RNA-PCR.
Cytokines	IFN-γ, IL-6, IL-1,TNF-α.
Tumor markers	**At screening**: NSE, CGA.<br>**On treatment:** every 2 cycles if elevated at baseline.
Urinalysis	**At Screening**: Macroscopic Panel (Dipstick) (Color, Bilirubin, Blood, Glucose, Ketones, Leukocytes esterase, Nitrite, pH, Protein, Specific Gravity, Urobilinogen)<br>If dipstick is abnormal then perform local laboratory Microscopic Panel (Red Blood Cells, White Blood Cells, Casts, Crystals, Bacteria, Epithelial cells).
Pregnancy test	A serum pregnancy test must be performed at screening (at the local laboratory) within ≤72 hours before first dose of study treatment.

7.2.8. Serum/Urine β-hCG

- All women who are being considered for participation in the trial, and who are not surgically sterilized or postmenopausal (defined as: a woman who is ≥45 years of age and has not had menses for more than 1 year), will be tested for pregnancy within 72 hours prior to each cycle of trial treatment and 30 days post treatment.
- Subjects must be excluded/discontinued in the event of a positive or borderline-positive test result. If a urine test is positive or borderline a serum β-HCG test will be required. The results of the pregnancy testing will not be recorded.

7.3. After the end of treatment (Follow-up)

- After the end of the treatment clinical procedures and assessment will be performed as previously explained and is reflected in the summary table (section 7.4).
- Serum and plasma samples for optional biomarker studies will also be collected at this point.

7.4. Summary table

Table 7.4. Trial flow chart

	**Screening phase**	**Treatment cycles [a] (chemotherapy + nivolumab)**						**Maintenance phase Cycles**		**End of treatment**	**Post-treatment**		
											Safety follow up	Follow up visits	Survival follow up [o]
	Pre-Study	1	2	3	4	5	6	1	2 and beyond	Time of treatment discontinuation	30 days after last dose	Every 12 w after last dose	Every 12w
Scheduling window (days)	-28 to -1		±3	±3	±3	±3	±3	±3	±3	±3	±7	±7	±7
Study Drug Admin[a]		X	X	X	X	X	X	X	X				
Cycle of Chemotherapy		X	X	X	X	X	X	X	X				
Informed consent [b]	X												
Informed consent for future biomedical research [b]	X												
History/Demographics [c]	X												
Physical exam [d]	X	X	X	X	X	X	X	X	X	X	X	X	X
Concomitant meds [e]	X	X	X	X	X	X	X	X	X	X	X		
ECOG - Performance status [f]	X	X	X	X	X	X	X	X	X	X	X		
Vital signs, Weight [g]	X	X (when clinically indicated)								x	x		
Adverse event evaluation [h]	X	X	X	X	X	X	X	X	X	X	X		
Tumor sample collection [i]	X												
Hematology [j]	X	X	X	X	X	X	X	X	X	X	X	X	X
Serum chemistry [j]	X	X	X	X	X	X	X	X	X	X	X	X	X
TSH, T3, FT4 [j]	X		X		X		X			X	X		
Hepatitis testing [j]	X	If clinically indicated, perform test as needed.											

	Screening phase	Treatment cycles [a] (chemotherapy + nivolumab)						Maintenance phase Cycles		End of treatment	Post-treatment		
	Pre-Study	1	2	3	4	5	6	1	2 and beyond	Time of treatment discontinuation	Safety follow up	Follow up visits	Survival follow up [o]
											30 days after last dose	Every 12 w after last dose	Every 12w
Cytokines [j]	X	Anytime when a suspected cytokine release syndrome occurs, immediately after the AE, and one week after occurrence of the AE											
Optional serum and plasma samples for biomedical research [K]	X									X			
Urinalysis [j]	X												
Serum or urine pregnancy test [l]	X												
EKG[m]	X												
Radiologic evaluation [n]	X	Treatment Period: Every 8 weeks counting from Cycle 1 Day 1 for 12 months, and every 12 weeks thereafter.<br>EOT: If a scan was not conducted within 30 days prior to end of study treatment<br>Efficacy follow-up: Continue same schedule as during treatment period until central review confirmed irRECIST progression.												

a) Unless otherwise specified, assessments/procedures are to be performed on Day 1 and prior to the first dose of treatment for each cycle.
b) Informed consent must be obtained before conducting any study-specific procedures (i.e. all of the procedures described in the protocol). The process of obtaining informed consent should be documented in the patient source documents. If a patient decides not to participate in the optional biomarker study, this in no way will affect the patient's ability to participate in the main research study.
c) All active conditions, and any condition diagnosed within the prior 10 years that are considered to be clinically significant by the Investigator. Any autoimmune disorders, regardless of onset date, should be recorded
d) Complete physical exam during the screening period. Clinically significant abnormal findings should be recorded as medical history. After the first dose of trial treatment new clinically significant abnormal findings should be recorded as AEs.
e) Site staff will review and record prior medication taken by the subject within 30 days of first dose.All concomitant medications received during the trial through 30 days after the last dose of trial treatment should be recorded.
f) ECOG status should be measured and registered at patient records and eCRF at screening, prior to dosing on day 1 of each treatment cycle and at discontinuation of trial treatment.
g) Vital signs should include temperature, pulse, respiratory rate, weight and blood pressure. Height will be measured at screening only.
h) Adverse events will be graded and recorded throughout the trial and during the follow-up period according to NCI CTCAE Version 5.0. Toxicities will be characterized in terms regarding seriousness, causality, toxicity grading, and action taken with regard to trial treatment.
i) Tumor tissue sample is mandatory to participate in this trial. It is accepted either pre-existing archived or newly-obtained (fresh tissue) biopsy specimens from either primary or metastatic tumor, whichever is most recent.
j) Laboratory tests for screening should be performed within 7 days prior to study enrollment. If performed < 7 days prior to the first dose of trial therapy the screening laboratory tests will serve as cycle 1 day 1 laboratory tests. If not, laboratory tests will need to be performed again on day 1 of cycle 1. Biochemistry includes: Albumin, Alkaline phosphatase, ALT, AST, Gamma-glutamyl-transferase (GGT), Lactate Dehydrogenase (LDH), Calcium, Magnesium, Phosphorus, Sodium, Potassium, Creatinine, Creatine kinase, Direct Bilirubin, Indirect Bilirubin, Total Bilirubin, Total Protein, Blood Urea Nitrogen (BUN) or Urea, Uric Acid, Amylase, Lipase, Glucose. The main laboratory determinations are summarized in Table 7 of the protocol.
k) Serum and plasma samples for optional biomarker studies will be collected prior to the beginning of treatment and at end of treatment.
l) Serum/Urine β-hCG A serum pregnancy test must be performed at screening (at the local laboratory) and when clinically indicated.
m) A standard 12-Lead Electrocardiogram (ECG) using local procedures at screening, additional ECGs will be performed only if clinically indicated.
n) The preferred radiologic technique is CT with intravenous (IV) contrast. If a patient is known to have a contraindication to CT contrast or develops a contraindication during the trial, a non-contrast CT of the chest (MRI is not recommended due to respiratory artifacts) plus a contrast-enhanced MRI (if possible) of the abdomen and pelvis should be performed. Brain CT scan or MRI or Whole Body Bone Scan are not required neither at baseline nor in the follow-up in the absence of neurological/bone signs or symptoms. Participants who discontinue study treatment in absence of disease progression, must continue their follow-up every 12 weeks (+/-7 days) by imaging until disease progression.
o) In this study, OS is a key endpoint of the study. Post-study follow-up is of critical importance and is essential. Participants who discontinue study treatment must continue their follow-up every 12 weeks (+/- 7 days), until death or the conclusion of the study (phone contacts are allowed).

8. Evaluation criteria

8.1. Definitions

- Evaluable for toxicity: All patients will be evaluable for toxicity from the time of their first treatment with nivolumab in combination with platinum-doublet chemotherapy.

- Evaluable for response: All eligible patients will be included in the response rate calculation. The subset that will be assigned a response category (Complete Response, Partial Response, Stable Disease or Progressive Disease; see definitions below) are all patients who have received at least one treatment and have their disease re-evaluated. Patients will have their response classified according to the definitions set out below *(Eisenhauer E, 2009)*.

8.2. Evaluation of efficacy

- Study evaluations will take place in accordance with the Schedule of Activities described in Section 7.4.
- Objective tumor response and time of progression will be measured according to RECIST criteria v.1.1.
- Response criteria are essentially based on a set of measurable lesions identified at baseline as target lesions, and – together with other lesions that are denoted as non-target lesions – followed until disease progression.

8.2.1. Measurability of tumour lesions at baseline

- Chest- abdominal and pelvic CT scan will be used to measure the lesions according to RECIST criteria.
- To meet screening criteria, initial tumor imaging must be performed within 28 days prior to the beginning of treatment. This scan will be considered the baseline assessment for the study. The site study team must review pre-trial images to confirm the subject has at least one target lesion (i.e. meets measurability requirements) per RECIST 1.1.
- irRECIST criteria will be used to measure ORR as secondary endpoint.

8.2.1.1. Definitions

- Measurable disease: the presence of at least one measurable lesion. If the measurable disease is restricted to a solitary lesion, its neoplastic nature should be confirmed by cytology/histology.
- Measurable lesions: *tumour lesions* that can be accurately measured in at least one dimension (longest diameter to be recorded) as ≥ 20 mm with chest x-ray, and as ≥ 10 mm with CT scan or clinical examination [using calipers]. Bone lesions are considered measurable only if assessed by CT scan and have an identifiable soft tissue component that meets these requirements (soft tissue component ≥ 10 mm by CT scan). *Malignant lymph nodes* must be ≥ 15 mm in the short axis to be considered measurable; only the short axis will be measured and followed. All tumour measurements must be recorded in millimeters (or decimal fractions of centimeters) by use of a ruler or calipers. Tumour lesions situated in a previously irradiated area, or in an area subjected to other locoregional therapy, are usually not considered measurable unless there has been demonstrated progression in the lesion. Provide detail on the conditions under which such lesions would be considered measurable.
- Non-measurable lesions: all other lesions (or sites of disease), including small lesions are considered non-measurable disease. Bone lesions without a measurable soft tissue component, leptomeningeal disease, ascites, pleural/pericardial effusions, lymphangitis cutis/pulmonis, inflammatory breast disease, lymphangitic involvement of lung or skin and abdominal masses followed by clinical examination are all non-measurable. Nodes that have a short axis <10 mm at baseline are considered non-pathological and should not be recorded or followed.

- Target Lesions: when more than one measurable tumour lesion or malignant lymph node is present at baseline all lesions up to *a maximum of 5 lesions total* (and a maximum of *2 lesions per organ*) representative of all involved organs should be identified as target lesions and will be recorded and measured at baseline. Target lesions should be selected on the basis of their size (lesions with the longest diameter), be representative of all involved organs, but in addition should be those that lend themselves to *reproducible repeated measurements*. Note that pathological nodes must meet the criterion of a short axis of ≥ 15 mm by CT scan and only the *short* axis of these nodes will contribute to the baseline sum. At baseline, the sum of the target lesions (longest diameter of tumour lesions plus short axis of lymph nodes: overall maximum of 5) is to be calculated and recorded.
- Non-target Lesions: all non-measurable lesions (or sites of disease) including pathological nodes (those with short axis ≥ 10 mm but < 15 mm), plus any measurable lesions over and above those listed as target lesions are considered *non-target lesions*. Measurements are not required but these lesions should be noted at baseline and should be followed as "present" or "absent".

8.2.1.2. Methods of measurements

- Patient must have measurable lesions on CT according to RECIST 1.1 in order to enter the study.
- The preferred radiologic technique is CT with IV contrast. If a patient is known to have a contraindication to CT contrast or develops a contraindication during the trial, a non-contrast CT of the chest (MRI is not recommended due to respiratory artifacts) plus a contrast-enhanced MRI (if possible) of the abdomen and pelvis should be performed.

8.2.2. Tumor response evaluation

8.2.2.1. Response evaluation according RECIST 1.1 [xliv]

- All patients will have their best response according to RECIST criteria 1.1 from the start of study treatment until the end of treatment classified as outlined below:
    - Complete or partial responses may be claimed only if the criteria for each are met at a subsequent time point at least 4 weeks later. Refer to the table below *(Table 8.2.2.1-a).*
    - Complete Response (CR): disappearance of all *target* and *non-target* lesions and normalization of tumour markers. Pathological lymph nodes must have short axis measures < 10 mm (Note: continue to record the measurement even if < 10 mm and considered CR). Tumour markers must have normalized. Residual lesions (other than nodes < 10 mm) thought to be non-malignant should be further investigated (by cytology or PET scans) before CR can be accepted.
    - Partial Response (PR): at least a 30% decrease in the sum of measures (longest diameter for tumour lesions and short axis measure for nodes) of target lesions, taking as reference the baseline sum of diameters. Non target lesions must be non-PD.
    - Stable Disease (SD): Neither sufficient shrinkage to qualify for PR nor sufficient increase to qualify for PD taking as reference the smallest sum of diameters on study.
    - Progressive Disease (PD): at least a 20% increase in the sum of diameters of measured lesions taking as references the smallest sum of diameters recorded on study (including baseline) AND an absolute increase of ≥ 5 mm. Appearance of new

lesions will also constitute PD (including lesions in previously unassessed areas). In exceptional circumstances, unequivocal progression of non-target disease may be accepted as evidence of disease progression, where the overall tumour burden has increased sufficiently to merit discontinuation of treatment, for example where the tumour burden appears to have increased by at least 73% in volume (which is the increase in volume when all dimensions of a single lesion increase by 20%). Modest increases in the size of one or more non-target lesions are NOT considered unequivocal progression. If the evidence of PD is equivocal (target or non-target), treatment may continue until the next assessment, but on further documentation, the earlier date must be used [xlv].

Table 8.2.2.1-a: Integration of target, non-target and new lesions into response assessment:

**Target Lesions**	**Non-Target Lesions**	**New Lesions**	**Overall Response**	**Best Response for this category also requires**
Patients with Target lesions ± non target lesions				
CR	CR	No	CR	Normalization of tumour markers, tumour nodes < 10 mm
CR	Non-CR/Non-PD	No	PR	
CR	Not all evaluated	No	PR	
PR	Non-PD/ not all evaluated	No	PR	
SD	Non-PD/ not all evaluated	No	SD	Documented at least once ≥ **4 weeks from baseline [note, protocol may define; 6-8 weeks is recommended]**
Not all evaluated	Non-PD	No	NE	
PD	Any	Any	PD	
Any	PD	Any	PD	
Any	Any	Yes	PD	
Patients with Non target lesions ONLY				
No Target	CR	No	CR	Normalization of tumour markers, all tumour nodes <10 mm
	Non-CR/Non-PD	No	Non-CR/Non-PD	
	Not all evaluated	No	NE	
	Unequivocal PD	Any	PD	
	Any	Yes	PD	
Note: Patients with a global deterioration of health status requiring discontinuation of treatment without objective evidence of disease progression [or evidence of unequivocal disease progression] at that time should be reported as "*symptomatic deterioration*". This is a reason for stopping therapy, but is NOT objective PD. Every effort should be made to document the objective progression even after discontinuation of treatment.				

The best overall response can be interpreted as below *(Table 8.2.2.1-b)*:

Table 8.2.2.1-b. Response on confirmation

**Response: First time point**	**Subsequent time point**	**BEST overall response**	**Also requires**
CR	CR	CR	Normalization of tumour markers, tumour nodes < 10 mm
CR	PR	SD, PD or PR (see comment*)	-
CR	SD	SD provided minimum criteria for SD duration met, otherwise, PD	-
CR	PD	SD provided minimum criteria for SD duration met, otherwise, PD	-
CR	NE	SD provided minimum criteria for SD duration met, otherwise NE	-
PR	CR	PR	-
PR	PR	PR	-
PR	SD	SD	-
PR	PD	SD provided minimum criteria for SD duration met, otherwise, PD	-
PR	NE	SD provide minimum criteria for SD duration met, otherwise, NE	-
NE	NE	NE	-
* May consider PR providing initial "CR" likely PR on subsequent review – then original CR should be corrected. Recurrence of lesion after true CR is PD.			

8.2.2.2. Response evaluation according irRECIST [xlvi]

- Following radiological assessment of PD by RECIST 1.1, sites will assess tumor response and progression per immune-related RECIST (irRECIST) for all subjects included in the trial.
- If imaging shows PD per RECIST 1.1, tumor assessment may be repeated at the site at least 4 weeks later to confirm PD (per irRECIST) with the option of continuing treatment until this scan is obtained for clinically stable subjects (see Table 11). Subjects who have unconfirmed disease progression (per irRECIST) may continue on treatment and follow the regular

imaging schedule intervals until documented disease progression, the start of new anti-cancer treatment, withdrawal of consent, death, or the end of the trial, whichever occurs first.

- In determining whether or not the tumor burden has increased or decreased, investigators should consider all target lesions as well as non-target lesions.
- Subjects that are deemed clinically unstable are not required to have repeat imaging for confirmation. If radiologic progression is confirmed by subsequent scan, it is recommended that the subject be discontinued from trial treatment unless, in the investigator's opinion, the subject is deriving benefit from treatment. Clinically stable subjects may continue to receive trial therapy. Clinically stable is defined as follows:
  - Absence of signs and symptoms indicating disease progression.
  - No decline in ECOG performance status.
  - Absence of rapid progression of disease.
  - Absence of progressive tumor at critical anatomical sites requiring urgent alternative medical intervention.

Table 8.2.2.2. Management after 1st radiological progression

	**Clinically stable**		**Clinically unstable**	
	**Imaging**	**Treatment**	**Imaging**	**Treatment**
1st Radiological evidence of PD per RECIST 1.1	Repeat imaging at ≥ 4 weeks to confirm PD.	May continue treatment at investigator's discretion while waiting for confirmatory scan.	Repeat imaging at ≥ 4 weeks to confirm PD if possible.	Discontinue treatment.
Repeat scan confirms PD	No additional imaging required	Discontinue treatment.	No additional imaging required	
Repeat scan shows SD, PR or CR.	Continue regularly schedule imaging every 8 weeks.	Continue study treatment at investigator's discretion.	Continue regularly schedule imaging every 8 weeks.	May restart study treatment as per investigator´s discretion.

8.2.2.3. Frequency of tumor re-evaluation

- The first on-study imaging assessment should be performed at 12 weeks (± 7 days) after the first dose of trial treatment. Subsequent imaging should be performed every 12 weeks (± 7 days) until PD.
- Imaging should be repeated at least 4 weeks after the first observation of a complete or partial response.
- Imaging may be more frequent if clinically indicated. Imaging should not be delayed for delays in cycle starts.
- After discontinuation of protocol treatment, patients who have not progressed will still be re-evaluated every 12 weeks.

8.2.3. Reporting of tumor response

All patients included in the study must be assessed for response to treatment, even if there is a major protocol treatment deviation or if they are ineligible, or not followed/re-evaluated. Each patient will be assigned one of the following categories: complete response, partial response, stable disease, progressive disease, early death from malignant disease, early death from toxicity, early death from other cause or unknown (not assessable, insufficient data).

Early death is defined as any death occurring before the first per protocol time point of tumor re-evaluation. The responsible investigator will decide if the cause of death is malignant disease, toxicity or other cause.

Patients for whom response is not confirmed will be classified as "unknown", unless they meet the criteria for stable disease (or the criteria for partial response in case of an unconfirmed complete response). Patients' response will also be classified as "unknown" if insufficient data were collected to allow evaluation per these criteria. Please refer to table *8.2.2.1-b.*

8.2.3.1. Response duration

Response duration will be measured from the time measurement criteria for CR/PR (whichever is first recorded) are first met until the first date that recurrent or progressive disease is objectively documented.

8.2.3.2. Stable disease duration

Stable disease duration will be measured from the time of start of treatment (or randomization for randomized studies) until the criteria for progression are met.

8.2.3.3. Progression Free Survival

Progression free survival will be calculated from the date treatment with first-line chemotherapy and nivolumab begins until the date of first progressive disease as per RECIST 1.1 or death.

8.2.3.4. Overall Survival

Overall survival will be calculated from the date treatment with first-line chemotherapy and nivolumab begins until the date of death from any cause.

9. Statistical considerations

9.1. Statistical design

9.1.1. Sample Size Determination

This is a phase II open-label, single arm study. The primary endpoint is the one-year overall survival rate.

The sample size of this study is driven by its primary objective which is to determine the one-year OS rate calculated from the date treatment with nivolumab and platinum-based chemotherapy begins, until the date of death from any cause in patients with advanced G3 NENs of

GEP tract or unknown primary site, who have not received any prior antineoplastic systemic treatment.

Based on data derived from tumor registries[xlvii],[xlviii], median OS for these patients is approximately 11-12 months, with 1-year overall survival rate of 50%. The primary hypothesis (H1) is that chemotherapy in combination with nivolumab is superior to chemotherapy in terms of OS, achieving a median OS of 14 months, with 72% patients alive at 12 months. The study will be considered successful if the primary hypothesis is statistically significant. Considering the following assumptions: a power of 80%, alpha error of 0.05, 18 months accrual time and 12-month follow-up period, a total number of 38 patients should be included in the study, considering a drop out rate of 10%.

9.2. Populations for Analysis

For purpose of analysis, the following populations are defined:

Population	Description
Enrolled	All participants who signed informed consent and were registered.
Treated	All participants who received at least one dose of any study medication. This is the primary dataset for dosing and safety analysis.
Response-Evaluable	All treated subjects who have a baseline and at least one on-treatment imaging evaluation or had progression or death prior to the first on-treatment scan.

9.3. Statistical Analysis

9.3.1. Efficacy Analysis

Endpoint	Statistical Analysis Methods
Primary	Primary endpoint is one year OS rate with nivolumab plus chemotherapy. This is defined as the proportion of patients that remain alive at 12 months since the beginning of treatment. OS will be censored on the last date a participant was known to be alive.
Secondary	PFS and OS with nivolumab plus chemotherapy assessed by Central Review.<br>ORR with nivolumab plus chemotherapy assessed by Central Review.<br>Chromogranin A and enolase levels and their association with ORR, PFS, and OS.<br>**ORR** is defined as the number of participants with a best overall response of confirmed CR or PR divided by the number of enrolled participants. BOR is defined as the best response designation, as determined by the central review, recorded between the date of treatment start and the date of objectively documented progression per RECIST 1.1 or, the date of initiation of subsequent anti-cancer therapy, whichever occurs first. For participants without documented progression or subsequent anti-cancer therapy, all available response designations will contribute to the BOR determination. For participants who continue treatment beyond progression, the BOR will be determined based on response designations recorded up to the time of the initial RECIST 1.1-defined progression. Complete or partial responses may be claimed only if the criteria for each are met at a subsequent time point of ≥ 4 weeks later.

9.3.1.1. Methods for Primary Endpoint

The efficacy analysis will be performed for the treatment population.

For the primary endpoint, the OS rate at 12 months with 95% CIs will be estimated using Kaplan-Meier methodology if follow-up requirement is met. OS curves, medians with 95% CIs and OS rates at 6, 18 and 24 months with 95% CIs will be estimated if follow-up requirement is met. The proportion will be calculated by the product limit method (Kaplan-Meier [K-M] estimate), which takes into account censored data.

9.3.1.2. Methods for Secondary Endpoints

The efficacy analysis for secondary endpoints will be performed for all treated patients.

PFS is defined as the time from the randomization date to the date of the first documented tumor progression per RECIST 1.1, or death due to any cause.

Participants who did not progress or die will be censored on the date of their last evaluable tumor assessment. Participants who did not have any on study tumor assessments will be censored on the beginning of treatment date. PFS curves, PFS medians with 95% CIs, and PFS rates at 6, 12, 18, 24, 36, and 48 months with 95% CIs will be estimated using Kaplan-Meier methodology if follow-up requirements are met.

ORR is defined as the number of participants with a best overall response (BOR) of confirmed CR or PR divided by the number of enrolled participants. BOR is defined as the best response designation, as determined by central review, recorded between the date of treatment start and the date of objectively documented progression per RECIST 1.1 or, the date of initiation of subsequent anti-cancer therapy, whichever occurs first. For participants without documented progression or subsequent anti-cancer therapy, all available response designations will contribute to the BOR determination. For participants who continue treatment beyond progression, the BOR will be determined based on response designations recorded up to the time of the initial RECIST 1.1-defined progression. Complete or partial responses may be claimed only if the criteria for each are met at a subsequent time point of ≥ 4 weeks later.
An estimate of the response rate and an associated exact two-sided 95% CI will be presented.

9.3.2. Safety Analysis

Safety analysis will be performed in all treated participants.

Descriptive statistics of safety will be presented using National Cancer Institute (NCI) Common Terminology Criteria for Adverse Events (CTCAE) version 5.0. All on-study AEs, drug related AEs, SAEs and drug-related SAEs, AEs and drug-related AEs leading to drug discontinuation will be tabulated using worst grade per NCI CTCAE v 5.0 criteria by system organ class and preferred term, based on MedDRA terminology. On-study lab parameters including hematology, coagulation, chemistry, liver function and renal function will be summarized using worst grade per NCI CTCAE v 5.0 criteria.

A tabular summary of the incidence of overall immune-mediated adverse events (imAEs) and serious imAEs will be performed. Frequency, management and resolution of imAEs will be analyzed and a descriptive analysis including time-to-onset, severity, duration, action taken with the study drug,

dosing delays of the study drug, corticosteroid details, re-challenge information and outcome of the AE will be individually characterized by imAE category.

10. Translational research

Baseline tumor samples will be mandatory prior to the beginning of the study. Serial blood samples (including serum and plasma at baseline and at disease progression or EOT) will be collected for additional biomarker research to identify prognostic and/or predictive factors of response to nivolumab and carboplatin-etoposide chemotherapy.

Biological samples may undergo genomic, metabolomic, transcriptomic and proteomic analyses or other molecular assessments related to the disease, the immunological landscape of tumors and patients, or the mechanism of action of drugs tested with this purpose. Additional information on this subject is available for all participants in the informed consent form.

The samples will be sent to and stored at the Gastrointestinal Tumors Research Unit from the Research Institute at the Hospital Universitario 12 de Octubre, Madrid.

11. Patient registration procedure

- Each patient is identified in the study by a Subject Number (Subject No.) that is assigned when the patient is first enrolled for screening and is retained as the primary identifier for the patient throughout his/her entire participation in the trial. The Subject No. consists of the Center Number (Center No.) with a sequential patient number suffixed to it, so that each patient is numbered uniquely across the entire database.
- Upon signing the informed consent form, the patient is assigned to the next sequential Subject No. available to the investigator.
- The investigator or designated staff will contact the IRT and provide the requested identifying information for the patient to register them into the IRT. Once assigned, the Subject No. must not be reused for any other patient and the Subject No. for that individual must not be changed, even if the patient is re-screened. If the patient fails to start treatment for any reason, the reason will be entered into the Screening Disposition page.

12. Reporting adverse events

12.1. Definitions for adverse event reporting

- An **Adverse Event (AE)** is defined as any untoward medical occurrence or experience in a patient or clinical investigation subject which occurs following the administration of the trial medication regardless of the dose or causal relationship. This can include any unfavorable and unintended signs (such as rash or enlarged liver), or symptoms (such as nausea or chest pain), an abnormal laboratory finding (including blood tests, x-rays or scans) or a disease temporarily associated with the use of the protocol treatment. *(ICH-GCP).*
- An **Adverse Drug Reaction (ADR)** is defined as any response to a medical product, that is noxious and/or unexpected, related to any dose. *(ICH-GCP). Response to a medicinal product* (used in the above definition) means that a causal relationship between the medicinal product

and the adverse event is at least a reasonable possibility, i.e. the relationship cannot be ruled out.

- An **Unexpected Adverse Drug Reaction** is any adverse reaction for which the nature or severity is not consistent with the applicable product information (e.g., Investigator's Brochure). *(ICH-GCP).*
- A **Serious Adverse Event (SAE)** is defined as any undesirable experience occurring to a patient, whether or not considered related to the protocol treatment. A Serious Adverse Event (SAE) which is considered related to the protocol treatment is defined as a **Serious Adverse Drug Reaction (SADR)**.
- Adverse events and adverse drug reactions which are considered as **serious** are those which result in:
    - Death
    - A life threatening event (i.e. the patient was at immediate risk of death at the time the reaction was observed)
    - hospitalization or prolongation of hospitalization
    - persistent or significant disability/incapacity
    - a congenital anomaly/birth defect
    - any other medically important condition (i.e. important adverse reactions that are not immediately life threatening or do not result in death or hospitalization but may jeopardize the patient or may require intervention to prevent one of the other outcomes listed above)

12.2. Reporting procedure

To ensure patient safety, every SAE, regardless of suspected causality, occurring after the patient has provided informed consent must be reported within 24 hours of knowing of its occurrence and until:

- Suspected SAEs up to Day 150 safety follow-up.
- Non-suspected SAEs up to Day 150 safety follow-up or start of new post treatment antineoplastic medication if administered during the period between the 30-Day safety follow-up and 150-Day safety follow-up, whichever is sooner.
- If a patient starts a post treatment antineoplastic therapy after the 30-day safety follow-up, then only SAEs suspected to be related to study treatment should be collected out to 150 days after discontinuation of treatment. SAEs suspected to be related to nivolumab will continue to be collected beyond the 150-Day safety visit.
- Any additional information for the SAE including complications, progression of the initial SAE, and recurrent episodes must be reported as follow-up to the original episode within 24 hours of the investigator receiving the follow-up information. A SAE occurring at a different time interval or otherwise considered completely unrelated to a previously reported one should be reported separately as a new event.
- Any SAEs experienced after the reporting period described above should only be reported if the investigator suspects a causal relationship to the study treatment.
- Information about all SAEs is collected and recorded on the Serious Adverse Event Report Form; all applicable sections of the form must be completed in order to provide a clinically thorough report. The investigator must assess and record the relationship of each SAE to each specific study treatment (if there is more than one study treatment).

- Each reoccurrence, complication, or progression of the original event should be reported as a follow-up to that event regardless of when it occurs. The follow-up information should describe whether the event has resolved or continues, if and how it was treated, and whether the patient continued or withdrew from study participation.
- Suspected Unexpected Serious Adverse Reactions (SUSARs) will be collected and reported to the competent authorities and relevant ethics committees in accordance with Directive 2001/20/EC or as per national regulatory requirements in participating countries.
- SAEs, whether related or not related to study drug, and pregnancies must be reported to BMS within 24 hours \ 1 Business Day of becoming aware of the event. SAEs must be recorded on either CIOMS, MedWatch, or approved site SAE form.
- Pregnancies must be reported and submitted to MFAR Clinical Research that will act on behalf of GETNE transferring the information to BMS. BMS will perform due diligence follow-up using the BMS Pregnancy Form which the investigator must complete.

  [redacted]
- If only limited information is initially available, follow-up reports are required. (Note: Follow-up SAE reports should include the same investigator term(s) initially reported.).
- If an ongoing SAE changes in its intensity or relationship to study drug or if new information becomes available, a follow-up SAE report should be sent within 24 hours \ 1 Business Day to BMS using the same procedure used for transmitting the initial SAE report.
- All SAEs should be followed to resolution or stabilization.

SAEs, whether related or not related to study drug, and pregnancies must be reported to MFAR Clinical Research that will manage, on behalf GETNE, the communications with BMS within 24 hours \ 1 Business Day of becoming aware of the event. SAEs must be recorded on either CIOMS, MedWatch, or approved site SAE form.

The causal relationship to study drug is determined by a physician and should be used to assess all adverse events (AE). The casual relationship can be one of the following:
- ***Related:*** There is a reasonable causal relationship between study drug administration and the AE.
- ***Not related:*** There is not a reasonable causal relationship between study drug administration and the AE.

The causal relationship to study drug is determined by a physician and should be used to assess all adverse events (AE). The casual relationship can be one of the following:
- ***Related:*** There is a reasonable causal relationship between study drug administration and the AE.
- ***Not related:*** There is not a reasonable causal relationship between study drug administration and the AE.
- The term "reasonable causal relationship" means there is evidence to suggest a causal relationship.
- Adverse events can be spontaneously reported or elicited during open-ended questioning, examination, or evaluation of a subject. (In order to prevent reporting bias, subjects should not be questioned regarding the specific occurrence of one or more AEs.)

NON SERIOUS ADVERSE EVENT

Non-serious Adverse Events (AE) are to be provided to BMS in aggregate via interim or final study reports as specified in the agreement or, if a regulatory requirement [eg, IND US trial] as part of an annual reporting requirement.

The Sponsor will reconcile the clinical database AE cases (case level only) transmitted to BMS Global Pharmacovigilance [redacted]

Non-serious Adverse Event Collection and Reporting

The collection of non-serious AE information should begin following the subject's written consent to participate in the study. All non serious adverse events (not only those deemed to be treatment-related) should be collected continuously during the treatment period and for a minimum of (provide # of days depending on the asset and study type) days following the last dose of study treatment.

Non-serious AEs should be followed to resolution or stabilization, or reported as SAEs if they become serious. Follow-up is also required for non-serious AEs that cause interruption or discontinuation of study drug and for those present at the end of study treatment as appropriate.

Laboratory Test Abnormalities

All laboratory test results captured as part of the study should be recorded following institutional procedures. Test results that constitute SAEs should be documented and reported to BMS as such.

The following laboratory abnormalities should be documented and reported appropriately:

- any laboratory test result that is clinically significant or meets the definition of an SAE.
- any laboratory abnormality that required the participant to have study drug discontinued or interrupted.
- any laboratory abnormality that required the subject to receive specific corrective therapy.

It is expected that wherever possible, the clinical rather than laboratory term would be used by the reporting investigator (eg, anemia versus low hemoglobin value).

Potential Drug Induced Liver Injury (DILI) Language required as noted below:

Definition of DILI criteria is mandatory for all pre-marketed asset protocols enrolling participants without known abnormalities in liver function at baseline AND for protocols involving participants with known liver abnormalities at baseline or with other clinical confounders where asset specific criteria for potential drug induced liver injury have been defined. Use for marketed assets is optional.

For protocols without known abnormalities in liver function at baseline, use the mandatory standard DILI definition listed below: Wherever possible, timely confirmation of initial liver related laboratory abnormalities should occur prior to the reporting of a potential DILI event. All occurrences of potential DILIs, meeting the defined criteria, must be reported as SAEs.

Potential drug induced liver injury is defined as:

1. AT (ALT or AST) elevation > 3 times upper limit of normal (ULN) AND
2. Total bilirubin > 2 times ULN, without initial findings of cholestasis (elevated serum alkaline phosphatase) AND
3. No other immediately apparent possible causes of AT elevation and hyperbilirubinemia, including, but not limited to, viral hepatitis, pre-existing chronic or acute liver disease, or the administration of other drug(s) known to be hepatotoxic.

AEs of Special Interest (Product Specific Usually a regulatory requirement. Remove if not applicable)

Pregnancy

If, following initiation of the investigational product, it is subsequently discovered that a study participant is pregnant or may have been pregnant at the time of investigational product exposure, including during at least 5 half-lives after product administration, the investigational product will be permanently discontinued in an appropriate manner (eg, dose tapering if necessary for participant).

The investigator must immediately notify [redacted] of this event and complete one of the following forms within 24 hours of awareness of the event via either the CIOMS, MedWatch or appropriate Pregnancy Surveillance Form in accordance with SAE reporting procedures.

Protocol-required procedures for study discontinuation and follow-up must be performed on the participant.

Follow-up information regarding the course of the pregnancy, including perinatal and neonatal outcome and, where applicable, offspring information must be reported on the CIOMS, MedWatch, BMS Pregnancy Surveillance Form, or approved site SAE form. A BMS Pregnancy Surveillance Form may be provided upon request.

Any pregnancy that occurs in a female partner of a male study participant should be reported to BMS. Information on this pregnancy will be collected on the Pregnancy Surveillance Form. In order for Sponsor or designee to collect any pregnancy surveillance information from the female partner, the female partner must sign an informed consent form for disclosure of this information.

Other Safety Considerations

Any significant worsening noted during interim or final physical examinations, electrocardiograms, X-rays, and any other potential safety assessments, whether or not these procedures are required by the protocol, should also be recorded as a non-serious or serious AE, as appropriate, and reported accordingly.

MFAR Clinical Research on behalf of GETNE will request from BMS GPV&E, [redacted] the SAE reconciliation report and include the BMS protocol number every 3 months and prior to database lock or final data summary.

GPV&E will send the investigator the report to verify and confirm all SAEs have been transmitted to BMS GPV&E.

The data elements listed on the GPV&E reconciliation report will be used for case identification purposes. If the Sponsor determines a case was not transmitted to BMS GPV&E, the case should be sent immediately to BMS [redacted]

13. Quality assurance

13.1. Control of data consistency

The investigator or qualified designee is responsible for recording and verifying the accuracy of subject data.

- The study will use eCRFs. A designated CRO will review the data entered by investigational staff for completeness and accuracy. Electronic data queries stating the nature of the problem and requesting clarification will be created for discrepancies and missing values and sent to the investigational site via the EDC system. Designated investigator site staff is required to respond promptly to queries and to make any necessary changes to the data.
- Concomitant treatments and prior medications entered into the database will be coded using the WHO Drug Reference List, which employs the Anatomical Therapeutic Chemical classification system.
- Medical history/current medical conditions and adverse events will be coded using the Medical dictionary for regulatory activities (MedDRA) terminology.
- The site staff designated by the investigator will enter the information required by the protocol onto the eCRFs as well as onto the designated CRO's requisition form.

13.2. On-site quality control (for multi-centre studies only when data are sent to a Data Centre or similar)

- Before study initiation, at a site initiation visit or at an investigator's meeting, the protocol and CRFs will be reviewed with the investigators and their staff.
- During the study, the field monitor will visit the site regularly to check the completeness of patient records, the accuracy of entries on the CRFs, the adherence to the protocol to Good Clinical Practice, the progress of enrollment, and to ensure that study treatment is being stored, dispensed, and accounted for according to specifications. Key study personnel must be available to assist the field monitor during these visits.
- The investigator must maintain source documents for each patient in the study, consisting of case and visit notes (hospital or clinic medical records) containing demographic and medical information, laboratory data, electrocardiograms, and the results of any other tests or assessments. All information recorded on CRFs must be traceable to source documents in the patient's file. The investigator must also keep the original signed informed consent form (a signed copy is given to the patient).
- The investigator must give the monitor access to all relevant source documents to confirm their consistency with the CRF entries.

13.3. Audits (for multi-centre studies only when data are sent to Data Centre or similar)

- To ensure quality of data, study integrity, and compliance with the protocol and the various applicable regulations and guidelines, the "Sponsor" may conduct site visits to institutions participating to protocols.
- The investigator, by accepting to participate to this protocol, agrees to cooperate fully with any quality assurance visit undertaken by third parties, including representatives from the "Sponsor", national and/or foreign regulatory authorities or company supplying the product under investigation, as well as to allow direct access to documentation pertaining to the clinical trial (including CRFs, source documents, hospital patient charts and other study files) to these authorized individuals.
- The investigator must inform the "Sponsor" immediately in case a regulatory authority inspection would be scheduled.

13.4. Central review of pathology

No central pathology review is planned.

13.5 Central review of Images

All images of patients will be uploaded to a platform in DICOM format and will be reviewed centrally by a radiologist specialised in pathology to avoid bias. Information for test uploading and management is provided in a central reviewing manual located at the ISF.

14. Ethical considerations

14.1. Patient protection

This protocol has been written, and the study will be conducted according to the ICH Harmonized Tripartite Guideline for Good Clinical Practice (ref: http://www.ifpma.org/pdfifpma/e6.pdf) with applicable local regulations (including European Directive 2001/20/EC and US Code of Federal Regulations Title 21), and with the ethical principles laid down in the Declaration of Helsinki.

The protocol and the proposed informed consent form must be reviewed and approved by a properly constituted Institutional Review Board/Independent Ethics Committee/Research required to sign a protocol signature page confirming his/her agreement to conduct the study in accordance with these documents and all of the instructions and procedures found in this protocol and to give access to all relevant data and records to auditors, Quality Assurance representatives, IRBs/IECs/REBs and regulatory authorities as required.

14.2. Subject identification

- The investigator must ensure anonymity of the patients; patients must not be identified by names in any documents submitted to the Sponsor.
- Signed informed consent forms and patient enrollment log must be kept strictly confidential to enable patient identification at the site.
- The investigator agrees that the IRB/ERC, or regulatory authority representatives may consult and/or copy trial documents in order to verify worksheet/case report form data. By signing the consent form, the subject agrees to this process. If trial documents will be photocopied during the process of verifying worksheet/case report form information, the subject will be identified by unique code only; full names/initials will be masked prior to transmission.
- By signing this protocol, the investigator agrees to treat all subject data used and disclosed in connection with this trial in accordance with all applicable privacy laws, rules and regulations.

14.3. Informed consent

- Eligible patients may only be included in the study after providing written (witnessed, where required by law or regulation), IRB/IEC/REB-approved informed consent.

- Informed consent must be obtained before conducting any study-specific procedures (i.e. all of the procedures described in the protocol). The process of obtaining informed consent should be documented in the patient source documents.
- The date when a patient's Informed Consent was actually obtained will be captured in their CRFs.
- An informed consent form (ICF) that is considered appropriate for this study and complies with the ICH GCP guideline and regulatory requirements will be provided with this protocol.
- Women of childbearing potential should be informed that taking the study medication may involve unknown risks to the fetus if pregnancy were to occur during the study and agree that in order to participate in the study they must adhere to the contraception requirement for the duration of the study. If there is any question that the patient will not reliably comply, they should not be entered in the study.

Additional consent form

Sub-studies and studies with an optional biomarker component may have a separate consent form covering those studies. This form will be adapted for each study. These informed consent forms will be submitted for ethical approval together with the Study Protocol and the main informed consent form of the study. If a patient opts not to participate in the optional assessments, this in no way will affect the patient's ability to participate in the main research study.

15. Publication policy

This trial is intended for publication, even if terminated prematurely. Publication may include any or all of the following: posting of a synopsis online, abstract and/or presentation at a scientific conference, or publication of a full manuscript.

The authors will work to submit a manuscript describing trial results within 12 months after the last data become available.

A synopsis of trial results for approved products will be posted on www.clinicaltrials.gov by 12 months after the last subject's last visit for the primary outcome, 12 months after the decision to discontinue development, or product marketing (dispensed, administered, delivered or promoted), whichever is later.

These timelines may be extended, if additional time is needed for analysis, to protect intellectual property, or to comply with confidentiality agreements with other parties.

Authors of the primary results manuscript will be provided the complete results from the Clinical Study Report, subject to the confidentiality agreement.

When a manuscript is submitted to a biomedical journal the protocol and statistical analysis plan will also be submitted to facilitate the peer and editorial review of the manuscript. If the manuscript is subsequently accepted for publication the journal will be allowed, to post on its website the key sections of the protocol that are relevant to evaluating the trial, specifically those sections describing the trial objectives and hypotheses, the subject inclusion and exclusion criteria, the trial design and procedures, the efficacy and safety measures, the statistical analysis plan, and any amendments relating to those sections.

The first author is responsible for defending the integrity of the data, method(s) of data analysis and the scientific content of the manuscript.

APPENDICES

Appendix A. Performance Status Criteria

**WHO Performance Status Scale**		**Karnofsky Performance Scale**	
**Grade**	Descriptions	**Percent**	Description
0	Normal activity. Fully active, able to carry on all pre-disease performance without restriction.	100	Normal, no complaints, no evidence of disease.
		90	Able to carry on normal activity; minor signs or symptoms of disease.
1	Symptoms, but ambulatory. Restricted in physically strenuous activity, but ambulatory and able to carry out work of a light or sedentary nature (e.g., light housework, office work)	80	Normal activity with effort; some signs or symptoms of disease.
		70	Cares for self, unable to carry on normal activity or to do active work.
2	In bed <50% of the time. Ambulatory and capable of all self-care, but unable to carry out any work activities. Up and about more than 50% of waking hours.	60	Requires occasional assistance, but is able to care for most of his/her needs.
		50	Requires considerable assistance and frequent medical care.
3	In bed >50% of the time. Capable of only limited self-care, confined in bed or chair more than 50% of waking hours.	40	Disabled, requires special care and assistance.
		30	Severely disabled, hospitalization indicated. Death not imminent.
4	100% bedridden. Completely disabled. Cannot carry on any self-care. Totally confined to bed or chair	20	Very sick, hospitalization indicated. Death not imminent.
		10	Moribund, fatal processes progressing rapidly.
5	Deceased.	0	Deceased.

Appendix B. References

[i] Garcia-Carbonero R , Capdevila J, Crespo-Herrero G et al. Incidence, patterns of care and prognostic factors for outcome of gastroenteropancreatic neuroendocrine tumors (GEP-NETs): results from the National Cancer Registry of Spain (RGETNE). Ann Oncol. 2010 Sep;21(9):1794-803.

[ii] Yao JC, Hassan M, Phan A, et al. One hundred years after "carcinoid": epidemiology of and prognostic factors for neuroendocrine tumors in 35,825 cases in the United States. J Clin Oncol. 2008 Jun 20;26(18):3063-72

[iii] Rindi G, Arnold R, Bosman FT, et al. Nomenclature and classification of neuroendocrine neoplasms of the digestive system. WHO classification of tumours of the digestive system. 4th ed Lyon: International Agency for Research on cancer (IARC); 2010.

[iv] Sorbye H, Strosberg J, Baudin E, et al. Gastroenteropancreatic high-grade neuroendocrine carcinoma. Cancer 2014;120: 2814-23.

[v] Sorbye H, Welin S, Langer SW et al. Predictive and prognostic factors for treatment and survival in 305 patients with advanced gastrointestinal neuroendocrine carcinoma (WHO G3): the NORDIC NEC study. Ann Oncol, 2013. 24(1): p. 152-60.

[vi] Moertel CG, Kvols LK, O'Connell MJ et al. Treatment of neuroendocrine carcinomas with combined etoposide and cisplatin. Evidence of major therapeutic activity in the anaplastic variants of these neoplasms. Cancer, 1991. 68(2): p. 227-232.

[vii] Mitry E, Baudin E, Ducreux M et al. Treatment of poorly differentiated neuroendocrine tumours with etoposide and cisplatin. Br J Cancer, 1999. 81(8): p. 1351.

[viii] Iwasa S, Morizane C, Okusaka T et al. Cisplatin and etoposide as first-line chemotherapy for poorly differentiated neuroendocrine carcinoma of the hepatobiliary tract and pancreas. Jpn J Clin Oncol, 2010. 40(4): p. 313-318.

[ix] Deutschbein T, Unger N, Yuece A et al. Chemotherapy in patients with progressive, undifferentiated neuroendocrine tumors: a single-center experience. Horm metab Res, 2011. 43(12): p. 838-843.

[x] Patta A, Fakih M. First-line cisplatin plus etoposide in high-grade metastatic neuroendocrine tumors of colon and rectum (MCRC NET): review of 8 cases. Anticancer Research, 2011. 31(3): p. 975-978.

[xi] Hanahan D, Weinberg RA. Hallmarks of cancer: the next generation. Cell 2011 Mar 4;144(5):646-74.

[xii] Pardoll DM. The blockade of immune checkpoints in cancer immunotherapy. Nat Rev Cancer 2012;12 (24):252-264.

[xiii] Borghaei H, Paz-Ares L, Horn L, et al. Nivolumab versus Docetaxel in Advanced Nonsquamous Non-Small-Cell Lung Cancer. N Engl J Med. 2015 Oct 22;373 (17):1627-39.

[xiv] Robert C, Long GV, Brady B, et al. Nivolumab in previously untreated melanoma without BRAF mutation. N Engl J Med. 2015 Jan 22;372 (4):320-30.

[xv] Hamid O, Carvajal RD. Anti-programmed death-1 and anti-programmed death-ligand 1 antibodies in cancer therapy. Expert Opin Biol Ther. 2013 Jun;13(6):847-61.

[xvi] Motzer RJ, Escudier B, McDermott DF, et al. Nivolumab versus Everolimus in Advanced Renal-Cell Carcinoma. N Engl J Med. 2015 Nov 5;373(19):1803-13.

[xvii] Goh G, Walradt T, Markarov V, et al. Mutational landscape of MCPyV-positive and MCPyV-negative merkel cell carcinomas with implications for immunotherapy. Oncotarget. 2015 Dec 7. doi: 10.18632/oncotarget.6494

[xviii] Talmadge JE, Donkor M, Scholar E. Inflammatory cell infiltration of tumors: Jekyll or Hyde. Cancer Metastasis Rev 2007; 26:373-400.

[xix] Hiraoka N. Tumor-infiltrating lymphocytes and hepatocellular carcinoma: molecular biology. Int J Clin Oncol 2010; 15:544-51.

[xx] Lee HE, Chae SW, Lee YJ, et al. Prognostic implications of type and density of tumour-infiltrating lymphocytes in gastric cancer. Br J Cancer 2008;99(10):1704-11.

[xxi] Leffers N, Gooden MJM, de Jong RA, et al. Prognostic significance of tumor-infiltrating T-lymphocytes in primary and metastatic lesions of advanced stage ovarian cancer. Cancer Immunol Immunother 2009; 58:449-59.

[xxii] Wolchok JD, Hoos A, O'Day S, et al. Guidelines for the evaluation of immune therapy activity in solid tumors: immune-related response criteria. Clin Cancer Res. 2009;15:7412-20.

[xxiii] Brahmer JR, Drake CG, Wollner I, et al. Phase I Study of Single-Agent Anti–Programmed Death-1 (MDX-1106) in Refractory Solid Tumors: Safety, Clinical Activity, Pharmacodynamics, and Immunologic Correlates. J Clin Oncol 2010; 28:3167-3175.

[xxiv] Opdivo Prescribing Information,2015.

[xxv] Antonia SJ, Lopez-Martin JA, Bendell J, et al. Nivolumab alone and nivolumab plus ipilimumab in recurrent small-cell lung cancer (CheckMate 032): a multicentre, open-label, phase 1/2 trial. Lancet Oncol. 2016;17(7):883–95.

[xxvi] Hellmann MD, Ott PA, Zugazagoitia J, Ready NE, et al. Nivolumab (nivo) ± ipilimumab (ipi) in advanced small-cell lung cancer (SCLC): first report of a randomized expansion cohort from CheckMate 032. J Clin Oncol. 2017;35(15_suppl):8503.

[xxvii] M. Reck, D. Vicente, T. Ciuleanu et al. Efficacy and safety of nivolumab (nivo) monotherapy versus chemotherapy (chemo) in recurrent small cell lung cancer (SCLC): Results from CheckMate 331. Annals of Oncology (2018) 29 (suppl_10): x39-x43. 10.1093/annonc/mdy511.

[xxviii] https://news.bms.com/press-release/corporatefinancial-news/bristol-myers-squibb-announces-checkmate-451-study-did-not-mee. Press release: Bristol-Myers Squibb Announces CheckMate -451 Study Did Not Meet Primary Endpoint of Overall Survival with Opdivo Plus Yervoy Vs. Placebo as A

Maintenance Therapy in Patients with Extensive-Stage Small Cell Lung Cancer After Completion of First-Line...

[xxix] De Ruysscher D, Pujol JL, Popat S, et al. STIMULI: a randomised open-label phase II trial of consolidation with nivolumab and ipilimumab in limited-stage SCLC after standard of care chemo-radiotherapy conducted by ETOP and IFCT. Ann Oncol. 2016;27(suppl_6):1430TiP-TiP.

[xxx] Nghiem PT, Bhathia S, Lipton EJ et al. PD-1 Blockade with pembrolizumab in advanced Merkel-Cell Carcinoma. N Engl J 2016 Jun 30; 374(26):2542-52.

[xxxi] Kaufman HL, Russell J, Hamid O et al. Avelumab in patients with chemotherapy-refractory metastatic Merkel cell carcinoma: a multicentre, single-group, open-label, phase 2 trial. Lancet Oncol. 2016 Oct;17 (10):1374-1385.

[xxxii] Antonia SJ, Bendell JC, Taylor MH, et al. Phase I/II study of nivolumab with or without ipilimumab for treatment of recurrent small cell lung cancer (SCLC): CA209-032. J Clin Oncol 33, 2015 (suppl; abstr 7503).

[xxxiii] Ott PA, Fernandez ME, Hiret S, et al. Pembrolizumab (MK-3475) in patients (pts) with extensive-stage small cell lung cancer (SCLC): Preliminary safety and efficacy results from KEYNOTE-028. J Clin Oncol 33, 2015 (suppl; abstr 7502).

[xxxiv] Topalian SL, Bhatia S, Hollebecque A et al. Abstract CT074: Non-comparative, open-label, multiple cohort, phase 1/2 study to evaluate nivolumab (NIVO) in patients with virus-associated tumors (CheckMate 358): Efficacy and safety in Merkel cell carcinoma (MCC). Cancer Research 2017; 77: CT074-CT074

[xxxv] Zhang P, Lu M, Li J et al. Efficacy and safety of PD-1 blockade with JS001 in patients with advanced neuroendocrine neoplasms: a non-randomized, open-label, phase 1b trial. Ann Oncol 2018, Volume 29, Issue suppl_8.

[xxxvi] Galluzzi L, Buque A, Kepp O, et al. Immunological effects of conventional chemotherapy and targeted anticancer agents. Cancer Cell 2015; 28: 690–714.

[xxxvii] Apetoh L, Ladoire S, Coukos G, et al. Combining immunotherapy and anticancer agents: the right path to achieve cancer cure? Ann Oncol 2015; 26: 1813–23.

[xxxviii] Rizvi NA, Hellmann MD, Brahmer JR, et al. Nivolumab in Combination With Platinum-Based Doublet Chemotherapy for First-Line Treatment of Advanced Non-Small-Cell Lung Cancer. J Clin Oncol. 2016 Sep 1;34 (25):2969-79.

[xxxix] Gadgeel SM, Stevenson J, Langer CJ, et al. Pembrolizumab (pembro) plus chemotherpay as front-line therapy for advanced NSCLC: KEYNOTE-021 cohorts A-C. Proc Am Soc Clin Oncol 2016; 34: Abstr 9016

[xl] Langer CJ, Gadgeel SM, Borghaei H, et al. Carboplatin and pemetrexed with or without pembrolizumab for advanced, non-squamous non-small-cell lung cancer: a randomised, phase 2 cohort of the open-label KEYNOTE-021 study. Lancet Oncol. 2016 Nov;17(11):1497-1508.

[xli] Camidge R, Liu S V, et al. Atezolizumab (MPDL3280A) Combined with Platinum-Based Chemotherapy in Non-Small Cell Lung Cancer (NSCLC): A Phase Ib Safety and

Efficacy Update. Journal of Thoracic Oncology • Volume 10, Number 9, Supplement 2, page S176, September 2015.

[xlii] Liu SV, Camidge DR, Gettinger SR et al.Long term follow-up of atezolizumab in combination with platinum-based doublet chemotherapy in patients with advanced non-small-cell lung cancer. Eur J Cancer 2018 2018 Sep;101:114-122.

[xliii] Common Terminology Criteria for Adverse Events (CTCAE) versión 5.0 Published: May 28, 2009 (v4.02: Sept. 15, 2009). http://evs.nci.nih.gov/ftp1/CTCAE/CTCAE_4.03_2010-06-14_QuickReference_8.5x11.pdf

[xliv] Eisenhauer E, Therasse P, Bogaerts J et al. New Response Evaluation Criteria in Solid Tumours: Revised RECIST Guideline (Version 1.1). Eur J Cancer. 2009 Jan; 45(2):228-47.

[xlv] Dancey JE, Dodd LE, Ford R, et al. Recommendations for the assessment of progression in randomised cancer treatment trials. Eur J Cancer 2009 Jan;45(2):281–9.

[xlvi] Wolchok JD, Hoos A, O´Day S et al. Guidelines for the evaluation of immune therapy activity in solid tumors: immune response criteria. Clin Cancer Res 2009 De 1; 15 (23): 7412-20.

[xlvii] Garcia-Carbonero R , Capdevila J, Crespo-Herrero G et al. Incidence, patterns of care and prognostic factors for outcome of gastroenteropancreatic neuroendocrine tumors (GEP-NETs): results from the National Cancer Registry of Spain (RGETNE). Ann Oncol. 2010 Sep;21(9):1794-803.

[xlviii] Sorbye H, Welin S, Langer SW et al. Predictive and prognostic factors for treatment and survival in 305 patients with advanced gastrointestinal neuroendocrine carcinoma (WHO G3): the NORDIC NEC study. Ann Oncol, 2013. 24(1): p. 152-60.

A phase II study of Platinum-doublet chemotherapy in combination with nivolumab as first-line treatment, in subjects with unresectable, locally advanced or metastatic G3 Neuroendocrine Neoplasms (NENs) of the gastroenteropancreatic (GEP) tract or of unknown (UK) origin.

Protocol identification number / code / Registration n°: CA209-73D

Sponsor code: GETNE-T1913

EudraCT number: 2019-001546-18

Acronym: NICE-NEC

Study Coordinator or Chair:

Rocío García-Carbonero, MD, PhD

Maria del Carmen Riesco-Martínez, MD, PhD

[redacted]

19/08/2019	**Protocol approval**
(date)	**Amendment 3**

Version: (4.1) / 15 November, 2021

Sponsor Signature Page

Protocol Number: GETNE-T1913

Study Title: A phase II study of Platinum-doublet chemotherapy in combination with nivolumab as first-line treatment, in subjects with unresectable, locally advanced or metastatic G3 Neuroendocrine Neoplasms (NENs) of the gastroenteropancreatic (GEP) tract or of unknown (UK) origin

EudraCT Number: 2019-001546-18

Version number and date: Protocol version 4.1, November 15th 2021

I have received and read the Investigator's Brochure for Nivolumab. I have read the **GETNE-T1913 - NICE-NEC STUDY** protocol and agree to conduct the study as outlined. I agree to maintain the confidentiality of all information received or developed in connection with this protocol and I agree to conduct this trial in accordance with all provisions of the protocol, GCPs and the Declaration of Helsinki.

Dr. Jaume Capdevila

Sponsor signature **Signature date (DD-MM-YYYY)**

Dr. Rocío García-Carbonero

Coordinating Investigator signature **Signature date (DD-MM-YYYY)**

Dr. Carmen Riesco Martínez

Coordinating Investigator signature **Signature date (DD-MM-YYYY)**

Investigator’s Agreement

Protocol Number: GETNE-T1913

Study Title: A phase II study of Platinum-doublet chemotherapy in combination with nivolumab as first-line treatment, in subjects with unresectable, locally advanced or metastatic G3 Neuroendocrine Neoplasms (NENs) of the gastroenteropancreatic (GEP) tract or of unknown (UK) origin

EudraCT Number: 2019-001546-18

Version number and date: Protocol version 4.1, November 15th 2021

I have received and read the Investigator’s Brochure for Nivolumab. I have read the **GETNE-T1913 - NICE-NEC STUDY** protocol and agree to conduct the study as outlined. I agree to maintain the confidentiality of all information received or developed in connection with this protocol and I agree to conduct this trial in accordance with all provisions of the protocol, GCPs and the Declaration of Helsinki.

____

Investigator Printed Name

____

Investigator Signature

____

Date:

SYNOPSIS
Clinical Protocol CA209- 73D

Sponsor code: GETNE-T1913
EudraCT number: 2019-001546-18

Protocol Title: A phase II study of platinum-doublet chemotherapy in combination with nivolumab as first-line treatment in subjects with unresectable, locally advanced or metastatic G3 neuroendocrine neoplasms (NENs) of the gastroenteropancreatic (GEP) tract or of unknown (UK) origin.

Sponsor: Grupo Español de Tumores Neuroendocrinos y Endocrinos (GETNE)

Investigational Product(s), Dose and Mode of Administration, Duration of Treatment with Investigational Product(s):

INDUCTION PHASE

- Nivolumab 360 mg IV will be administered every 3 weeks (±3 days) for 6 cycles
- Carboplatin (AUC=5) IV will be administered every 3 weeks (±3 days) for 6 cycles following the administration of nivolumab.
- Etoposide 100 mg/m^2/day IV will be administered on D1-3 every 3 weeks (±3 days) for 6 cycles following the administration of carboplatin.

MAINTENANCE PHASE

- Nivolumab 480 mg IV will be administered every 4 weeks (±3 days) for 2 years

Study Phase: Non-randomized phase II

Study Population:

- Patients with histologically confirmed non-resectable or stage IV G3 (Ki-67>20%) neuroendocrine neoplasms of the GEP tract or of UK origin.
- No prior systemic therapy.
- Available tumor sample for translational research.

Research Hypothesis:

- The addition of Nivolumab to platinum-based chemotherapy doublet will induce deeper and more durable tumor responses and prolong overall survival (OS) of patients with advanced G3 NENs of gastroenteropancreatic (GEP) or unknown (UK) origin, as compared to historical cohorts of patients treated with standard chemotherapy alone.

Objectives:

Objectives	Endpoints
**Primary**<br>● To determine the overall survival patients with advanced G3 NENs treated with nivolumab + platinum-based chemotherapy.	● 1 year-OS rate
**Secondary**<br>● To determine other efficacy outcomes of nivolumab + platinum-based chemotherapy in patients with advanced G3 NENs.	● ORR<br>● Duration of response<br>● PFS<br>● OS
● To evaluate the safety and tolerability of nivolumab + platinum-based chemotherapy in this patient population.	● Incidence of AEs, SAEs and selected AEs
● To evaluate biochemical response as predictive biomarker of efficacy of nivolumab + chemotherapy in this patient population.	● Chromogranin A and enolase values and their association with ORR, PFS and OS.
● To explore potential predictive and prognostic biomarkers.	● Mutational burden, gene expression signature, soluble factors and other molecular markers in peripheral blood and their association with clinical outcomes.

Study Design:

Protocol **CA209- 73D** is a non-randomized, open-label, phase 2 trial that will enroll 38 ≥ 18 years old with untreated metastatic or unresectable G3 NENs of gastroenteropancreatic or UK origin, evaluating the efficacy of nivolumab combined with platinum-based doublet chemotherapy as first-line treatment, followed by maintenance treatment with nivolumab in patients who have not progressed after 6 months of first line platinum-based induction chemotherapy.

Tumor progression and response endpoints will be assessed using RECIST 1.1 criteria. If there is no evidence of disease progression after the induction phase, treatment with nivolumab will continue until RECIST 1.1 defined progression or death, unacceptable toxicity, 24 months of treatment or withdrawal of consent.

Dose reductions will not be allowed for nivolumab. Treatment beyond initial investigator-assessed progression (either clinical or radiological) is permitted for nivolumab if the subject has an investigator-assessed clinical benefit and is tolerating well study drug.

The study period will include 18 months of accrual plus 24 months of follow-up after the induction phase. The study will end once survival follow-up has concluded.

Study Treatment

Medication	Potency	IP/Non-IP
Nivolumab	100 mg/vial	IP
Carboplatin	450 mg/vial	IP
Etoposide	100 mg/vial	IP

Figure-1. Study Schema

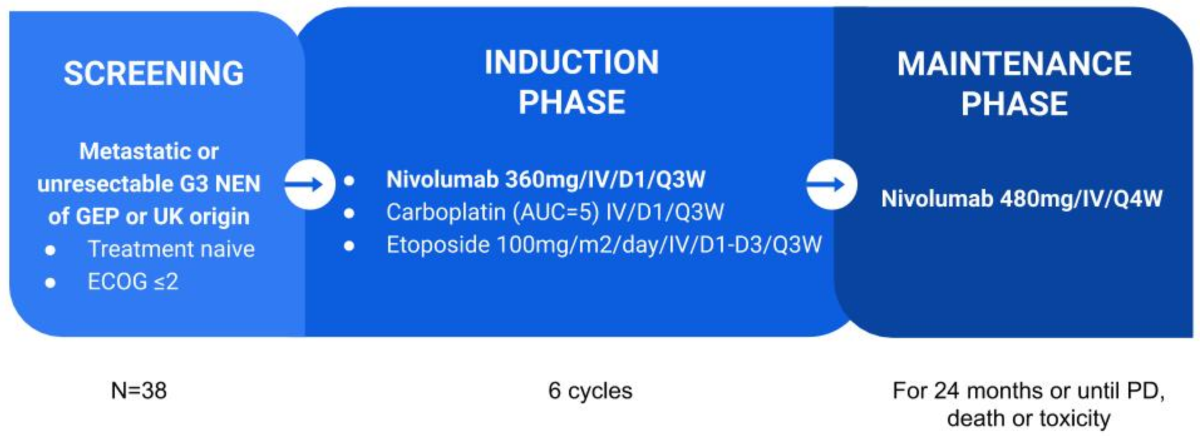

Study Population:
For study entry all the following criteria MUST be met:

Key Inclusion Criteria (See Protocol Section 4.1 for full list of criteria)

- Histologically confirmed G3 NENs originated in the gastroenteropancreatic tract (WHO 2015/2019 classification). Patients with a G3 NEN of unknown primary will also be eligible for this trial.
- Ki-67 >20% or mitotic rate > 20 per 10 HPF.
- Metastatic or locally advanced unresectable disease not amenable to treatment with curative intent.
- No prior systemic treatment for advanced disease nor as adjuvant therapy permitted.
- Availability of fresh or archive formalin-fixed, paraffin-embedded tumor tissue for biomarker assessment.
- Patients must have clinically and/or radiographically documented measurable disease.
- Adequate organ function as defined by the following criteria
  - Absolute neutrophil count (ANC) ≥1500 cells/mm3;
  - Platelets ≥100,000 cells/mm3;
  - Hemoglobin ≥9.0 g/dL;

- AST and ALT ≤2.5 x upper limit of normal (ULN); in patients with liver metastases AST and ALT ≤5.0 x ULN;
  - Total bilirubin ≤1.5 x ULN;
  - Serum creatinine ≤1.5 x ULN or calculated creatinine clearance ≥60 mL/min.
- ECOG performance status of 0-2.

Key Exclusion Criteria (See Protocol Section 4.2 for full list of criteria)

- The following endocrine tumor types may not be included: paraganglioma, adrenal, thyroid parathyroid or pituitary endocrine tumors. Large or small cell lung neuroendocrine carcinoma of the lung will also be excluded.
- Prior therapy with any immune checkpoint inhibitor.
- Prior organ transplantation, including allogeneic stem-cell transplantation.
- Systemic chronic steroid therapy (≥ 10 mg/day prednisone or equivalent) or other immunosuppressive agents or use of any investigational drug within 28 days before the start of trial treatment.
- Known history of positive testing for Human Immunodeficiency Virus (HIV) infection, known history of positive tests for Hepatitis B virus surface antigen (HBVsAg) or Hepatitis C ribonucleic acid (HCV RNA) indicating acute or chronic infection or other significant acute or chronic infections requiring medication at study entry.

Study Assessments:

The primary objective is to determine one-year OS rate of nivolumab in combination with platinum-doublet chemotherapy in treatment-naïve patients with unresectable G3 NENs. This will be calculated from the date of treatment initiation with platinum-based chemotherapy and nivolumab until the date of death from any cause. Subjects will be assessed for response every 8 weeks (± 7 days) after the date of administration of the first dose for 12 months and every 12 weeks (± 7 days) thereafter until disease progression. Patients that stop therapy due to toxicity in the absence of disease progression shall continue to be assessed with CT scans with the same frequency until disease progression or initiation of a new line of therapy. Upon treatment discontinuation, subjects will be followed for survival every 3 months (via telephone contact allowed) until death. All patients included will be followed.

Statistical Considerations:

Sample Size

A sample size of 38 subjects will provide 80% power to test the null hypothesis (H_0) that one-year OS rate for advanced G3 NENs treated with nivolumab in combination with platinum-doublet chemotherapy is 0.5 against a two-sided alternative. This design yields a two-sided type I error rate of 5% when the true one-year OS rate is 0.72.

Study Calendar:

-	Study start (real)	11/10/20199
-	First patient First visit (real)	4/11/2019
-	End or recruitment (real)	26/1/2021
-	Last patient Last visit (estimated)	1Q 2023
-	End of study (estimation)	2Q 2023

Table of contents

1. Background and introduction **14**
1.1. Background Disease Information 14
1.2. Background Therapeutic Information 15
1.2.1. Nivolumab: mechanism of action 15
1.2.2. Preclinical experience with Nivolumab 15
1.2.3. Clinical experience with Nivolumab in SCLC 15
1.2.4. Immune checkpoint inhibitors in other high grade NENs 17
1.2.5. Chemotherapy in combination with immune checkpoint inhibitors 17
1.2.6. Potential for drug-drug interactions 19

2. Objectives and Endpoints of the trial **20**
2.1. Primary objective 20
2.2. Secondary Objectives 20

3. Study Design **22**
3.1. Overall Design 22
3.1.1. Data Monitoring Committee and Other External Committees 22
3.2. Number of participants 23
3.3. End of Study Definition 23
3.4. Study Calendar 23

4. Patient selection criteria **24**
4.1. Inclusion Criteria 24
4.2. Exclusion Criteria 24
4.3. Screening Failures 26
4.3.1. Retesting during Screening Period 26

5. Therapeutic Regimens, Expected Toxicity, Dose Modifications **27**
5.1. Treatments Administered 27
5.1.1. Dosing 28
5.1.1.1. Nivolumab 28
5.1.1.2 Chemotherapy dosing 29
5.1.1.3 Premedication 29
5.1.2. Patient Monitoring 30
5.1.3. Treatment of Nivolumab Infusion Reactions 30
5.2. Dose Adjustments 32
5.2.1. Dose modifications for Nivolumab 33
5.2.2. Dose modifications for Chemotherapy 36
5.2.2.1 Dose Reductions for Hematologic Adverse Events 37
5.2.2.2. Dose Reductions for Non-Hematologic Adverse Events 38
5.2.3. Dose Delay Criteria 38

5.2.3.1. Dose delay Criteria for Nivolumab 38
5.2.3.2. Dose delay Criteria for Chemotherapy 39
5.2.4. Criteria to Resume Dosing 39
5.2.4.1. Criteria to Resume Nivolumab Dosing 39
5.2.4.2. Criteria to Resume Treatment with Chemotherapy 40
5.3. Duration of Therapy 40
5.3.1. Treatment Beyond Disease Progression 40
5.4. Preparation/Handling/Storage/ Accountability 41
5.5. Concomitant therapy 42
5.5.1. Prohibited and/or Restricted Therapy 42
5.5.2. Permitted Therapy 42
5.5.3. Palliative Local Therapy 43
5.5.4. Imaging Restrictions and Precautions 43

6. Discontinuation Criteria 45
6.1. Discontinuation from Study Treatment 45
6.1.1. Nivolumab Dose Discontinuation 45
6.1.2. Chemotherapy Dose Discontinuation 46
6.2. Discontinuation from Study 47
6.2.1. Post Study Treatment Study Follow-up 48
6.3. Lost to Follow-up 48

7. Clinical evaluation, laboratory tests, follow-up 49
7.1. Before treatment start 49
7.1.1. Informed Consent 49
7.1.1.1. General Informed Consent 49
7.1.1.2. Consent and Collection of Specimens for Future Biomedical Research 49
7.1.2. Inclusion/Exclusion Criteria Assessment 50
7.1.3. Medical History 50
7.1.4. Prior and concomitant medications review 50
7.1.4.1. Prior Medications 50
7.1.4.2. Concomitant Medications 50
7.1.5. Assignment of Screening Number 50
7.1.6. Subject Identification Card 50
7.1.7. Submitting Tumor Sample 50
7.1.8. Tumor Imaging 51
7.1.8.1. Computed (CT) with IV contrast or Magnetic Resonance Imaging (MRI) of chest, abdomen and pelvis. 51
7.1.8.2. Brain CT with IV contrast or MRI scan 51
7.1.8.3. Whole body bone scan 51
7.1.9. Laboratory Assessments 51
7.1.10. 12-Lead Electrocardiogram (ECG) 52
7.2. During treatment 52

7.2.1. Adverse Event (AE) Monitoring 52
7.2.2. Physical Exam 52
7.2.3. Vital Signs 52
7.2.4. 12-Lead Electrocardiogram (ECG) 52
7.2.5. Eastern Cooperative Oncology Group (ECOG) Performance Status 53
7.2.6. Tumor Imaging and Assessment of Disease 53
7.2.7. Laboratory Assessments 53
7.2.8. Serum/Urine β-hCG 54
7.3. After the end of treatment (Follow-up) 54
7.4. Summary table 54

8. Evaluation criteria 57
8.1. Definitions 57
8.2. Evaluation of efficacy 57
8.2.1. Measurability of tumour lesions at baseline 57
8.2.1.1. Definitions 57
8.2.1.2. Methods of measurements 58
8.2.2. Tumor response evaluation 58
8.2.2.1. Response evaluation according RECIST 1.1 [xliv] 58
8.2.2.2. Response evaluation according irRECIST [xlvi] 61
8.2.2.3. Frequency of tumor re-evaluation 61
8.2.3. Reporting of tumor response 62
8.2.3.1. Response duration 62
8.2.3.2. Stable disease duration 62
8.2.3.3. Progression Free Survival 62
8.2.3.4. Overall Survival 62

9. Statistical considerations 63
9.1. Statistical design 63
9.1.1. Sample Size Determination 63
9.2. Populations for Analysis 63
9.3. Statistical Analysis 63
9.3.1. Efficacy Analysis 63
9.3.1.1. Methods for Primary Endpoint 64
9.3.1.2. Methods for Secondary Endpoints 64
9.3.2. Safety Analysis 65

10. Translational research 66

11. Patient registration procedure 67

12. Reporting adverse events 68
12.1. Definitions for adverse event reporting 68
12.2. Reporting procedure 68

13. Quality assurance **73**
13.1. Control of data consistency 73
13.2. On-site quality control (for multi-centre studies only when data are sent to a Data Centre or similar) 73
13.3. Audits (for multi-centre studies only when data are sent to Data Centre or similar) 73
13.4. Central review of pathology 74

14. Ethical considerations **75**
14.1. Patient protection 75
14.2. Subject identification 75
14.3. Informed consent 75

15. Publication policy **77**

APPENDICES **78**
Appendix A. Performance Status Criteria 78
Appendix B. References 79

Schema:

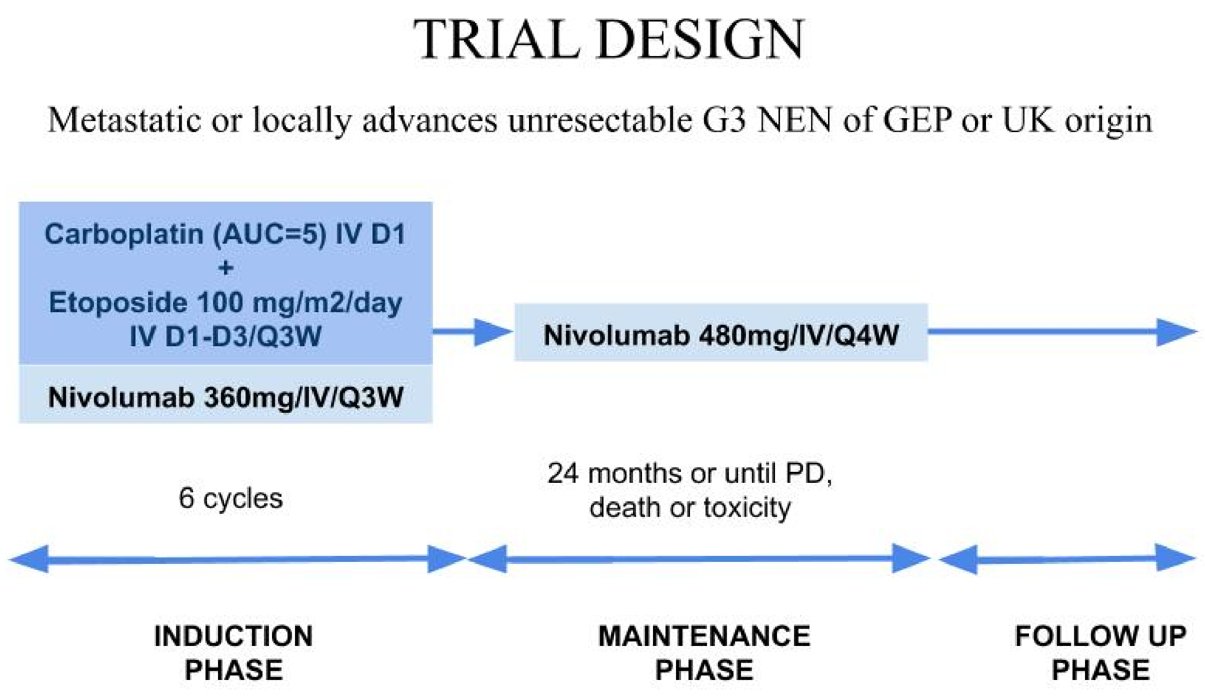
TRIAL DESIGN
Metastatic or locally advances unresectable G3 NEN of GEP or UK origin
Carboplatin (AUC=5) IV D1
+
Etoposide 100 mg/m2/day
IV D1-D3/Q3W
Nivolumab 360mg/IV/Q3W
Nivolumab 480mg/IV/Q4W
6 cycles
24 months or until PD,
death or toxicity
INDUCTION
PHASE
MAINTENANCE
PHASE
FOLLOW UP
PHASE

1. Background and introduction

1.1. Background Disease Information

NENs comprise a heterogeneous family of neoplasms with a wide and complex spectrum of clinical behaviour. They are uncommon tumours with an estimated incidence per year that ranges from 5 to 7 cases per 100,000 in the Caucasian population. The reported incidence has substantially increased over the last decades, al least partially due to improved diagnostic techniques and clinical awareness.[i],[ii]

G3 NENs are defined according to the World Health Organization (WHO) as those with high proliferative index (Ki-67 index greater than 20% or a mitotic rate greater than 20 per 10 high power fields (HPF)), which are generally, but not always, poorly differentiated tumors[iii].

The gastroenteropancreatic tract is the most common site for extra-pulmonary G3 NENs accounting for 35-55% of all NENs outside the lung. Approximately 50-60% of patients present with metastatic disease at the time of diagnosis and have a fateful prognosis. Median OS of patients with advanced disease is 5 months, ranging from less than a month for patients receiving best supportive care only, up to 11 months with best available therapy[iv],[v]. Historically, treatment strategies for extra-pulmonary G3 NENs have been often extrapolated from the treatment paradigm for small cell lung cancer (SCLC) given the histologic similarities between both entities. Therefore, standard front-line therapy involves the administration of a platinum agent (cisplatin or carboplatin) and etoposide, with response rates in the largest recent western series of ~ 30% and median survival of about one year [vi],[vii],[viii],[ix],[x]. The optimal duration of first line (1L) treatment is unclear. However, in the absence of progression, chemotherapy is usually stopped after 4 to 6 cycles due to cumulative toxicity and the lack of evidence demonstrating greater clinical benefit with extended treatment. Progression-free survival after this induction period remains poor, lasting 2- 3 months. Therefore, new therapeutic strategies are needed in this setting to improve response rates and prolong progression free survival without increasing toxicity in these patients. The addition of an immune checkpoint inhibitor to 1L chemotherapy, followed by maintenance immunotherapy could be a valuable option in this scenario.

More recently, a better understanding of the role of the immunological system in tumor control has opened multiple doors to implement different strategies to enhance immune response against cancer cells [xi]. It is well known that tumor cells elude immune response by several mechanisms. The programmed death-1 receptor (PD-1) and its ligand (PD-L1) are key therapeutic targets in the reactivation of the immune response against multiple cancers. PD-1 receptor is expressed on activated T cells and interacts with its ligand (PD-L1), which is expressed in tumor and immune cells, to down-regulate T-cell activation and promote *tumor immune escape*, a mechanism by which tumor cells avoid recognition and elimination by the immune system [xii]. The development of monoclonal antibodies against the PD-1/ PD-L1 pathway, that disrupt PD-1–mediated signalling and restore thereby antitumor immunity, has led to significant antitumor activity in a wide spectrum of neoplastic diseases, inducing increased progression-free-survival (PFS), overall survival (OS) and long lasting responses in different tumor types such as melanoma or lung cancer [xiii],[xiv],[xv],[xvi].

On the other hand, an increasing body of evidence has demonstrated that mutational burden is strongly associated with increased response to immunotherapy. In this regard, high grade NENs are suitable candidates for these treatment strategies as they are highly mutated tumours with high PD-L1 expression. For example, some authors have shown that virus-negative Merkel Cell Carcinomas

(MCCs), a type of high grade NEN, harbour more tumor neoantigens than melanomas or non-small cell lung cancers (median of 173, 65, and 111 neoantigens/sample, respectively), two cancers for which immune checkpoint blockade can produce durable clinical responses [xvii].

In summary, the addition of a checkpoint inhibitor could be a valuable treatment option in these patients. The objective of this trial is to determine the one-year overall survival rate of nivolumab in addition to platinum-doublet chemotherapy in advanced G3 NENs without prior therapy.

1.2. Background Therapeutic Information

1.2.1. Nivolumab: mechanism of action

Nivolumab (BMS-936558) is a fully human, IgG4 mAb that binds PD-1 on activated immune cells and disrupts engagement of the receptor with its ligands PD-L1 and PD-L2, abrogating inhibitory signals and augmenting the host antitumor response. The PD-1 receptor-ligand interaction is a major pathway hijacked by tumors to suppress immune control. The normal function of PD-1, expressed on the cell surface of activated T cells under physiological conditions, is to down-modulate unwanted or excessive immune responses, including autoimmune reactions. PD-1 has been shown to negatively regulate antigen receptor signaling upon engagement of its ligands (PD-L1 and/or PD L2)[xviii]. These ligands are constitutively expressed or can be induced in a variety of cell types, including various tumors [xix],[xx],[xxi]. Binding of PD-1 to its ligands inhibits T cell activation triggered through the T-cell receptor. PD-L2 is thought to control immune T-cell activation in lymphoid organs, whereas PD-L1 serves to dampen unwarranted T-cell function in peripheral tissues. A variety of cancers have been shown to express abundant levels of this PD-L1. This suggests that the PD-1/PD-L1 pathway plays a critical role in tumor immune evasion and can be an attractive target for therapeutic intervention.

1.2.2. Preclinical experience with Nivolumab

In vitro, nivolumab (BMS-936558) binds to PD-1 with high affinity (EC50 0.39 - 2.62 nM), and inhibits the binding of PD-1 to its ligands PD-L1 and PD-L2 (IC50: 1 nM). Nivolumab binds specifically to PD-1 and not to related members of the CD28 family such as CD28, ICOS, CTLA-4, and BTLA. Blockade of the PD-1 pathway by nivolumab results in a reproducible enhancement of both proliferation and IFN-γ release in the mixed lymphocyte reaction (MLR). Using a CMV re-stimulation assay with human PBMC, the effect of nivolumab on antigen specific recall response indicates that nivolumab augmented IFN-γ secretion from CMV specific memory T cells in a dose-dependent manner versus isotype-matched control. In vivo blockade of PD-1 by a murine analog of nivolumab enhances the anti-tumor immune response and result in tumor rejection in several immunocompetent mouse tumor models (MC38, SA1/N, and PAN02)[xxii].

1.2.3. Clinical experience with Nivolumab in SCLC

In early clinical trials, nivolumab has demonstrated activity in several tumor types, including melanoma, renal cell cancer (RCC), and NSCLC [xxiii].

Nivolumab is now approved in the U.S. and Europe to treat different tumor types including: melanoma (both adjuvant and metastatic setting), metastatic NSCLC in patients with progression on or after platinum-based chemotherapy, advanced SCLC with progression after platinum-based chemotherapy, advanced renal cell carcinoma, metastatic urothelial cancer and advanced squamous cell carcinoma of head and neck [xxiv].

The approval in SCLC was based on the results of Checkmate-032 [xxv], This was a phase I/II multicentre, multi-arm, open-label trial that included a cohort of 216 patients with SCLC. Patients progressing after at least one platinum-containing therapy were allocated to three treatment arms: nivolumab plus ipilimumab [1 mg/kg + 1 mg/kg iv (n = 3), 1 mg/kg + 3 mg/kg iv (n = 61), and 3 mg/kg + 1 mg/kg iv (n = 54)] versus nivolumab monotherapy (3 mg/kg iv) (n = 98). Nivolumab plus ipilimumab was administered every 3 weeks for four cycles followed by nivolumab 3 mg/kg iv every other week. The primary endpoint was objective response per RECIST v1.1. Objective response was achieved in 14/61 (23%) receiving nivolumab 1 mg/kg plus ipilimumab 3 mg/kg; 10/54 (19%) receiving nivolumab 3 mg/kg plus ipilimumab 1 mg/kg, 1/3 (33%) receiving nivolumab 1 mg/kg plus ipilimumab 1 mg/kg, and 10/98 (10%) patients in the nivolumab monotherapy arm. The median duration of response was 17.9 months for nivolumab monotherapy, and 14.2 months with nivolumab 1 mg/kg plus ipilimumab 3 mg/kg. Patients with ongoing responses at 2 years were 45% for nivolumab monotherapy and 36% in the combination. Responses were observed regardless of platinum sensitivity, line of therapy, or PD-L1 status. Two-year OS rates were 14% for nivolumab monotherapy and 26% in the combination arm, with a median OS of 4.1 (95% CI 3.0–6.8) and 7.8 (95% CI 3.6–14.2), respectively. In a randomized, phase II cohort from CheckMate 032 to further evaluate nivolumab ± ipilimumab, the initial efficacy of 242 patients was consistent with that in the non-randomized cohort [xxvi]. Grade ≥ 3 toxicities occurred in 18/61 (30%) in the nivolumab 1 mg/kg plus ipilimumab 3 mg/kg group, 10/54 (19%) in the nivolumab 3 mg/kg plus ipilimumab 1 mg/kg, and 13/98 (13%) in the nivolumab monotherapy. Six (6%) patients in the nivolumab 3 mg/kg group, seven (11%) in the nivolumab 1 mg/kg plus ipilimumab 3 mg/kg group, and four (7%) in the nivolumab 3 mg/kg plus ipilimumab 1 mg/kg group discontinued treatment due to treatments related adverse events (TRAEs). Four patients who received nivolumab plus ipilimumab died from TRAEs (myasthenia gravis, pneumonitis, encephalitis and hepatitis), and one patient who received nivolumab monotherapy died from treatment-related pneumonitis.

Several trials are currently evaluating nivolumab ± ipilimumab. Initial results from the randomized phase III clinical trial Checkmate 331(NCT02481830) evaluating the role of nivolumab versus chemotherapy (topotecan or amrubicin) in patients with relapsed SCLC have been recently presented showing no statistically significant improvement in OS with nivolumab vs chemotherapy (HR, 0.86 [95% CI, 0.72–1.04]); however OS curves showed delayed separation after month 12. HR for OS with nivolumab vs chemotherapy in patients with platinum-resistant SCLC was 0.71 (95% CI, 0.54–0.94) [xxvii].

Checkmate 451 evaluated the role of nivolumab alone, nivolumab 1 mg/kg in combination with ipilimumab 3 mg/kg, or placebo as consolidation/maintenance therapy after completion of platinum-based first-line chemotherapy in patients with extended disease SCLC. The primary endpoint of OS has not been met [xxviii]. Results from a phase II trial of consolidation with nivolumab and ipilimumab in localized SCLC after chemo-radiotherapy are pending (STIMULI, NCT02046733)[xxix].

In general, nivolumab has been well tolerated to date, with a favorable safety profile relative to anticipated toxicities based on an immunostimulatory mechanism of action.

1.2.4. Immune checkpoint inhibitors in other high grade NENs

Further research of these therapeutic strategies in other types of high grade NENs, such as Merkel cell carcinomas (MCC), has also proven to be highly effective both in chemonaive and pretreated patients. In a multicenter phase 2 trial, patients with advanced MCC who had received no previous systemic therapy were assigned to receive pembrolizumab at a dose of 2 mg per kilogram every 3 weeks. The primary endpoint was the objective response rate (ORR). Twenty-six patients were treated with pembrolizumab, with an ORR of 56% (95% confidence interval [CI], 35 – 76%) and a rate of progression free survival at 6 months of 67% [xxx]. Avelumab, an anti-PD-L1 monoclonal antibody, also showed efficacy in MCC refractory to chemotherapy in a phase II trial that enrolled 88 patients with advanced disease. Responses were achieved in 32% of patients, 82% of which were maintained at 10 months of follow-up time. Grade 3 treatment-related events occurred in only 5% of patients with no grade 4 events described [xxxi]. These promising results represent a new therapeutic option for advanced MCC and open a new path for future clinical trials in G3 NENs. In SCLC, which shares many histopathological and biological features with extrapulmonary high grade NEN, Nivolumab has recently shown clinical activity in a phase I/II study of SCLC patients previously treated with platinum-based chemotherapy [xxxii]. An objective response rate of 18% was documented in 40 patients treated with nivolumab monotherapy. Pembrolizumab has also recently showed promising activity in a phase IB study of 20 patients who had received prior platinum-based combination chemotherapy [xxxiii]. Preliminary results reported objective responses in 35% of the treated patients. Nivolumab has also been tested in a cohort of 25 patients with advanced MCC within the CheckMate 358 trial (NCT02488759)[xxxiv]. Preliminary results have reported an overall response rate of 68% in 22 evaluable patients. Responses occurred in both treatment-naïve (71%) and -experienced (63%) patients, regardless of tumor MCPyV or PD-L1 expression. Expansion cohorts will evaluate combinations of nivolumab with ipilimumab or anti-LAG-3.

More recently, initial results from an open-label, phase 1b trial evaluating the efficacy and safety of JS001 on advanced NENs with high proliferative index (Ki67>10%) who had failed to standard therapy, have been presented. JS001 is a monoclonal humanized IgG4 PD-1 antibody with a hinge S228P mutation that completely blocks PD-1 interactions with PD-L1 and PD-L2. A total of 35 patients were enrolled, with a majority of NECs (n=28). An encouraging 18% overall response rate and 25% disease control rate was achieved in NECs, with a 50% response rate in patients with PD1-positive tumors, and the median OS had not been reached. JS001 was tolerable in patients with NENs with <3% of severe adverse events [xxxv]. Other checkpoint inhibitors have not shown significant activity as monotherapy in G3 heavily pretreated NEN (i.e. pembrolizumab, PDR001). Currently, ongoing trials are evaluating the role of these immunomodulating agents as maintenance treatment at earlier stages of disease, and in combination with different therapeutic strategies including chemotherapy.

1.2.5. Chemotherapy in combination with immune checkpoint inhibitors

There is increasing evidence suggesting that chemotherapy not only has antitumor activity due to cytotoxic effects, but also through immunological effects, including reducing T-regulatory cell activity and enhancing cross-presentation of tumour antigens [xxxvi],[xxxvii]. Chemotherapy has also been shown to induce PD-L1 expression on tumour cells. Therefore, combining immunotherapy and chemotherapy could synergistically improve the anticancer activity of anti-PD-1 and anti-PD-L1 monotherapy.

Results from PD-1 inhibitors in combination with platinum-based chemotherapy in first line phase Ib/II trials have shown promising results in NSCLC. A phase I multicohort study was conducted to explore the efficacy and safety of nivolumab in combination with standard chemotherapy in first-line NSCLC [xxxviii]. 56 patients were assigned by histology to receive nivolumab 10 mg/kg iv every 3 weeks plus gemcitabine and cisplatin (squamous) or pemetrexed plus cisplatin (non-squamous) or nivolumab 5 or 10 mg/kg plus carboplatin-paclitaxel (all histologies). Patients received the combination treatment for four cycles followed by nivolumab alone until progression or unacceptable toxicity. No dose-limiting toxicities (DLTs) occurred during the first 6 weeks of treatment. Consistent with previous reports for immune checkpoint inhibitors monotherapy, grade 3-4 treatment-related adverse events were reported in 45% of patients. ORR were 33%, 47%, 47% and 43% for nivolumab 10 mg/kg plus gemcitabine-cisplatin, nivolumab 10 mg/kg plus pemetrexed-cisplatin, nivolumab 10 mg/kg plus paclitaxel-carboplatin, and nivolumab 5 mg/kg plus paclitaxel-carboplatin respectively. Responses were achieved regardless of tumor programmed death ligand-1 expression. The safety profile of nivolumab was consistent with that expected for individual agents with no particular concerns.

Recently, the international, multicohort, phase 1/2 KEYNOTE-021 study assessed the safety and anti-tumour activity of pembrolizumab added to three different platinum-based chemotherapy doublets in patients with advanced NSCLC [xxxix]. Chemotherapy-naïve NSCLC pts were randomly assigned to pembrolizumab 2 or 10 mg/kg every 3 weeks plus carboplatin (AUC 6) + paclitaxel 200 mg/m2 (any histology) or carboplatin (AUC 6) + paclitaxel 200 mg/m2 + bevacizumab 15 mg/kg (non-squamous) or carboplatin (AUC 5) plus pemetrexed 500 mg/m2 (nonsquamous) for 4 cycles followed by maintenance treatment with pembrolizumab, pembrolizumab + bevacizumab or pembrolizumab plus pemetrexed respectively. Seventy-four pts were included in the study. All combinations showed promising anti-tumour activity irrespective of tumour PD-L1 expression, with manageable safety profiles. One DLT occurred in cycle 1 (grade 3 rash). Grade 3-4 treatment-related AEs occurred in 36%, 46% and 42% of pts in each cohort respectively. Most common AEs were AST elevation (n=3), anemia (n=2), neutropenia (n=2) and febrile neutropenia (n=2). Based on these results, 123 patients with advanced chemotherapy-naïve NSCLC were randomized (1:1) in an open-label phase II trial to four cycles of carboplatin (AUC 5) plus pemetrexed 500 mg/m2 iv every three weeks alone or in combination with pembrolizumab 200 mg iv, flat dose, every three weeks, followed by maintenance with pembrolizumab for 24 months and pemetrexed until progressive disease or unacceptable toxicity. Patients were stratified according to PD-L1 expression (<1% vs ≥ 1%). 55% of patients in the pembrolizumab plus chemotherapy group achieved an objective response compared with 29% in the chemotherapy alone group (p=0.0016). The incidence of grade 3 or worse treatment-related adverse events was similar between both arms occurring in 23 pts (39%) in the pembrolizumab plus chemotherapy arm and 16 (26%) in the chemotherapy alone arm. Most common grade 3 or worse treatment-related adverse events were anaemia (12% in the pembrolizumab plus chemotherapy group vs 15% in the chemotherapy alone group), decreased neutrophil count (5% vs 3%), thrombocytopenia (3% in both arms) and decreased lymphocyte count, neutropenia, and sepsis (3% vs 2% each). The incidence of adverse events based on a presumed immunological mechanism of action, was 22% in the pembrolizumab plus chemotherapy group and 11% in the chemotherapy group. The only events of grade 3 or worse were one infusion reaction (2% in the pembrolizumab plus chemotherapy group vs none in the chemotherapy alone group), grade 3 skin reaction (one [2%] in the pembrolizumab plus chemotherapy group vs one [2%] in the chemotherapy group, and grade 3 pneumonitis (one [2%] in the pembrolizumab plus chemotherapy group). Deaths attributed to study treatment occurred in one patient (1%) in the pembrolizumab plus chemotherapy group due to sepsis

and two patients (3%) in the chemotherapy alone group (pancytopenia and sepsis). This study showed that the combination of pembrolizumab and platinum chemotherapy could be an effective and tolerable first-line treatment option for patients with NSCLC [xl].

Furthermore, a recently published clinical trial assessing atezolizumab in combination with different platinum-doublets in patients with newly diagnosed metastatic NSCLC showed encouraging efficacy. Seventy-six NSCLC patients were enrolled and assigned to three different treatment arms: atezolizumab plus carboplatin and paclitaxel (Arm C), atezolizumab plus carboplatin and pemetrexed (Arm D) or atezolizumab plus carboplatin and nab-paclitaxel (Arm E). Common treatment-related grade 3-4 adverse events were neutropenia (36% Arm C, 36% Arm D, 42% Arm E) and anaemia (16% Arm C, 16% Arm D, 31% Arm E). Confirmed ORRs were 36% Arm C, 68% Arm D (one CR) and 46% Arm E (four CRs). Median PFS was 7.1 months, (95% CI: 4.2-8.3), 8.4 months (95% CI: 4.7-11) and 5.7 months (95% CI: 4.4-14.8), respectively. Median OS was 12.9 months (95% CI: 8.8-21.3), 18.9 months (95% CI: 9.9-27.4) and 17.0 months (95% CI: 12.7-not evaluable), respectively. Atezolizumab was well tolerated in combination with all chemotherapy regimens tested[xli],[xlii].

Currently several phase Ib/II trials evaluating the combination of immune checkpoint inhibitors plus chemotherapy are ongoing.

1.2.6. Potential for drug-drug interactions

Given nivolumab is a therapeutic monoclonal antibody, it is not anticipated to be directly eliminated through hepatic/renal metabolism to compete with the elimination of platinum-doublet chemotherapy agents, which are mainly eliminated through metabolism and renal excretion.

As nivolumab is not considered a cytokine modulator, it is unlikely to have an effect on drug metabolizing enzymes or transporters in terms of inhibition or induction such as cytochrome P450 (CYP) enzymes. Therefore the risk of drug-drug interactions between nivolumab and platinum-doublet chemotherapy agents is anticipated to be low.

2. Objectives and Endpoints of the trial

The objectives and endpoints of the trial are summarized in Table 2-1.

2.1. Primary objective

- To determine the efficacy of nivolumab in combination with platinum-chemotherapy in patients with high grade neuroendocrine neoplasms, evaluated as one-year overall survival (OS) rate calculated from the date of treatment initiation until the date of death from any cause.

2.2. Secondary Objectives

- To determine overall response rate (ORR) as per Response Evaluation Criteria in Solid Tumors version 1.1 (RECIST v1.1) and the immune-related RECIST (irRECIST).
- To determine progression free survival (PFS) calculated from the date of treatment initiation with first-line chemotherapy and nivolumab until the date of first documentation of progressive disease as per RECIST v1.1 or death.
- To determine the OS calculated from the date of treatment initiation with nivolumab and platinum-based chemotherapy until the date of death from any cause.
- To assess biochemical response in patients with baseline elevation of chromogranin A and/or enolase, and correlate it with clinical outcome.
- To assess the safety and toxicity profile of nivolumab in combination with platinum-doublets in this patient population according to the Common Terminology Criteria for Adverse Events v.5.0 (CTCAE).
- To explore potential predictive or prognostic blood or tissue biomarkers.

Objectives	Endpoints
**Primary**<br>● To determine the overall survival patients with advanced G3 NENs treated with nivolumab + platinum-based chemotherapy.	● 1 year-OS rate
**Secondary**<br>● To determine other efficacy outcomes of nivolumab + platinum-based chemotherapy in patients with advanced G3 NENs.	● ORR<br>● Duration of response<br>● PFS<br>● OS
● To evaluate the safety and tolerability of nivolumab + platinum-based chemotherapy in this patient population.	● Incidence of AEs, SAEs and selected AEs
● To evaluate biochemical response as predictive biomarker of efficacy of nivolumab + chemotherapy in this patient population..	● Chromogranin A and enolase values and their association with ORR, PFS and OS.
● To explore potential predictive and prognostic biomarkers.	● Mutational burden, gene expression signature, soluble factors and other molecular markers in peripheral blood and their association with clinical outcomes.

3. Study Design

3.1. Overall Design

This is a multi-center phase II non-randomized, open-label trial investigating platinum-doublet chemotherapy in combination with nivolumab in patients with advanced G3 NENs of GEP or UK origin followed by maintenance treatment with nivolumab for up to 2 years in patients who have achieved complete response (CR), partial response (PR) or stable disease (SD) after 6 months of first line platinum-based induction chemotherapy with nivolumab.

All participants will be treated as described below:

- 6 cycles of chemotherapy (carboplatin (AUC= 5) iv on day 1 and etoposide 100 mg/m2/day iv on days 1-3, administered every 3 weeks) in combination with nivolumab 360 mg iv q3w (flat dose).
- In the absence of disease progression, nivolumab treatment will be continued until disease progression, unacceptable toxicity, death, withdrawal of consent, or a maximum treatment duration of 2 years, whichever occurs first. Participants will be permitted to continue on nivolumab beyond initial RECIST 1.1 defined progression, as long as they meet the criteria described in *section 5.3.1*

The study design schematic is presented in Figure 3.1-1

Figure 3.1-1

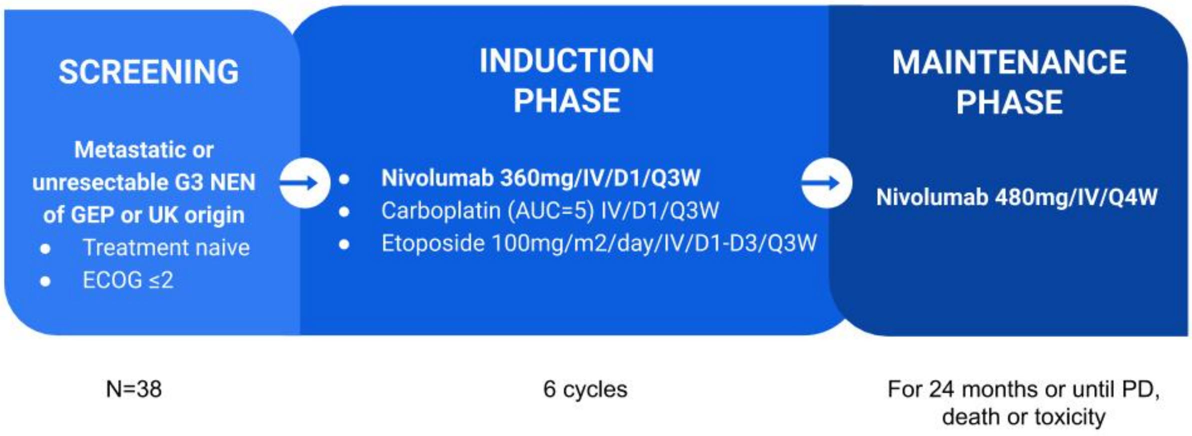

3.1.1. Data Monitoring Committee and Other External Committees

A Data Monitoring Committee (DMC) will not be required for this study, considering the following:

1. This study is an open-label study.
2. Data from the Checkmate-012 study has demonstrated a favourable and manageable safety profile that is consistent with prior experience with these agents.
3. Safety data will be closely monitored by BMS and the pharmacovigilance team of MFAR, with real time review and assessment of SAEs as they are received, and periodic review of all adverse events data for potential new safety signals.

3.2. Number of participants

The planned sample size is 38 patients with G3 NENs of gastroenteropancreatic or unknown primary origin.
Participating centres will include 10 academic hospitals selected from the GETNE Spanish network (Please see *Section 9*, statistical considerations, for specific details).

3.3. End of Study Definition

The duration of the study from start of enrollment to the last participant inclusion is expected to be 18 months. The study will end once additional survival follow-up of 24 months after the induction phase has concluded.

3.4. Study Calendar

- Study start (real)	11/10/2019
- First patient First visit (real)	4/11/2019
- End or recruitment (real)	26/1/2021
- Last patient Last visit (estimated)	1Q 2023
- End of study (estimation)	2Q 2023

4. Patient selection criteria

The following eligibility criteria are designed to select subjects for whom protocol treatment is considered appropriate. All relevant medical and non-medical conditions should be taken into consideration when deciding whether this protocol is suitable for a particular subject.

4.1. Inclusion Criteria

Patients should meet all of the following inclusion criteria:

1. Histologically confirmed G3 NENs originated in the gastroenteropancreatic tract (WHO 2010 classification). Patients with a G3 NEN of unknown primary will also be eligible for this trial.
2. Ki-67 >20% or mitotic rate > 20 per 10 HPF.
3. Metastatic or locally advanced unresectable disease not amenable to treatment with curative intent.
4. No prior systemic treatment for advanced disease nor as adjuvant therapy.
5. Availability of fresh or archive formalin-fixed, paraffin-embedded tumor tissue for biomarker assessment.
6. Patients must have clinically and/or radiographically documented measurable disease. At least one site of disease must be unidimensionally measurable as per RECIST 1.1.
7. Adequate organ function as defined by the following criteria (within 7 days prior to enrollment):
    a. absolute neutrophil count (ANC) ≥1500 cells/mm3
    b. platelets ≥100,000 cells/mm3
    c. hemoglobin ≥9.0 g/dL
    d. AST and ALT ≤2.5 x upper limit of normal (ULN); in patients with liver metastases AST and ALT ≤5.0 x ULN
    e. total bilirubin ≤1.5 x ULN
    f. serum creatinine ≤1.5 x ULN or calculated creatinine clearance ≥60 mL/min.
8. Male or female, age ≥18 years.
9. ECOG performance status of 0-2.
10. Life expectancy of ≥12 weeks.
11. Women of childbearing potential must have a negative serum or urine pregnancy test within 7 days prior to treatment initiation.
12. Highly effective contraception (i.e. methods with a failure rate of less than 1 % per year) for both fertile, sexually active male and female subjects. Highly effective contraception must be used 28 days prior to first trial treatment administration, for the duration of trial treatment, and at least for 5 months after the last dose of nivolumab and 6 months after the last dose of carboplatin/etoposide.
13. Signed and dated informed consent document must be given according to ICH/GCP, and national/local regulations indicating that the patient (or legally acceptable representative) has been informed of all pertinent aspects of the trial prior to enrolment.

4.2. Exclusion Criteria

Subjects shall not meet any of the following exclusion criteria:

1. The following endocrine tumor types may not be included: paraganglioma, adrenal, thyroid parathyroid or pituitary endocrine tumors. Large or small cell lung neuroendocrine carcinoma of the lung will also be excluded.
2. Prior therapy with any immune checkpoint inhibitor.
3. Major surgery, except diagnostic biopsy, in <4 weeks or radiation therapy <2 weeks prior to starting study treatment. Prior palliative radiotherapy to metastatic lesion(s) is permitted, provided there is at least one measurable lesion that has not been irradiated.
4. Prior organ transplantation, including allogeneic stem-cell transplantation.
5. Prior history of non-infectious pneumonitis requiring steroids or current pneumonitis.
6. Systemic chronic steroid therapy (> 10 mg/day prednisone or equivalent) or other immunosuppressive agents or use of any investigational drug within 28 days before the start of trial treatment. Short-term administration of steroids for allergic reactions or management of immune-related adverse events is allowed. Topical, inhaled, nasal and ophthalmic steroids are also allowed.
7. Use of any live vaccines against infectious diseases within 4 weeks of initiation of study treatment.
8. Known history of positive testing for Human Immunodeficiency Virus (HIV) infection, known history of or positive tests for Hepatitis B virus surface antigen (HBVsAg) or Hepatitis C ribonucleic acid (HCV RNA) indicating acute or chronic infection or other significant acute or chronic infections requiring medication at study entry.
9. Active, known or suspected autoimmune disease or a documented history of autoimmune disease, including ulcerative colitis and Crohn’s disease. (Patients with vitiligo, type I diabetes mellitus, residual hypothyroidism due to autoimmune condition only requiring hormone replacement, psoriasis not requiring systemic treatment, or conditions not expected to recur in the absence of an external trigger are permitted to enroll).
10. Active seizure disorder or evidence of brain metastases, spinal cord compression, or carcinomatous meningitis.
11. A serious uncontrolled medical disorder or active infection that would impair their ability to receive study treatment will not be allowed to enter the study. Any of the following within the 12 months prior to study drug administration: myocardial infarction, uncontrolled angina, coronary/peripheral artery bypass graft, NYHA class ≥ III congestive heart failure, cerebrovascular accident or transient ischemic attack and 6 months for deep vein thrombosis or pulmonary embolism.
12. Known hypersensitivity reactions to monoclonal antibodies (≥ grade 3 according to NCI Common Terminology Criteria for Adverse Events (CTCAE) v 5.0 [xliii] or any past medical history of anaphylaxis or uncontrolled asthma (i.e., 3 or more asthma characteristics partially controlled).
13. Any other prior malignancy within 5 years of study entry, with the exception of adequately treated in-situ carcinoma of the cervix, breast or uteri, or non-melanomatous skin cancer.
14. Any psychological, familial, sociological or geographical condition potentially hampering compliance with the study protocol and follow-up schedule; those conditions should be discussed with the patient before registration in the trial.
15. Dementia or significantly altered mental status that would prohibit the understanding or rendering of informed consent and compliance with the requirements of this protocol.
16. Female patients who are pregnant or lactating, or men and women of reproductive potential not willing or not able to employ an effective method of birth control/contraception to prevent

pregnancy during treatment and for 6 months after discontinuing study treatment The definition of effective contraception should be in agreement with local regulation and based on the judgment of the principal investigator or a designated associate.

17. Other severe acute or chronic medical or psychiatric condition, or laboratory abnormality that may increase the risk associated with study participation or study drug administration, or may interfere with the interpretation of study results, and in the judgment of the investigator would make the patient inappropriate for study entry.

4.3. Screening Failures

Screening failures are defined as participants who consent to participate in the clinical study but are not subsequently randomized. A minimal set of screen failure information is required to ensure transparent reporting of screen failure participants, to meet the Consolidated Standards of Reporting Trials (CONSORT) publishing requirements, and to respond to queries from regulatory authorities. Minimal information includes date of consent, demography, screen failure details, eligibility criteria, and any serious AEs.

4.3.1. Retesting during Screening Period

This study permits the re-enrollment of a participant that has discontinued the study as a pre-treatment failure (ie, participant has not been treated). If re-enrolled, the participant must be re-consented. Retesting of laboratory parameters and/or other assessments within any single screening will be permitted (in addition to any parameters that require a confirmatory value).

5. Therapeutic Regimens, Expected Toxicity, Dose Modifications

Study treatment is defined as any investigational treatment(s), marketed product(s), placebo or medical device intended to be administered to a study participant according to the study treatment allocation.
Study treatment includes both Investigational [Medicinal] Product (IP/IMP) and Non-investigational [Medicinal] Product (Non-IP/Non-IMP) and consist of the following:

- Nivolumab
- Carboplatin
- Etoposide

Other medications used as support or escape medication for preventative, diagnostic, or therapeutic reasons, as components of the standard of care for a given diagnosis, may be considered as non-investigational products.

Study Treatments for Study CA209- 73D

Product description and dosage Form	Potency	IP/Non-IP	Blinded or Open Label	Primary Packaging (Volume)/Label Type and Secondary Packaging (Qty)/Label Type	Appearance	Storage Conditions (per label)
BMS-936558-01 Nivolumab Solution for Injection[a]	100 mg/vial (10 mg/mL)	IP	Open Label	10 mL per vial and 5 or 10 vials per carton	Clear to opalescent colorless to pale yellow liquid. May contain particles	2 to 8 C. Protect from light and freezing
Carboplatin Solution for injection[b]	450 mg/vial (10 mg/mL)	IP	Open Label	Various packaging configuration	Clear, colorless or slightly yellow solution.	Product should be stored as per market product conditions.
Etoposide	100 mg/ vial (20mg/ mL)	IP	Open Label	Various packaging configuration	Powder, white to white with a yellow cast.	Product should be stored as per market product conditions.

NOTES: [a] May be labeled as either “BMS-936558-01” or “Nivolumab”

5.1. Treatments Administered

The treatments that will be used in this trial are outlined below (Table-5.1). They will be administered in the order presented.
All trial treatments may be administered on an outpatient basis.

Table-5.1. Trial treatments

Drug	Drug	Frequency	Administration	Treatment period	Use
Nivolumab	360 mg	Q3W	IV infusion	Day 1 of each cycle	Experimental
Carboplatin	AUC5	Q3W	IV infusion	Day 1 of each cycle	Standard
Etoposide	100 $mg/m^2/day$	Q3W	IV infusion	Days 1-3 of each cycle	Standard

Nivolumab will be administered first as a 30 minute IV infusion, followed by the carboplatin infusion and then the etoposide infusion. Platinum-doublet will start at least 30 minutes after completion of the nivolumab infusion. At the investigator's discretion, nivolumab may be administered over a longer infusion time (60 minutes) if the participant developed a prior infusion reaction.

Nivolumab treatment will continue until disease progression, discontinuation due to unacceptable toxicity, or withdrawal of consent. Participants will be treated up to 24 months in the absence of disease progression or unacceptable toxicity.

All participants will be monitored continuously for adverse events (AEs) while on study treatment. Treatment modifications (eg, dose delay, reduction, retreatment, or discontinuation) will be based on specific laboratory and adverse event criteria, as described in *sections 5.2-5.3.*

5.1.1. Dosing

5.1.1.1. Nivolumab

Participants will receive nivolumab, followed by chemotherapy on day 1 of every 3 weeks cycle for 6 cycles (*induction phase*). At the time of completion of 6 cycles of chemotherapy and nivolumab, participants who have not experienced disease progression will continue to receive nivolumab at a dose of 480 mg as 30 minute infusion every 4 weeks for up to 2 years (*maintenance phase*).

Drug	Dose	Frequency	Administration	Treatment period	Use
**Induction phase**					
Nivolumab	360 mg	Q3W	IV infusion	Day 1 of each cycle	Experimental
**Maintenance phase**					
Nivolumab	480 mg	Q4W	IV infusion	Day 1 of each cycle	Experimental

Treatment will continue until progression, unacceptable toxicity, withdrawal of consent, whichever occurs first. Treatment with nivolumab will be given for up to 24 months including the induction phase (a total of 34 cycles) in the absence of disease progression or unacceptable toxicity.

The assessment for discontinuation of nivolumab should be made separately from chemotherapy. If criteria for discontinuation for nivolumab are met, platinum-based doublet chemotherapy may continue until 6 cycles have been completed. If a participant meets criteria for discontinuation and investigator is unable to determine whether the event is related to all or one study drug, the participant should discontinue all study drugs and be taken off the study.

5.1.1.2 Chemotherapy dosing

6 cycles of carboplatin in combination with etoposide will be administered every 3 weeks (Q3W).

All chemotherapy agents' preparation, premedication, administration, monitoring, and management of complications are to follow local prescription guidelines and regulations. The dose of chemotherapy may be capped per local standards.

❑ Carboplatin:

Carboplatin (AUC=5) will be administered as a 15-60 minute IV infusion per site's standard practice Q3W on day 1 of each treatment cycle. Carboplatin will be administered to participants at least 30 minutes following the end of the nivolumab infusion.

The dose of carboplatin will be calculated using the *Calvert formula* as follows:

Carboplatin dose (mg)= AUC target x ([CrCl (ml/min) + 25]

Creatinine clearance (CrCl) calculation is based on the *Cockcroft-Gault formula* and should include the most recent serum creatinine and the most recent weight.

NOTE: If calculation of the CrCl by the Cockcroft-Gault formula yields a result of > 125 mL/min, then a CrCl should be calculated by an alternative formula per institutional standards or capped at 125 mL/min.

The dose of carboplatin may be capped per local standards.

❑ Etoposide:

Etoposide 100 mg/m^2/day will be administered as a 30-60 minute IV infusion per site's standard practice Q3W on days 1, 2 and 3 of each treatment cycle. Etoposide will be administered following the end of carboplatin infusion.

5.1.1.3 Premedication

❑ Nivolumab

Patients should not receive pre-medication to prevent infusion reaction before the first infusion of nivolumab. If a patient experiences an infusion reaction, he/she may receive premedication prior to subsequent dosing days (*Table 5.1.3).*

❑ Carboplatin and Etoposide:

Antiemetic premedication will be administered according to local standards. Recommended antiemetic treatments are:

- ❑ Dexamethasone (dosing according to local standards; an equivalent dose of another corticosteroid may be administered).

- ❑ A 5-HT3 receptor antagonist (type per investigator discretion and local standards-of-care).

Additional use of antiemetic premedications may be employed at the discretion of the Investigator. Please refer to the product label or local standards of care for carboplatin and etoposide supportive measures.

5.1.2. Patient Monitoring

Vital signs including temperature, pulse, respiratory rate, weight and blood pressure prior to the administration of each dose of trial treatment will be measured as specified in *section 7.4.*

5.1.3. Treatment of Nivolumab Infusion Reactions

Since nivolumab contains only human immunoglobulin protein sequences, it is unlikely to be immunogenic and induce infusion or hypersensitivity reactions. However, if such a reaction were to occur, it might manifest with fever, chills, rigors, headache, rash, pruritus, arthralgias, hypo- or hypertension, bronchospasm, or other symptoms. All Grade 3 or 4 infusion reactions should be reported within 24 hours to the BMS Medical Monitor and reported as a SAE if criteria are met. Infusion reactions should be graded according to NCI CTCAE (Version 5.0) guidelines.

Treatment recommendations are provided below and may be modified based on local treatment standards and guidelines, as appropriate:

For Grade 1 symptoms: (mild reaction; infusion interruption not indicated; intervention not indicated)

- Remain at bedside and monitor the patient until recovery from symptoms. The following prophylactic premedications are recommended for future infusions: diphenhydramine 50 mg (or equivalent) and/or acetaminophen/paracetamol 325 to 1000 mg at least 30 minutes before additional nivolumab administration.

For Grade 2 symptoms: (moderate reaction requires therapy or infusion interruption but responds promptly to symptomatic treatment [eg, antihistamines, non-steroidal anti-inflammatory drugs, narcotics, corticosteroids, bronchodilators, IV fluids]; prophylactic medications indicated for < 24 hours)

- Stop the nivolumab infusion, begin an IV infusion of normal saline, and treat the participant with diphenhydramine 50 mg IV (or equivalent) and/or acetaminophen/paracetamol 325 to 1000 mg; remain at bedside and monitor participant until resolution of symptoms. Corticosteroid and/or bronchodilator therapy may also be administered as appropriate. If the infusion is interrupted, then restart the infusion at 50% of the original infusion rate when symptoms resolve; if no further complications ensue after 30 minutes, the rate may be increased to 100% of the original infusion rate. Monitor participant closely. If symptoms recur, then no further nivolumab will be administered at that visit. Administer diphenhydramine 50 mg IV, and remain at bedside and monitor the participant until resolution of symptoms. The amount of study drug infused must be recorded on the electronic case report form (eCRF).
- For future infusions, the following prophylactic premedications are recommended: diphenhydramine 50 mg (or equivalent) and/or acetaminophen/paracetamol 325 to 1000 mg should be administered at least 30 minutes before nivolumab infusion. If necessary, corticosteroids (up to 25 mg of hydrocortisone or equivalent) may be used.

For Grade 3 or 4 symptoms: (severe reaction, Grade 3: prolonged [ie, not rapidly responsive to symptomatic medication and/or brief interruption of infusion]; recurrence of symptoms following initial improvement; hospitalization indicated for other clinical sequelae [eg, renal impairment, pulmonary infiltrates]. Grade 4: Life threatening; pressor or ventilator support indicated)

- Immediately discontinue infusion of nivolumab. Begin an IV infusion of normal saline and treat the participant as follows: Recommend bronchodilators, epinephrine 0.2 to 1mg of a 1:1000 solution for subcutaneous administration or 0.1 to 0.25 mg of a 1:10.000 solution injected slowly for IV administration, and/or diphenhydramine 50 mg IV with methylprednisolone 100 mg IV (or equivalent), as needed. Participant should be monitored until the investigator is comfortable that the symptoms will not recur. Nivolumab will be permanently discontinued. Investigators should follow their institutional guidelines for the treatment of anaphylaxis. Remain at bedside and monitor participant until recovery of the symptoms.

In case of late-occurring hypersensitivity symptoms (eg, appearance of a localized or generalized pruritus within 1 week after treatment), symptomatic treatment may be given (eg, oral antihistamine or corticosteroids).

Table 5.1.3 Infusion reaction guidelines

Grade	Treatment	Premedication at subsequent dosing
Grade 1	Increase monitoring of vital signs until the subject is deemed medically stable in the opinion of the investigator.	Subject may be premedicated at least 30 minutes prior to nivolumab infusion with:<br>● Diphenhydramine 50 mg po (or equivalent dose of antihistamine).<br>● Acetaminophen 325-1000 mg po.
Grade 2	**Stop Infusion and monitor symptoms.**<br>Treat the participant with:<br>● IV infusion of normal saline<br>● Diphenhydramine 50 mg IV<br>● Acetaminophen/ paracetamol 325-1000mg.<br>● Increase monitoring of vital signs as medically indicated until the subject is deemed medically stable in the opinion of the investigator.<br>● If symptoms resolve within one hour of stopping drug infusion, the infusion may be restarted at 50% of the original infusion rate (e.g., from 100 mL/hr to 50 mL/hr). Otherwise dosing will be held until symptoms resolve and the subject should be premedicated for the next scheduled dose.	Subject should be premedicated at least 30 minutes prior to nivolumab infusion with:<br>● Diphenhydramine 50 mg po (or equivalent dose of antihistamine).<br>● Acetaminophen 325-1000 mg po.<br>● If necessary corticosteroids may be used (up to 25 mg of hydrocortisone or equivalent).

Grade 3-4	**Stop Infusion.**<br>Treat the participant with:<br>● IV infusion of normal saline.<br>● Bronchodilators if required.<br>● Epinephrine 0.2 to 1 mg of a 1:1000 solution sc.<br>● Diphenhydramine 50 mg IV.<br>● Methylprednisolone 100 mg IV (or equivalent).<br>● Remain at bedside and monitor participant until recovery of the symptoms and stable in the opinion of the investigator.<br>● Hospitalization may be indicated.<br><br>**Nivolumab permanently discontinued**	**No subsequent dosing**
Appropriate resuscitation equipment should be available in the room and a physician readily available during the period of drug administration.		

5.2. Dose Adjustments

- The Investigator may attribute each toxicity event to carboplatin, etoposide or nivolumab alone and use a dose reduction according to *Tables 5.2, 5.1.3.* For individual subjects requiring a dose modification, treatment for each new cycle may be delayed if the scheduled off-drug periods are not adequate to allow for recovery to Grade ≤1 or the baseline status of the subject.
- Nivolumab dose reductions are not permitted. Nivolumab treatment may be interrupted or discontinued due to toxicity for up to 6 weeks (longer interruptions shall be discussed and approved by the medical monitor on an individual basis). If toxicity has not recovered to at least a grade 1 the patient shall be permanently discontinued from the study. Longer dose interruptions.
- If a dose reduction for toxicity occurs with any agent, the dose may not be re-escalated. Subjects can have a maximum of 2 dose modifications to each of the components of study therapy throughout the course of the study for toxicities. If a subject experiences several toxicities and there are conflicting recommendations, dose reduction will be done according to the most severe toxicity. Subjects who require a 3rd dose modification to any particular component will have that agent discontinued.
- Reduction of one chemotherapy agent and not the other agent is appropriate if, in the opinion of the investigator, the toxicity is clearly related to one of the treatments. If, in the opinion of the investigator, the toxicity is related to the combination of both chemotherapy agents, both drugs should be reduced according to recommended dose modifications. If the toxicity is related to the combination of three agents, chemotherapy should be reduced, interrupted or discontinued and nivolumab should be interrupted or discontinued according to the recommended dose modifications.

Table 5.2. Dose modifications for trial medications

Drug	Dose level 0	Dose level -1	Dose level -2	Dose level -3

Carboplatin	AUC 5	AUC 4	AUC 3	Discontinue
Etoposide	100 mg/m2	80 mg/m2	60 mg/m2	Discontinue
Nivolumab	360 mg/m2	Dose reductions not permitted		

5.2.1. Dose modifications for Nivolumab

There will be no dose reductions for nivolumab. Adverse events (AEs), both non-serious and serious, associated with nivolumab exposure may represent an immunologic etiology. These adverse events may occur shortly after the first dose or several months after the last dose of treatment. AEs can differ in severity and duration from AEs caused by other therapeutic classes. Early recognition and management of AEs associated with immune-oncology agents may mitigate severe toxicity. Nivolumab must be withheld for drug-related toxicities and severe or life-threatening AEs as per *Table 5.2.1* below.

Table 5.2.1 Nivolumab guidelines for drug-related adverse events.

**Toxicity**	**Grade of event**	**Management**	**Follow-up**
**Diarrhea/Colitis**	1	Continue immunotherapy per protocol.<br>Symptomatic treatment	-Close monitoring for worsening symptoms.<br>-Educate patient to report worsening immediately.<br>If worsens:<br>-Treat as Grade 2 or 3/4.
	2	Hold treatment until toxicity resolves to grade 0-1.<br>Symptomatic treatment.	If improves to grade 1:<br>-resume therapy per protocol<br>If persists> 5-7 days or recur:<br>-0.5-1.0 mg/kg/day methylprednisolone or equivalent.<br>-When symptoms improve to grade 1, taper steroids over at least 1 month, consider prophylactic antibiotic for opportunistic infections, and resume immunotherapy per protocol.<br>If worsens or persists>3-5 days with oral steroids:<br>-Treat as grade 3-4.

	3-4	-Discontinue treatment.<br>-1.0 to 2.0 mg/kg/day methylprednisolone IV or equivalent.<br>- Add prophylactic antibiotics for opportunistic infections.<br>-Consider lower endoscopy	<u>If improves:</u><br>-Continue steroids until grade 1, then taper over at least 1 month.<br><u>If persists >3-5 days or recurs after improvement:</u><br>-Add infliximab 5mg/kg (if no contraindication).<br>Note: infliximab should not be used in cases of perforation or sepsis.
**AST, ALT, Bilirubin**	1	Continue treatment per protocol	Continue LFT monitoring per protocol.<br><u>If worsens:</u><br>-Treat as Grade 2 or 3-4.
	2	-Hold treatment until toxicity resolves to grade 0-1<br>-Increase frequency of monitoring to every 3 days.	<u>If returns to baseline:</u><br>-Resume routine monitoring, resume therapy per protocol.<br><u>If elevations persist >5-7 days or worsen:</u><br>-0.5-1 mg/kg/day methylprednisolone or oral equivalent and when LFT returns to grade 1 or baseline, taper steroids over at least 1 month, consider prophylactic antibiotics for opportunistic infections and resumen therapy per protocol.
**AST, ALT, Bilirubin**	3-4	-Discontinue nivolumab [1]<br>-Increase frequency of monitoring to every 1-2 days.<br>-1.0 to 2.0 mg/kg/day methylprednisolone IV or IV equivalent [2]	<u>If returns to grade 2</u>:<br>-Taper steroids over at least 1 month.<br><u>If does not improve in >3-5 days, worsens or rebounds:</u><br>-Add mycophenolate mofetil 1 g BID.<br>-If no response within an additional 3-5 days, consider other immunosuppressants per local guidelines.
**Skin Rash**	1-2	-Symptomatic therapy (eg. antihistamines, topical steroids).<br>-Continue nivolumab per protocol.	<u>If persists >1-2 weeks or recurs:</u><br>-Consider skin biopsy.<br>-Delay nivolumab per protocol.<br>-Consider 0.5-1.0 mg/kg/day methylprednisolone IV or oral equivalent. Once improving, taper steroids over at least 1 month, consider prophylactic antibiotics for opportunistic infections, and resume nivolumab per protocol.<br><u>If worsens:</u><br>-Treat as Grade 3-4.
	3-4	-Hold or discontinue nivolumab per protocol.<br>-Consider skin biopsy.<br>-Dermatology consult.<br>-1.0 to 2.0 mg/kg/day methylprednisolone IV or IV equivalent.	If it improves to Grade 1:<br>-Taper steroids over at least 1 month and add prophylactic antibiotics for opportunistic infections.<br>- Resume nivolumab per protocol.

**Pneumonitis**	1	-Consider delay of nivolumab.<br>-Monitor for symptoms every 2-3 days.<br>-Consider Pulmonary and ID consults.	-Re-image at least every 3 weeks.<br>If worsens:<br>-Treat as Grade 2 or 3-4
	2	-Delay of nivolumab per protocol.<br>-Monitor for symptoms daily,consider hospitalization.<br>- Pulmonary and ID consults.<br>-1.0 mg/kg/day methylprednisolone IV or oral equivalent.<br>- Consider bronchoscopy, lung biopsy.	-Re-image every 1-3 days.<br>If improves:<br>-When symptoms return to near baseline, taper steroids over at least 1 month and then resume nivolumab per protocol and consider prophylactic antibiotics.<br>If not improving after 2 weeks or worsening:<br>-Treat as Grade 3-4.
**Pneumonitis**	3-4	-Discontinue nivolumab per protocol.<br>-Hospitalize.<br>-Pulmonary and ID consults.<br>-2-4 mg/kg/day methylprednisolone IV or equivalent.<br>-Add prophylactic antibiotics for opportunistic infections.<br>- Consider bronchoscopy, lung biopsy.	If improves to baseline:<br>-Taper steroids over at least 6 weeks.<br>If not improving after 48 hours or worsening:<br>-Add additional immunosuppression.
**Renal failure or nephritis**	1	-Continue nivolumab per protocol.<br>-Monitor creatinine weekly	If returns to baseline:<br>-Resume routine creatinine monitoring per protocol.<br>If worsens:<br>-Treat as Grade 2 or 3-4.
	2-3	-Hold nivolumab per protocol.<br>-Monitor creatinine every 2-3 days.<br>0.5-1 mg/kg/day methylprednisolone IV or equivalent.<br>-Consider renal biopsy with nephrology consult.	If returns to Grade 1:<br>-Taper steroids over at least 1 month, consider prophylactic antibiotics for opportunistic infections, and resume nivolumab and routine creatinine monitoring per protocol.<br><br>If elevations persist >7 days or worsen:<br>-Treat as Grade 4.
	4	-Discontinue nivolumab per protocol.<br>-Monitor creatinine daily.<br>-1.0-2.0mg/kg/day methylprednisolone IV or IV equivalent.<br>-Consider renal biopsy.<br>- Consult nephrologist.	If returns to Grade 1:<br>-Taper steroids over at least 1 month, consider prophylactic antibiotics for opportunistic infections.

	Management	Follow-up
**Asymptomatic TSH elevation**	-Continue nivolumab per protocol.<br>-If TSH<0.5 X LLN, or TSH>2 x ULN, or consistently out of range in 2 subsequent measurements :include fT4 at subsequent cycles as clinically indicated; consider endocrinology consult.	
**Symptomatic endocrinopathy**	-Evaluate endocrine function.<br>-Consider pituitary scan.<br><u>Symptomatic with abnormal lab/pituitary scan:</u><br>-Delay nivolumab per protocol.<br>-1-2 mg/kg/day methylprednisolone or PO equivalent.<br>-Initiate appropriate hormone therapy.<br><u>No abnormal lab/pituitary MRI scan but symptoms persist:</u><br>-Repeat labs in 1-3 weeks/MRI in 1 month.	If improves (with or without hormone replacement):<br>-Taper steroids over at least 1 month, consider prophylactic antibiotics for opportunistic infections.<br>-Resume nivolumab per protocol.<br>- Patients with adrenal insufficiency may need to continue steroids with mineralocorticoid component.
**Suspicion of adrenal crisis (e.g. severe dehydration, hypotension, shock out of proportion to current illness)**	-Delay or Discontinue nivolumab therapy per protocol.<br>-Rule out sepsis.<br>-Stress dose of IV steroids with mineralocorticoid activity.<br>-IV fluids.<br>-Consult endocrinologist.<br>-If adrenal crisis ruled out, then trat as above for symptomatic endocrinopathy.	
[1] Nivolumab may be delayed rather than discontinued if AST/ALT ≤ 8 X ULN or Total Bilirubin ≤ 5 x ULN.<br>[2] The recommended starting dose for grade 4 hepatitis is 2 mg/kg/day methylprednisolone IV.<br>[3] Patients with intolerable or persistent Grade 2 drug-related AE may hold study medication at physician discretion. Permanently discontinue study drug for persistent Grade 2 adverse reactions for which treatment with study drug has been held, that do not recover to Grade 0-1 within 12 weeks of the last dose.		

5.2.2. Dose modifications for Chemotherapy

Dose reductions of chemotherapy may be required, and will be performed according to *Table 5.2.2.* Chemotherapy dose reductions are permanent; once the dose of any chemotherapy agent is reduced, it may not be re-escalated in subsequent cycles. The dose reductions for each agent in the platinum-based doublet chemotherapy regimen are not linked and may be adjusted independently as summarized below.

Table 5.2.2 Dose modifications for Chemotherapeutic Agents

Drug	Dose level 0	Dose level -1	Dose level -2	Dose level -3
Carboplatin	AUC 5	AUC 4	AUC 3	Discontinue
Etoposide	100 mg/m2	80 mg/m2	60 mg/m2	Discontinue

Any participants with two prior dose reductions for one agent who experiences a toxicity that would cause a third dose reduction must be discontinued from that agent.

5.2.2.1 Dose Reductions for Hematologic Adverse Events

Dose modifications for hematologic toxicities (according to CTCAE version 5.0) are summarized in *Table-5.2.2.1*.

Dose adjustments are based on nadir blood counts (assessed as per local standards) since the preceding drug administration. Dose level adjustments for platinum-based doublet chemotherapy are relative to that of the preceding administration. Generally, both chemotherapy agents in the platinum-based doublet chemotherapy regimen should be dose reduced together for hematologic toxicity. After the first cycle, growth factors may be used to assist hematologic recovery. Use local standards of care in the use of these supportive measures. Additionally, prophylactic antibiotics may be used according to local standards of care. Please report any antibiotic or growth factor use on the eCRF.

Table 5.2.2.1 Dose modifications for carboplatin/etoposide hematologic adverse events

Toxicity	Grade	Action	Treatment Restart	Treatment discontinuation
Neutropenia	3	Hold treatment until Neutrophil count resolves to >1,500/mm3	No dose reduction required if neutropenia does not induce treatment delay. If neutropenia induces treatment delay consider G-CSF support. If already con G-CSF support reduce by 1 DL (at the discretion of the investigator)	Toxicity does not resolve within 6 weeks of last infusion or if > 2 Dose Level reductions are exceeded
	4	Hold treatment until neutrophil count resolves to >1,500/mm^3	Reduce by 1 DL *consider G-CSF	
Febrile Neutropenia	3-4	Hold treatment until toxicity resolves to Grade 0-1	Reduce by 1 DL	Toxicity does not resolve within 6 weeks of last infusion or if > 2 Dose Level reductions are exceeded
Thrombocytopenia	3-4	Platelet count resolves to >100,000/mm3	Reduce by 1 DL	Toxicity does not resolve within 6 weeks of last infusion or if > 2 Dose Level reductions exceeded

5.2.2.2. Dose Reductions for Non-Hematologic Adverse Events

Dose adjustments for chemotherapy for non-hematologic toxicities during treatment are described in Section 5.2.2. All dose reductions should be made based on the worst grade toxicity during the prior cycle. Participants experiencing any toxicity during the previous cycle should have their chemotherapy delayed up to 3 weeks (6 weeks since the administration of the last chemotherapy infusion) until retreatment criteria are met and then reduced for all subsequent cycles by 1 dose level or discontinued as appropriate. Dose levels for the two drugs in the platinum-doublet chemotherapy regimen are not linked and may be reduced independently, as summarized in *Table-5.2.2.2.*

Table 5.2.2.2. Dose modifications for non-hematologic adverse events

Toxicity	Grade	Action	Treatment Restart
Creatinine increased	2	Hold treatment until toxicity resolves to Grade 0-1	Reduce cisplatin by 1 DL
	3-4[1]	Discontinue carboplatin if creatinine clearance <20 ml/min	Discontinue treatment
Ototoxicity or sensory neuropathy	2	Hold treatment until toxicity resolves to Grade 0-1	Reduce cisplatin by 1 DL
	≥ 3	Discontinue chemotherapy	Discontinue chemotherapy
Allergic reaction [a]	≥ 3	Discontinue chemotherapy	Discontinue chemotherapy
All other non-hematological toxicities	3-4	Hold treatment until toxicity resolves to Grade 0-1.	Reduce by 1 DL

[a] Only the drug(s) causing the hypersensitivity reaction or acute infusion reaction (≥ Grade 3) require discontinuation. All other drugs may be continued.

5.2.3. Dose Delay Criteria

5.2.3.1. Dose delay Criteria for Nivolumab

Tumor assessments for all participants should continue as per protocol even if dosing is delayed.

Nivolumab administration should be delayed for the following:

- Any Grade ≥ 2 non-skin, drug-related adverse event, except for fatigue and laboratory abnormalities.
- Any Grade ≥ 3 skin drug-related AE.
- Any Grade ≥ 3 drug-related laboratory abnormality with the following exceptions for lymphopenia, AST, ALT, or total bilirubin or asymptomatic amylase or lipase:
    - Grade 3 lymphopenia does not require a dose delay

If a participant has a baseline AST, ALT, or total bilirubin that is within normal limits, delay dosing for drug-related Grade 2 toxicity.

If a participant has baseline AST, ALT, or total bilirubin within the Grade 1 toxicity range, delay dosing for drug-related Grade ≥ 3 toxicity.

- Any Grade ≥ 3 drug-related amylase or lipase abnormality that is not associated with symptoms or clinical manifestations of pancreatitis does not require dose delay.
- Any AE, laboratory abnormality or intercurrent illness, which, in the judgment of the investigator, warrants delaying the dose of study medication.

Participants who require delay should be re-evaluated weekly or more frequently if clinically indicated. Participants should resume dosing when re-treatment criteria are met.

5.2.3.2. Dose delay Criteria for Chemotherapy

Chemotherapy drugs should be delayed for any of the following on the Day 1 of each cycle:

- Absolute neutrophil count (ANC) < 1500/L
- Platelets < 100,000/mm3
- Any Grade ≥ 2 non-skin, non-hematologic, drug-related adverse event (excluding Grade 2 alopecia, Grade 2 fatigue, and Grade 2 laboratory abnormalities)
- Any Grade ≥ 3 skin, drug-related adverse event
- Any Grade ≥ 3 drug-related laboratory abnormality, with the following exceptions for lymphopenia, AST, ALT, or total bilirubin:
    - Grade 3 lymphopenia does not require dose delay.
    - If a participant has a baseline AST, ALT or total bilirubin that is within normal limits, delay dosing for drug-related Grade ≥ 2 toxicity.

  o If a participant has baseline AST, ALT, or total bilirubin within the Grade 1 toxicity range, delay dosing for drug-related Grade ≥ 3 toxicity.
- Any adverse event, laboratory abnormality, or intercurrent illness, which, in the judgment of the investigator, warrants delaying the dose of study medication.

Investigators should consult local labeling for the chemotherapy drugs being administered to any given participant for additional guidance on dose delays.

If any non-hematologic adverse event meeting the dose delay criteria above is felt to be related to only one particular agent in the platinum-based doublet chemotherapy regimen, then that agent alone may be omitted for that cycle while the other agent is given. In order to maintain synchronized dosing of the regimen, the omitted agent should be resumed with the next scheduled cycle once the AE has improved and retreatment criteria are met. Please refer to *Section 5.2.2* to determine if dose reduction of the resumed agent is required.

If both drugs in the platinum-based doublet chemotherapy regimen are delayed, then the participant should be re-evaluated weekly or more frequently if clinically indicated until retreatment criteria are met (as per *Section 5.2.4*).

<u>5.2.4. Criteria to Resume Dosing</u>

5.2.4.1. Criteria to Resume Nivolumab Dosing

Participants may resume treatment with nivolumab when the drug-related AE(s) resolve(s) to Grade ≤ 1 or baseline, with the following exceptions:

- Participants may resume treatment in the presence of Grade 2 fatigue.
- Participants who have not experienced a Grade 3 drug-related skin AE may resume treatment in the presence of Grade 2 skin toxicity.

- Participants with baseline Grade 1 AST/ALT or total bilirubin who require dose delays for reasons other than a 2-grade shift in AST/ALT or total bilirubin may resume treatment in the presence of Grade 2 AST/ALT OR total bilirubin.
- Participants with combined Grade 2 AST/ALT and total bilirubin values meeting discontinuation parameters (*Section 6.1.1*) should have treatment permanently discontinued.
- Drug-related pulmonary toxicity, diarrhea, or colitis must have resolved to baseline before treatment is resumed. Participants with persistent Grade 1 pneumonitis after completion of a steroid taper over at least 1 month may be eligible for retreatment if discussed with and approved by the BMS Medical Monitor.
- Participants who received systemic corticosteroids for management of any drug-related toxicity must be off corticosteroids or have tapered down to an equivalent dose of prednisone ≤ 10 mg/day.
- Drug-related endocrinopathies adequately controlled with only physiologic hormone replacement may resume treatment.
- Dose delay of nivolumab which results in treatment interruption of > 6 weeks requires treatment discontinuation, with exceptions as noted in *Section 6.1.1*

5.2.4.2. Criteria to Resume Treatment with Chemotherapy

- Participants may resume treatment with chemotherapy when the ANC returns to 1500/µl, the platelet count returns to 100,000/mm3, and all other drug-related toxicities have returned to baseline or Grade 1 (or Grade 2 for alopecia and fatigue).
- If a participant fails to meet criteria for re-treatment, then re-treatment should be delayed, and the participant should be re-evaluated weekly or more frequently as clinically indicated. Any participant who fails to recover from toxicity attributable to chemotherapy to baseline or Grade 1 (except Grade 2 alopecia and fatigue) within 6 weeks from the last dose given should discontinue the drug(s) that caused the delay.
- When resuming chemotherapy treatment, please follow the dose reduction recommendations in *Section 5.2.2.*

5.3. Duration of Therapy

- During the "induction phase" six cycles of chemotherapy in combination with nivolumab will be given unless disease progression (see definition Section 7) or unacceptable toxicity are encountered.
- If no progression or unacceptable toxicity are observed after the induction phase, the patient will continue to receive nivolumab 480 mg every 4 weeks. The maximum treatment duration is 24 months or 34 cycles including the induction phase.
- <u>End of treatment</u>
  Patients will continue study treatment until disease progression per RECIST 1.1 or, unacceptable toxicity, withdrawal of consent, physician's decision, subject/guardian decision, lost to follow-up, death, study treatment completion (24 months) or the study is terminated by the Sponsor.

<u>5.3.1. Treatment Beyond Disease Progression</u>

Accumulating evidence indicates a minority of participants treated with immunotherapy may derive clinical benefit despite initial evidence of PD.

Participants will be permitted to continue on nivolumab for treatment beyond initial RECIST 1.1 defined PD as long as they meet the following criteria:

- Investigator-assessed clinical benefit and no rapid disease progression.
- Participant is tolerating study treatment.
- Stable performance status.
- Treatment beyond progression will not delay an imminent intervention to prevent serious complications of disease progression (eg, CNS metastases).
- Participant provides written informed consent prior to receiving additional nivolumab treatment, using an ICF describing any reasonably foreseeable risks or discomforts, or other alternative treatment options.

The decision to continue treatment beyond initial investigator-assessed progression should be discussed with the BMS Medical Monitor and documented in the study records. A follow-up scan should be performed within six weeks +/- 7 days of original PD to determine whether there has been a decrease in the tumor size, or continued progression of disease. Subsequent scans should be performed per protocol defined schedule +/- 7 days until further progression is determined.
If the investigator feels that the participant continues to achieve clinical benefit by continuing treatment, the participant should remain on the trial and continue to receive monitoring according to the Time and Events Schedule in Section *7.2.*

For the participants who continue study therapy beyond progression, further progression is defined as an additional 10% increase in tumor burden from time of initial PD. This includes an increase in the sum of diameters of all target lesions and/or the diameters of new measurable lesions compared to the time of initial PD. Nivolumab treatment should be discontinued permanently upon documentation of further progression.

New lesions are considered measurable at the time of initial progression if the longest diameter is at least 10 mm (except for pathological lymph nodes which must have a short axis of at least 15 mm).
Any new lesion considered non-measurable at the time of initial progression may become measurable and therefore included in the tumor burden if the longest diameter increases to at least 10 mm (except for pathological lymph nodes which must have a short axis of at least 15 mm). In situations where the relative increase in total tumor burden by 10% is solely due to inclusion of new lesions which become measurable, these new lesions must demonstrate an absolute increase of at least 5 mm.

5.4. Preparation/Handling/Storage/ Accountability

The investigational product should be stored in a secure area according to local regulations. It is the responsibility of the investigator to ensure that investigational product is only dispensed to study participants. The investigational product must be dispensed only from official study sites by authorized personnel according to local regulations.

The product storage manager should ensure that the study treatment is stored in accordance with the environmental conditions (temperature, light, and humidity) as determined by BMS. If concerns regarding the quality or appearance of the study treatment arise, the study treatment should not be dispensed and contact BMS immediately.

Study treatment not supplied by BMS will be stored in accordance with the package insert.

Investigational product documentation (whether supplied by BMS or not) must be maintained that includes all processes required to ensure drug is accurately administered. This includes documentation of drug storage, administration and, as applicable, storage temperatures, reconstitution, and use of required processes (eg, required diluents, administration sets).

5.5. Concomitant therapy

5.5.1. Prohibited and/or Restricted Therapy

Subjects are prohibited from receiving the following therapies during screening to the end of treatment of this trial:

- Antineoplastic systemic chemotherapy or biological therapy not specified in the protocol
- Immunotherapy not specified in this protocol.
- Investigational agents other than nivolumab.
- Radiation therapy; palliative radiation therapy to a symptomatic lesion (e.g. bony metastasis), or to the brain may be permitted.
- Live vaccines within 30 days prior to the first dose of trial treatment and while participating in the trial. Live vaccines include, but are not limited to, the following: measles, mumps, rubella, chicken pox, yellow fever, rabies, BCG, and typhoid (oral) vaccine. The killed virus vaccines used for seasonal influenza vaccines for injection are allowed; however live attenuated intranasal influenza vaccines (e.g. Flu - Mist®) are not allowed.
- The use of systemic steroid therapy and other immunosuppressive drugs is not allowed except for the treatment of infusion reaction, irAEs, and for prophylaxis against imaging contrast dye allergy or replacement-dose steroids in the setting of adrenal insufficiency (providing this is <10 mg/day prednisone or equivalent), or transient exacerbations of other underlying diseases such as COPD requiring treatment for ≤ 3 weeks. If systemic corticosteroids are required for the control of infusion reactions or irAEs, it must be tapered and be at non immunosuppressive doses (< 10 mg/day of prednisone or equivalent) before the next administration of study treatment. If the dose of prednisone or equivalent cannot be reduced to less than 10 mg/day before the administration of next dose of study treatment then nivolumab must be discontinued.

Caution should be used regarding the use of herbal medications as there may be yet unknown interactions with nivolumab. Discontinuation of the use of herbal medications prior to study enrollment is encouraged. Except for the permitted procedures specified as palliative local therapies (Section 5.5.3), all other radiation therapy or surgery to any tumor lesion is not permitted during study treatment.

There are no prohibited therapies during the post-treatment follow-up phase.

5.5.2. Permitted Therapy

All treatments that the investigator considers necessary for a subject's welfare may be administered at the discretion of the investigator in keeping with the standards of medical care.

Participants are permitted the use of topical, ocular, intra-articular, intranasal, and inhalational corticosteroids (with minimal systemic absorption). Adrenal replacement steroid doses >10 mg daily prednisone are permitted. A brief (less than 3 weeks) course of corticosteroids for prophylaxis (eg,

contrast dye allergy) or for treatment of non-autoimmune conditions (eg, delayed-type hypersensitivity reaction caused by a contact allergen) is permitted.

All concomitant medication will be recorded on the case report form (CRF) including all prescription, over-the-counter (OTC), herbal supplements, and IV medications and fluids.

If changes occur during the trial period, documentation of drug dosage, frequency, route, and date may also be included on the CRF.

All concomitant medications received during the trial through 30 days after the last dose of trial treatment should be recorded.

5.5.3. Palliative Local Therapy

Palliative local therapy, including palliative radiation therapy and palliative surgical resection, to symptomatic non-target bone lesions, skin lesions, or CNS lesions is permitted prior to discontinuation of study treatment for participants who do not have evidence of overall clinical or radiographic progression per RECIST 1.1.

Palliative local therapy to lesions causing gastrointestinal bleeding may also be permitted prior to discontinuation of study treatment in participants who do not have evidence of overall clinical or radiographic progression per RECIST 1.1, provided that the lesions undergoing palliative local therapy are not the only sites of measurable disease and the case is discussed with and approved by the BMS Medical Monitor.

Participants requiring palliative local therapy should be evaluated for objective evidence of disease progression prior to the initiation of such therapy, particularly if the most recent tumor assessment was more than 4 weeks prior to the start of local therapy. If progression per RECIST 1.1 is identified on any tumor assessments prior to the initiation of palliative local therapy, then participants must either discontinue study drug treatment or they must meet criteria to continue treatment beyond progression (*Section 5.3.1*) in order to resume immunotherapy after palliative local therapy.

The potential for overlapping toxicities with radiotherapy and nivolumab currently is not known; however, anecdotal data suggests that it is tolerable. As concurrent radiotherapy and nivolumab have not been formally evaluated, in cases where palliative radiotherapy is required for a tumor lesion, then nivolumab should be withheld for at least 1 week before, during, and 1 week after radiation. Participants should be closely monitored for any potential toxicity during and after receiving radiotherapy, and AEs should resolve to Grade ≤ 1 prior to resuming nivolumab.

5.5.4. Imaging Restrictions and Precautions

It is the local imaging facility's responsibility to determine, based on participant attributes (eg, allergy history, diabetic history and renal status), the appropriate imaging modality and contrast regimen for each participant. Imaging contraindications and contrast risks should be considered in this assessment. Participants with renal insufficiency should be assessed as to whether or not they should receive contrast and if so, what type and dose of contrast is appropriate. Should a participant have a contraindication for CT IV contrast, a non-contrast CT of the chest and a contrast enhanced MRI of the abdomen and pelvis may be obtained.

Specific to MRI, participants with severe renal insufficiency (ie, estimated glomerular filtration rate (eGFR) < 30 mL/min/1.73 m2) are at increased risk of nephrogenic systemic fibrosis. MRI contrast should not be given to this participant population. In addition, participants are excluded from MRI if they have tattoos, metallic implants, pacemakers, etc. The ultimate decision to perform

MRI in an individual participant in this study rests with the site radiologist, the investigator and the standard set by the local Ethics Committee.

6. Discontinuation Criteria

6.1. Discontinuation from Study Treatment

For all participants, global deterioration of health status requiring discontinuation of treatment without objective evidence of disease progression at that time should be reported as ‘symptomatic deterioration’ in the source data and in the case report form.

Tumor assessments for participants, who discontinue study treatment without radiographic progression, should continue as per protocol until radiographic progression is determined.

Chemotherapy dose reduction is allowed on study. Any participant with two prior dose reductions to one agent who experiences a toxicity that would cause a third dose reduction must be discontinued from that agent. A participant who is discontinued from the chemotherapy treatment will remain on the study and receive nivolumab.

6.1.1. Nivolumab Dose Discontinuation

Treatment with nivolumab should be permanently discontinued for any of the following:

- Any Grade ≥ 2 drug-related uveitis or eye pain or blurred vision that does not respond to topical therapy and does not improve to Grade 1 severity within the re-treatment period OR requires systemic treatment.
- Any Grade ≥ 2 drug-related pneumonitis or interstitial lung disease that does not resolve after a dose delay and systemic steroids (also see *Table 5.2.1).*
- Any Grade 3 drug-related bronchospasm, hypersensitivity reaction, or infusion reaction, regardless of duration;
- Any Grade 3 non-skin, drug-related adverse event lasting > 7 days, with the following exceptions for uveitis, pneumonitis, bronchospasm, diarrhea, colitis, neurologic toxicity, hypersensitivity reactions, infusion reactions, endocrinopathies, and laboratory abnormalities:
    - Grade 3 drug-related uveitis, pneumonitis, bronchospasm, diarrhea, colitis, neurologic toxicity, hypersensitivity reaction, or infusion reaction of any duration requires discontinuation.
    - Grade 3 drug-related endocrinopathies adequately controlled with only physiologic hormone replacement do not require discontinuation.
    - Grade 3 drug-related laboratory abnormalities do not require treatment discontinuation except:
        - Grade 3 drug-related thrombocytopenia > 7 days or associated with bleeding requires discontinuation.
        - Any drug-related liver function test (LFT) abnormality that meets the following criteria require discontinuation: Grade ≥ 3 drug-related AST, ALT or Total Bilirubin requires discontinuation.*

    * In most cases of Grade 3 AST or ALT elevation, study drugs(s) will be permanently discontinued. If the investigator determines a possible favorable benefit/risk ratio that warrants continuation of study drugs(s), a discussion between the investigator and the BMS medical monitor or designee must occur.

    Concurrent AST or ALT > 3x ULN and total bilirubin > 2x ULN
- Any Grade 4 drug-related adverse event or laboratory abnormality, except for the following events, which do not require discontinuation:
    - Grade 4 neutropenia ≤ 7 days.

    - Grade 4 lymphopenia or leukopenia.
    - Isolated Grade 4 amylase or lipase abnormalities that are not associated with symptoms or clinical manifestations of pancreatitis and decrease to < Grade 4 within 1 week of onset.
    - Isolated Grade 4 electrolyte imbalances/abnormalities that are not associated with clinical sequelae and are corrected with supplementation/appropriate management within 72 hours of their onset.
- Grade 4 drug-related endocrinopathy adverse events such as adrenal insufficiency, ACTH deficiency, hyper- or hypothyroidism, or glucose intolerance, which resolve or are adequately controlled with physiologic hormone replacement (corticosteroids, thyroid hormones) or glucose controlling agents, respectively, may not require discontinuation after discussion with and approval from the BMS Medical Monitor.
- Dosing delays lasting > 6 weeks from the previous dose that occur for non-drug-related reasons may be allowed if approved by the BMS medical monitor. Prior to re-initiating treatment in a participant with a dosing delay lasting > 6 weeks, the BMS medical monitor must be consulted. Tumor assessments should continue as per protocol even if dosing is delayed. Periodic study visits to assess safety and laboratory studies should also continue every 6 weeks or more frequently if clinically indicated during such dosing delays.
- Any adverse event, laboratory abnormality, or intercurrent illness which, in the judgment of the Investigator, presents a substantial clinical risk to the participant with continued nivolumab dosing.

The assessment for discontinuation of nivolumab should be made separately from the assessment made for discontinuation of chemotherapy.

If a participant meets criteria for discontinuation and investigator is unable to determine whether the event is related to nivolumab or chemotherapy, the participant should discontinue all treatment.

The assessment for discontinuation of nivolumab should be made separately from the assessment made for discontinuation of chemotherapy doublet. If criteria for discontinuation for nivolumab are met before the nivolumab plus platinum-based doublet chemotherapy cycles have been completed, platinum-based doublet chemotherapy may continue until 6 cycles have been completed.

6.1.2. Chemotherapy Dose Discontinuation

Except where specified below, chemotherapy drugs in the platinum-based doublet chemotherapy regimen should be discontinued for any of the following:

- Any Grade ≥ 3 peripheral neuropathy
- Any drug-related liver function test (LFT) abnormality that meets the following criteria requires discontinuation:
    - AST or ALT > 5-10 x ULN for > 2 weeks
    - AST or ALT > 10 x ULN
    - Total bilirubin > 5 x ULN
    - Concurrent AST or ALT > 3x ULN and total bilirubin > 2 x ULN
- Any drug-related adverse event which recurs after two prior dose reductions for the same drug-related adverse event requires discontinuation of the drug(s) which was/were previously dose reduced.

- Any Grade ≥ 3 drug-related hypersensitivity reaction or infusion reaction requires discontinuation of the drug(s) felt to be causing the reaction. The drug not felt to be related to the hypersensitivity reaction or infusion reaction may be continued.
- Any Grade 4 drug-related adverse event which the investigator deems is inappropriate to be managed by dose reduction(s) requires discontinuation of the drug(s) felt to be causing the event. The drug not felt to be related to the event may be continued.
- Any event that leads to delay in dosing of any study drug(s) for > 6 weeks from the previous dose requires discontinuation of that drug(s) with the following exception:
    - Dosing delays lasting > 6 weeks from the previous dose that occur for non-drug-related reasons may be allowed if approved by the BMS medical monitor. Prior to re-initiating treatment in a participant with a dosing delay lasting > 6 weeks, the BMS medical monitor must be consulted. Periodic study visits to assess safety and laboratory studies should also continue every 6 weeks or more frequently if clinically indicated during such dosing delays.
- Any adverse event, laboratory abnormality, or intercurrent illness which, in the judgment of the Investigator, presents a substantial clinical risk to the participant with continued platinum-based doublet chemotherapy dosing. Investigators should consult local labeling for the chemotherapy drugs being administered to any given participant for additional guidance on dose discontinuation.

6.2. Discontinuation from Study

Participants who request to discontinue study treatment will remain in the study and must continue to be followed for protocol specified follow-up procedures. The only exception to this is when a participant specifically withdraws consent for any further contact with him/her or persons previously authorized by participant to provide this information.

- Participants should notify the investigator of the decision to withdraw consent from future follow-up in writing, whenever possible.
- The withdrawal of consent should be explained in detail in the medical records by the investigator, as to whether the withdrawal is from further treatment with study treatment only or also from study procedures and/or post treatment study follow-up, and entered on the appropriate CRF page.
- In the event that vital status (whether the participant is alive or dead) is being measured, publicly available information should be used to determine vital status only as appropriately directed in accordance with local law.
- If the participant withdraws consent for disclosure of future information, the sponsor may retain and continue to use any data collected before such a withdrawal of consent.

In the case of pregnancy, the investigator must immediately notify the BMS Medical Monitor/designee of this event. In the event a normal healthy female participant becomes pregnant during a clinical trial, the study treatment must be discontinued immediately. In most cases, the study treatment will be permanently discontinued in an appropriate manner (eg, dose tapering if necessary for participant safety). Please call the BMS Medical Monitor within 24 hours of awareness of the pregnancy. If the investigator determines a possible favorable benefit/risk ratio that warrants

continuation of study treatment, a discussion between the investigator and the BMS Medical Monitor/designee must occur.

All participants who discontinue study treatment should comply with protocol specified follow-up procedures as outlined in *Section 7*. The only exception to this requirement is when a participant withdraws consent for all study procedures including post-treatment study follow-up or loses the ability to consent freely (ie, is imprisoned or involuntarily incarcerated for the treatment of either a psychiatric or physical illness).

If study treatment is discontinued prior to the participant's completion of the study, the reason for the discontinuation must be documented in the participant's medical records and entered on the appropriate case report form (CRF) page.

6.2.1. Post Study Treatment Study Follow-up

In this study, OS is a key endpoint of the study. Post study follow-up is of critical importance and is essential to preserving participant safety and the integrity of the study. Participants who discontinue study treatment must continue to be followed for collection of outcome and/or survival follow-up data as required and in line with *Section 7* until death or the conclusion of the study.

6.3. Lost to Follow-up

All reasonable efforts must be made to locate participants to determine and report their ongoing status. This includes follow-up with persons authorized by the participant.

Lost to follow-up is defined by the inability to reach the participant after a minimum of three documented phone calls, faxes, or emails as well as lack of response by participant to one registered mail letter. All attempts should be documented in the participant's medical records.

If it is determined that the participant has died, the site will use permissible local methods to obtain date and cause of death.

If investigator's use of third party representative to assist in the follow-up portion of the study has been included in the participant's informed consent, then the investigator may use a Sponsor retained third party representative to assist site staff with obtaining participant's contact information or other public vital status data necessary to complete the follow-up portion of the study.

The site staff and representative will consult publicly available sources, such as public health registries and databases, in order to obtain updated contact information.

If after all attempts, the participant remains lost to follow-up, then the last known alive date as determined by the investigator should be reported and documented in the participant's medical records.

7. Clinical evaluation, laboratory tests, follow-up

Study procedures and timing are summarized in the Schedule of Activities.

- All immediate safety concerns must be discussed with the Sponsor immediately upon occurrence or awareness to determine if the participant should continue or discontinue treatment.
- Adherence to the study design requirements, including those specified in the Schedule of Activities, is essential and required for study conduct.
- All screening evaluations must be completed and reviewed to confirm that potential participants meet all eligibility criteria before randomization. The investigator will maintain a screening log to record details of all participants screened and to confirm eligibility or record reasons for screening failure, as applicable.

7.1. Before treatment start

7.1.1. Informed Consent

The investigator or qualified designee must obtain documented consent from each potential subject or each subject’s legally acceptable representative prior to participating in a clinical trial or Future Biomedical Research.

7.1.1.1. General Informed Consent

- Consent must be documented by the subject’s dated signature or by the subject’s legally acceptable representative dated signature on a consent form along with the dated signature of the person conducting the consent discussion.
- A copy of the signed and dated consent form should be given to the subject before participation in the trial. The initial informed consent form, any subsequent revised written informed consent form and any written information provided to the subject must receive the IRB/ERC’s approval/favorable opinion in advance of use.
- The subject or his/her legally acceptable representative should be informed in a timely manner if new information becomes available that may be relevant to the subject’s willingness to continue participation in the trial. The communication of this information will be provided and documented via a revised consent form or addendum to the original consent form that captures the subject’s dated signature or by the subject’s legally acceptable representative dated signature.
- The informed consent will adhere to IRB/ERC requirements, applicable laws and regulations.

7.1.1.2. Consent and Collection of Specimens for Future Biomedical Research

- The investigator or qualified designee will explain the Future Biomedical Research consent to the subject, answer all of his/her questions, and obtain written informed consent before performing any procedure related to the Future Biomedical Research sub-trial.
- A copy of the informed consent will be given to the subject.

7.1.2. Inclusion/Exclusion Criteria Assessment

All inclusion and exclusion criteria will be reviewed by the investigator or qualified designee to ensure that the subject qualifies for the trial. More details on criteria for patients eligibility section 4.1 and 4.2.

7.1.3. Medical History

- A medical history will be obtained by the investigator or qualified designee. Medical history will include all active conditions, and any condition diagnosed within the prior 10 years that are considered to be clinically significant by the Investigator.
- Any autoimmune disorders, regardless of onset date, should be recorded.

7.1.4. Prior and concomitant medications review

7.1.4.1. Prior Medications

The investigator or qualified designee will review prior medication use, including any protocol-specified washout requirement, and record prior medication taken by the subject within 30 days of first dose.

7.1.4.2. Concomitant Medications

- The investigator or qualified designee will record medication, if any, taken by the subject during the trial from the time of signing the informed consent form until the Safety Follow-up Visit.
- All medications related to reportable SAEs should be recorded.

7.1.5. Assignment of Screening Number

- All consented subjects will be given a unique screening number that will be used to identify the subject for all procedures that occur.
- Each subject will be assigned only one screening number. Screening numbers must not be re-used for different subjects. Any subject who is screened multiple times will retain the original screening number assigned at the initial screening visit.

7.1.6. Subject Identification Card

- All subjects will be given a Subject Identification Card identifying them as participants in a research trial. The card will contain trial site contact information (including direct telephone numbers) to be utilized in the event of an emergency.
- The investigator or qualified designee will provide the subject with a Subject Identification Card immediately after the subject provides written informed consent.

7.1.7. Submitting Tumor Sample

- Tumor tissue sample is mandatory to participate in this trial.
- Either pre-existing archived or newly-obtained (fresh tissue) biopsy specimens from either primary or metastatic tumor, whichever is most recent.

- Newly-obtained (fresh tissue) is defined as a specimen obtained up to 42 days prior to administration of study treatment on Day 1 of Cycle 1, and no additional anti-cancer treatment has been given after the specimen was obtained.
- Pre-existing, archived tissue must be obtained prior to time point that any anti-cancer treatment was given.

- A fine needle aspirate (FNA) or cytologic specimen will not be acceptable. In the event the most recent available tumor tissue specimen is an FNA or cytologic specimen, a previous specimen obtained prior to any anti-cancer therapy was given may be submitted (provided it is not an FNA or cytologic specimen).
- Where available, both newly-obtained (fresh tissue) and pre-existing archived tissues are requested.
- Tumor tissue specimen submitted in either formalin solution or FFPE block is acceptable.

7.1.8. Tumor Imaging

- To meet screening criteria, initial tumor imaging must be performed within 28 days prior to the beginning of the treatment. This scan will be considered the baseline assessment for the study. Scans performed as part of routine clinical management are acceptable for use as the baseline scan if they are of diagnostic quality, include all required anatomy, and performed within 28 days prior to the beginning of the treatment.
- The site study team must review pre-trial images to confirm the subject has at least one target lesion (i.e. meets measurability requirements) per RECIST 1.1.
- The following assessments are required at screening:

7.1.8.1. Computed (CT) with IV contrast or Magnetic Resonance Imaging (MRI) of chest, abdomen and pelvis.

- The preferred radiologic technique is CT with intravenous (IV) contrast. If a patient is known to have a contraindication to CT contrast or develops a contraindication during the trial, a non-contrast CT of the chest (MRI is not recommended due to respiratory artifacts) plus a contrast-enhanced MRI (if possible) of the abdomen and pelvis should be performed.

7.1.8.2. Brain CT with IV contrast or MRI scan

Patients with known baseline brain lesions are not eligible for this study, but brain CT scan or MRI is not required neither at baseline nor in the follow-up in the absence of neurological signs or symptoms.

7.1.8.3. Whole body bone scan

This is not required at any time point unless clinically indicated.

7.1.9. Laboratory Assessments

- Laboratory tests for screening should be performed within 7 days prior to study enrollment. If performed < 7 days prior to the first dose of trial therapy the screening laboratory tests will serve as cycle 1 day 1 laboratory tests. If not, laboratory tests will need to be performed again on day 1 of cycle 1.

- Serum and plasma samples for optional biomarker studies will be collected prior to the beginning of treatment.

A summary of the procedures required is summarized in Table 1 and flow chart- Section 7.4.

7.1.10. 12-Lead Electrocardiogram (ECG)

A standard 12-lead ECG will be performed using local standard procedures once at screening. Clinically significant abnormal findings should be recorded as medical history and monitored if needed. No new ECGs will be performed unless clinically indicated.

7.2. During treatment

7.2.1. Adverse Event (AE) Monitoring

- The investigator or qualified designee will assess each subject to evaluate for potential new or worsening AEs as specified in the Trial Flow Chart and more frequently if clinically indicated.
- Adverse events will be graded and recorded throughout the trial and during the follow-up period according to NCI CTCAE Version 5.0. Toxicities will be characterized in terms regarding seriousness, causality, toxicity grading, and action taken with regard to trial treatment.
- All AEs of unknown etiology associated with nivolumab exposure should be evaluated to determine if it is possibly an ECI of a potentially immunologic etiology (termed immune-related adverse events, or irAEs).

7.2.2. Physical Exam

The investigator or clinical designee will perform a complete physical exam during the screening period. Clinically significant abnormal findings should be recorded as medical history.
Additional full physical exams should be performed as specified in the Trial Flow Chart-Section 7.4. After the first dose of trial treatment new clinically significant abnormal findings should be recorded as AEs.

7.2.3. Vital Signs

- The investigator or qualified designee will take vital signs at screening, prior to the administration of each dose of trial treatment and at treatment discontinuation as specified in the Trial Flow Chart-Section 7.4.
- Vital signs should include temperature, pulse, respiratory rate, weight and blood pressure. Height will be measured at screening only.

7.2.4. 12-Lead Electrocardiogram (ECG)

No ECGs will be performed unless clinically indicated.

7.2.5. Eastern Cooperative Oncology Group (ECOG) Performance Status

The investigator or qualified designee will assess ECOG status (see appendix) at screening, prior to dosing on day 1 of each treatment cycle and at discontinuation of trial treatment as specified in the Trial Flow Chart.

7.2.6. Tumor Imaging and Assessment of Disease

- Tumor imaging will be performed by whole body contrast-enhanced computed tomography (CT; preferred).
- The first on-study imaging assessment should be performed at 8 weeks (± 7 days) after the first dose of trial treatment. Subsequent imaging should be performed every 8 weeks (± 7 days) during the first 12 months and every 12 weeks thereafter until PD. Imaging may be more frequently performed if clinically indicated.

7.2.7. Laboratory Assessments

- After Cycle 1, pre-dose laboratory procedures can be conducted within 72 hours prior to dosing. Results must be reviewed by the investigator or qualified designee and found to be acceptable prior to each dose of trial treatment.
- The main laboratory determinations are summarized in Table 7

Table 7. Laboratory determinations required during the study

Test category	Determinations
Hematology	Hematocrit, Hemoglobin, Platelets, Red blood cells, White blood cells, Differential (Basophils, Eosinophils, Lymphocytes, Monocytes, Neutrophils, Bands, Other).
Chemistry	Albumin, Alkaline phosphatase, ALT, AST, Gamma-glutamyl-transferase (GGT), Lactate Dehydrogenase (LDH), Calcium, Magnesium, Phosphorus,, Sodium, Potassium, Creatinine, Creatine kinase, Direct Bilirubin, Indirect Bilirubin, Total Bilirubin,,, Total Protein, Blood Urea Nitrogen (BUN) or Urea, Uric Acid, Amylase, Lipase, Glucose.
Coagulation	**At Screening:** Prothrombin time (PT), International normalized ratio (INR]), Activated partial thromboplastin time (APTT).
Thyroid	**At screening**: TSH (Thyroid Stimulating Hormone), Free T3 and Free T4.<br>At the subsequent visits as indicated in the flow chart: TSH only.<br>If TSH is abnormal, Free T3 and T4 shall be tested.
Hepatitis markers	**At screening**: HBV-DNA, HBsAg, HBsAb, HBcAb, HCV RNA-PCR.
Cytokines	IFN-γ, IL-6, IL-1,TNF-α.
Tumor markers	**At screening**: NSE, CGA.<br>**On treatment:** every 2 cycles if elevated at baseline.

Urinalysis	**At Screening**: Macroscopic Panel (Dipstick) (Color, Bilirubin, Blood, Glucose, Ketones, Leukocytes esterase, Nitrite, pH, Protein, Specific Gravity, Urobilinogen)<br>If dipstick is abnormal then perform local laboratory Microscopic Panel (Red Blood Cells, White Blood Cells, Casts, Crystals, Bacteria, Epithelial cells).
Pregnancy test	A serum pregnancy test must be performed at screening (at the local laboratory) within ≤72 hours before first dose of study treatment.

7.2.8. Serum/Urine β-hCG

- All women who are being considered for participation in the trial, and who are not surgically sterilized or postmenopausal (defined as: a woman who is ≥45 years of age and has not had menses for more than 1 year), will be tested for pregnancy within 72 hours prior to each cycle of trial treatment and 30 days post treatment.
- Subjects must be excluded/discontinued in the event of a positive or borderline-positive test result. If a urine test is positive or borderline a serum β-HCG test will be required. The results of the pregnancy testing will not be recorded.

7.3. After the end of treatment (Follow-up)

- After the end of the treatment clinical procedures and assessment will be performed as previously explained and is reflected in the summary table (section 7.4).
- Serum and plasma samples for optional biomarker studies will also be collected at this point.

7.4. Summary table

Table 7.4. Trial flow chart

	**Screening phase**	**Treatment cycles [a] (chemotherapy + nivolumab)**						**Maintenance phase Cycles**		**End of treatment**	**Post-treatment**		
	Pre-Study	1	2	3	4	5	6	1	2 and beyond	Time of treatment discontinuation	Safety follow up	Follow up visits	Survival follow up [o]
											30 days after last dose	Every 12 w after last dose	Every 12w
Scheduling window (days)	-28 to -1		±3	±3	±3	±3	±3	±3	±3	±3	±7	±7	±7
Study Drug Admin[a]		X	X	X	X	X	X	X	X				
Cycle of Chemotherapy		X	X	X	X	X	X	X	X				
Informed consent [b]	X												
Informed consent for future biomedical research [b]	X												
History/Demographics [c]	X												
Physical exam [d]	X	X	X	X	X	X	X	X	X	X	X	X	X
Concomitant meds [e]	X	X	X	X	X	X	X	X	X	X	X		
ECOG - Performance status [f]	X	X	X	X	X	X	X	X	X	X	X		

Vital signs, Weight [g]	X	X (when clinically indicated)								x	x		
Adverse event evaluation [h]	X	X	X	X	X	X	X	X	X	X	X		
Tumor sample collection [i]	X												
Hematology [j]	X	X	X	X	X	X	X	X	X	X	X	X	X
Serum chemistry [j]	X	X	X	X	X	X	X	X	X	X	X	X	X
TSH, T3, FT4 [j]	X		X		X		X			X	X		
Hepatitis testing [j]	X	If clinically indicated, perform test as needed.											
	**Screening phase**	**Treatment cycles [a] (chemotherapy + nivolumab)**						**Maintenance phase Cycles**		**End of treatment**	**Post-treatment**		
											Safety follow up	Follow up visits	Survival follow up [o]
	Pre-Study	1	2	3	4	5	6	1	2 and beyond	Time of treatment discontinuation	30 days after last dose	Every 12 w after last dose	Every 12w
Cytokines [j]	X	Anytime when a suspected cytokine release syndrome occurs, immediately after the AE, and one week after occurrence of the AE											
Optional serum and plasma samples for biomedical research [K]	X									X			
Urinalysis [j]	X												
Serum or urine pregnancy test [l]	X												
EKG[m]	X												
Radiologic evaluation [n]	X	Treatment Period: Every 8 weeks counting from Cycle 1 Day 1 for 12 months, and every 12 weeks thereafter.<br>EOT: If a scan was not conducted within 30 days prior to end of study treatment<br>Efficacy follow-up: Continue same schedule as during treatment period until central review confirmed irRECIST progression.											

a) Unless otherwise specified, assessments/procedures are to be performed on Day 1 and prior to the first dose of treatment for each cycle.
b) Informed consent must be obtained before conducting any study-specific procedures (i.e. all of the procedures described in the protocol). The process of obtaining informed consent should be documented in the patient source documents. If a patient decides not to participate in the optional biomarker study, this in no way will affect the patient's ability to participate in the main research study.
c) All active conditions, and any condition diagnosed within the prior 10 years that are considered to be clinically significant by the Investigator. Any autoimmune disorders, regardless of onset date, should be recorded
d) Complete physical exam during the screening period. Clinically significant abnormal findings should be recorded as medical history. After the first dose of trial treatment new clinically significant abnormal findings should be recorded as AEs.
e) Site staff will review and record prior medication taken by the subject within 30 days of first dose.All concomitant medications received during the trial through 30 days after the last dose of trial treatment should be recorded.
f) ECOG status should be measured and registered at patient records and eCRF at screening, prior to dosing on day 1 of each treatment cycle and at discontinuation of trial treatment.
g) Vital signs should include temperature, pulse, respiratory rate, weight and blood pressure. Height will be measured at screening only.
h) Adverse events will be graded and recorded throughout the trial and during the follow-up period according to NCI CTCAE Version 5.0. Toxicities will be characterized in terms regarding seriousness, causality, toxicity grading, and action taken with regard to trial treatment.
i) Tumor tissue sample is mandatory to participate in this trial. It is accepted either pre-existing archived or newly-obtained (fresh tissue) biopsy specimens from either primary or metastatic tumor, whichever is most recent.
j) Laboratory tests for screening should be performed within 7 days prior to study enrollment. If performed < 7 days prior to the first dose of trial therapy the screening laboratory tests will serve as cycle 1 day 1 laboratory tests. If not, laboratory tests will need to be performed again on day 1 of cycle 1. Biochemistry includes: Albumin, Alkaline phosphatase, ALT, AST, Gamma-glutamyl-transferase (GGT), Lactate Dehydrogenase (LDH), Calcium, Magnesium, Phosphorus, Sodium, Potassium, Creatinine, Creatine kinase, Direct Bilirubin, Indirect Bilirubin, Total Bilirubin, Total Protein, Blood Urea Nitrogen (BUN) or Urea, Uric Acid, Amylase, Lipase, Glucose. The main laboratory determinations are summarized in Table 7 of the protocol.
k) Serum and plasma samples for optional biomarker studies will be collected prior to the beginning of treatment and at end of treatment.
l) Serum/Urine β-hCG A serum pregnancy test must be performed at screening (at the local laboratory) and when clinically indicated.
m) A standard 12-Lead Electrocardiogram (ECG) using local procedures at screening, additional ECGs will be performed only if clinically indicated.
n) The preferred radiologic technique is CT with intravenous (IV) contrast. If a patient is known to have a contraindication to CT contrast or develops a contraindication during the trial, a non-contrast CT of the chest (MRI is not recommended due to respiratory artifacts) plus a contrast-enhanced MRI (if possible) of the abdomen and pelvis should be performed. Brain CT scan or MRI or Whole Body Bone Scan are not required neither at baseline nor in the follow-up in the absence of neurological/bone signs or symptoms. Participants who discontinue study treatment in absence of disease progression, must continue their follow-up every 12 weeks (+/-7 days) by imaging until disease progression.

o) In this study, OS is a key endpoint of the study. Post-study follow-up is of critical importance and is essential. Participants who discontinue study treatment must continue their follow-up every 12 weeks (+/- 7 days), until death or the conclusion of the study (phone contacts are allowed).

8. Evaluation criteria

8.1. Definitions

- Evaluable for toxicity: All patients will be evaluable for toxicity from the time of their first treatment with nivolumab in combination with platinum-doublet chemotherapy.
- Evaluable for response: All eligible patients will be included in the response rate calculation. The subset that will be assigned a response category (Complete Response, Partial Response, Stable Disease or Progressive Disease; see definitions below) are all patients who have received at least one treatment and have their disease re-evaluated. Patients will have their response classified according to the definitions set out below *(Eisenhauer E, 2009)*.

8.2. Evaluation of efficacy

- Study evaluations will take place in accordance with the Schedule of Activities described in Section 7.4.
- Objective tumor response and time of progression will be measured according to RECIST criteria v.1.1.
- Response criteria are essentially based on a set of measurable lesions identified at baseline as target lesions, and – together with other lesions that are denoted as non-target lesions – followed until disease progression.

8.2.1. Measurability of tumour lesions at baseline

- Chest- abdominal and pelvic CT scan will be used to measure the lesions according to RECIST criteria.
- To meet screening criteria, initial tumor imaging must be performed within 28 days prior to the beginning of treatment. This scan will be considered the baseline assessment for the study. The site study team must review pre-trial images to confirm the subject has at least one target lesion (i.e. meets measurability requirements) per RECIST 1.1.
- irRECIST criteria will be used to measure ORR as secondary endpoint.

8.2.1.1. Definitions

- Measurable disease: the presence of at least one measurable lesion. If the measurable disease is restricted to a solitary lesion, its neoplastic nature should be confirmed by cytology/histology.
- Measurable lesions: *tumour lesions* that can be accurately measured in at least one dimension (longest diameter to be recorded) as ≥ 20 mm with chest x-ray, and as ≥ 10 mm with CT scan or clinical examination [using calipers]. Bone lesions are considered measurable only if assessed by CT scan and have an identifiable soft tissue component that meets these requirements (soft tissue component ≥ 10 mm by CT scan). *Malignant lymph nodes* must be ≥ 15 mm in the short axis to be considered measurable; only the short axis will be measured and followed. All tumour measurements must be recorded in millimeters (or decimal fractions of centimeters) by use of a ruler or calipers. Tumour lesions situated in a previously irradiated area, or in an area subjected to other locoregional therapy, are usually not considered measurable unless there has been demonstrated progression in the lesion. Provide detail on the conditions under which such lesions would be considered measurable.

- Non-measurable lesions: all other lesions (or sites of disease), including small lesions are considered non-measurable disease. Bone lesions without a measurable soft tissue component, leptomeningeal disease, ascites, pleural/pericardial effusions, lymphangitis cutis/pulmonis, inflammatory breast disease, lymphangitic involvement of lung or skin and abdominal masses followed by clinical examination are all non-measurable. Nodes that have a short axis <10 mm at baseline are considered non-pathological and should not be recorded or followed.
- Target Lesions: when more than one measurable tumour lesion or malignant lymph node is present at baseline all lesions up to *a maximum of 5 lesions total* (and a maximum of *2 lesions per organ*) representative of all involved organs should be identified as target lesions and will be recorded and measured at baseline. Target lesions should be selected on the basis of their size (lesions with the longest diameter), be representative of all involved organs, but in addition should be those that lend themselves to *reproducible repeated measurements*. Note that pathological nodes must meet the criterion of a short axis of ≥ 15 mm by CT scan and only the *short* axis of these nodes will contribute to the baseline sum. At baseline, the sum of the target lesions (longest diameter of tumour lesions plus short axis of lymph nodes: overall maximum of 5) is to be calculated and recorded.
- Non-target Lesions: all non-measurable lesions (or sites of disease) including pathological nodes (those with short axis ≥ 10 mm but < 15 mm), plus any measurable lesions over and above those listed as target lesions are considered *non-target lesions*. Measurements are not required but these lesions should be noted at baseline and should be followed as "present" or "absent".

8.2.1.2. Methods of measurements

- Patient must have measurable lesions on CT according to RECIST 1.1 in order to enter the study.
- The preferred radiologic technique is CT with IV contrast. If a patient is known to have a contraindication to CT contrast or develops a contraindication during the trial, a non-contrast CT of the chest (MRI is not recommended due to respiratory artifacts) plus a contrast-enhanced MRI (if possible) of the abdomen and pelvis should be performed.

8.2.2. Tumor response evaluation

8.2.2.1. Response evaluation according RECIST 1.1 [xliv]

- All patients will have their best response according to RECIST criteria 1.1 from the start of study treatment until the end of treatment classified as outlined below:
  - Complete or partial responses may be claimed only if the criteria for each are met at a subsequent time point at least 4 weeks later. Refer to the table below *(Table 8.2.2.1-a).*
  - Complete Response (CR): disappearance of all *target* and *non-target* lesions and normalization of tumour markers. Pathological lymph nodes must have short axis measures < 10 mm (Note: continue to record the measurement even if < 10 mm and considered CR). Tumour markers must have normalized. Residual lesions (other than nodes < 10 mm) thought to be non-malignant should be further investigated (by cytology or PET scans) before CR can be accepted.

- Partial Response (PR): at least a 30% decrease in the sum of measures (longest diameter for tumour lesions and short axis measure for nodes) of target lesions, taking as reference the baseline sum of diameters. Non target lesions must be non-PD.
- Stable Disease (SD): Neither sufficient shrinkage to qualify for PR nor sufficient increase to qualify for PD taking as reference the smallest sum of diameters on study.
- Progressive Disease (PD): at least a 20% increase in the sum of diameters of measured lesions taking as references the smallest sum of diameters recorded on study (including baseline) AND an absolute increase of ≥ 5 mm. Appearance of new lesions will also constitute PD (including lesions in previously unassessed areas). In exceptional circumstances, unequivocal progression of non-target disease may be accepted as evidence of disease progression, where the overall tumour burden has increased sufficiently to merit discontinuation of treatment, for example where the tumour burden appears to have increased by at least 73% in volume (which is the increase in volume when all dimensions of a single lesion increase by 20%). Modest increases in the size of one or more non-target lesions are NOT considered unequivocal progression. If the evidence of PD is equivocal (target or non-target), treatment may continue until the next assessment, but on further documentation, the earlier date must be used [xlv].

Table 8.2.2.1-a: Integration of target, non-target and new lesions into response assessment:

**Target Lesions**	**Non-Target Lesions**	**New Lesions**	**Overall Response**	**Best Response for this category also requires**
Patients with Target lesions ± non target lesions				
CR	CR	No	CR	Normalization of tumour markers, tumour nodes < 10 mm
CR	Non-CR/Non-PD	No	PR	
CR	Not all evaluated	No	PR	
PR	Non-PD/ not all evaluated	No	PR	
SD	Non-PD/ not all evaluated	No	SD	Documented at least once ≥ **4 weeks from baseline [note, protocol may define; 6-8 weeks is recommended]**
Not all evaluated	Non-PD	No	NE	
PD	Any	Any	PD	
Any	PD	Any	PD	
Any	Any	Yes	PD	
Patients with Non target lesions ONLY				
No Target	CR	No	CR	Normalization of tumour markers, all tumour nodes <10 mm
	Non-CR/Non-PD	No	Non-CR/Non-PD	
	Not all evaluated	No	NE	
	Unequivocal PD	Any	PD	
	Any	Yes	PD	

Note: Patients with a global deterioration of health status requiring discontinuation of treatment without objective evidence of disease progression [or evidence of unequivocal disease progression] at that time should be reported as "*symptomatic deterioration*". This is a reason for stopping therapy, but is NOT objective PD. Every effort should be made to document the objective progression even after discontinuation of treatment.

The best overall response can be interpreted as below *(Table 8.2.2.1-b)*:

Table 8.2.2.1-b. Response on confirmation

**Response: First time point**	**Subsequent time point**	**BEST overall response**	**Also requires**
CR	CR	CR	Normalization of tumour markers, tumour nodes < 10 mm
CR	PR	SD, PD or PR (see comment*)	-
CR	SD	SD provided minimum criteria for SD duration met, otherwise, PD	-
CR	PD	SD provided minimum criteria for SD duration met, otherwise, PD	-
CR	NE	SD provided minimum criteria for SD duration met, otherwise NE	-
PR	CR	PR	-
PR	PR	PR	-
PR	SD	SD	-
PR	PD	SD provided minimum criteria for SD duration met, otherwise, PD	-
PR	NE	SD provide minimum criteria for SD duration met, otherwise, NE	-
NE	NE	NE	-
* May consider PR providing initial "CR" likely PR on subsequent review – then original CR should be corrected. Recurrence of lesion after true CR is PD.			

8.2.2.2. Response evaluation according irRECIST [xlvi]

- Following radiological assessment of PD by RECIST 1.1, sites will assess tumor response and progression per immune-related RECIST (irRECIST) for all subjects included in the trial.

- If imaging shows PD per RECIST 1.1, tumor assessment may be repeated at the site at least 4 weeks later to confirm PD (per irRECIST) with the option of continuing treatment until this scan is obtained for clinically stable subjects (see Table 11). Subjects who have unconfirmed disease progression (per irRECIST) may continue on treatment and follow the regular imaging schedule intervals until documented disease progression, the start of new anti-cancer treatment, withdrawal of consent, death, or the end of the trial, whichever occurs first.
- In determining whether or not the tumor burden has increased or decreased, investigators should consider all target lesions as well as non-target lesions.
- Subjects that are deemed clinically unstable are not required to have repeat imaging for confirmation. If radiologic progression is confirmed by subsequent scan, it is recommended that the subject be discontinued from trial treatment unless, in the investigator's opinion, the subject is deriving benefit from treatment. Clinically stable subjects may continue to receive trial therapy. Clinically stable is defined as follows:
    - Absence of signs and symptoms indicating disease progression.
    - No decline in ECOG performance status.
    - Absence of rapid progression of disease.
    - Absence of progressive tumor at critical anatomical sites requiring urgent alternative medical intervention.

Table 8.2.2.2. Management after 1st radiological progression

	**Clinically stable**		**Clinically unstable**	
	**Imaging**	**Treatment**	**Imaging**	**Treatment**
1st Radiological evidence of PD per RECIST 1.1	Repeat imaging at ≥ 4 weeks to confirm PD.	May continue treatment at investigator's discretion while waiting for confirmatory scan.	Repeat imaging at ≥ 4 weeks to confirm PD if possible.	Discontinue treatment.
Repeat scan confirms PD	No additional imaging required	Discontinue treatment.	No additional imaging required	
Repeat scan shows SD, PR or CR.	Continue regularly schedule imaging every 8 weeks.	Continue study treatment at investigator's discretion.	Continue regularly schedule imaging every 8 weeks.	May restart study treatment as per investigator´s discretion.

8.2.2.3. Frequency of tumor re-evaluation

- The first on-study imaging assessment should be performed at 12 weeks (± 7 days) after the first dose of trial treatment. Subsequent imaging should be performed every 12 weeks (± 7 days) until PD.
- Imaging should be repeated at least 4 weeks after the first observation of a complete or partial response.
- Imaging may be more frequent if clinically indicated. Imaging should not be delayed for delays in cycle starts.

- After discontinuation of protocol treatment, patients who have not progressed will still be re-evaluated every 12 weeks.

8.2.3. Reporting of tumor response

All patients included in the study must be assessed for response to treatment, even if there is a major protocol treatment deviation or if they are ineligible, or not followed/re-evaluated. Each patient will be assigned one of the following categories: complete response, partial response, stable disease, progressive disease, early death from malignant disease, early death from toxicity, early death from other cause or unknown (not assessable, insufficient data).

Early death is defined as any death occurring before the first per protocol time point of tumor re-evaluation. The responsible investigator will decide if the cause of death is malignant disease, toxicity or other cause.

Patients for whom response is not confirmed will be classified as "unknown", unless they meet the criteria for stable disease (or the criteria for partial response in case of an unconfirmed complete response). Patients' response will also be classified as "unknown" if insufficient data were collected to allow evaluation per these criteria. Please refer to table *8.2.2.1-b.*

8.2.3.1. Response duration

Response duration will be measured from the time measurement criteria for CR/PR (whichever is first recorded) are first met until the first date that recurrent or progressive disease is objectively documented.

8.2.3.2. Stable disease duration

Stable disease duration will be measured from the time of start of treatment (or randomization for randomized studies) until the criteria for progression are met.

8.2.3.3. Progression Free Survival

Progression free survival will be calculated from the date treatment with first-line chemotherapy and nivolumab begins until the date of first progressive disease as per RECIST 1.1 or death.

8.2.3.4. Overall Survival

Overall survival will be calculated from the date treatment with first-line chemotherapy and nivolumab begins until the date of death from any cause.

9. Statistical considerations

9.1. Statistical design

9.1.1. Sample Size Determination

This is a phase II open-label, single arm study. The primary endpoint is the one-year overall survival rate.

The sample size of this study is driven by its primary objective which is to determine the one-year OS rate calculated from the date treatment with nivolumab and platinum-based chemotherapy begins, until the date of death from any cause in patients with advanced G3 NENs of GEP tract or unknown primary site, who have not received any prior antineoplastic systemic treatment.

Based on data derived from tumor registries[xlvii],[xlviii], median OS for these patients is approximately 11-12 months, with 1-year overall survival rate of 50%. The primary hypothesis (H1) is that chemotherapy in combination with nivolumab is superior to chemotherapy in terms of OS, achieving a median OS of 14 months, with 72% patients alive at 12 months. The study will be considered successful if the primary hypothesis is statistically significant. Considering the following assumptions: a power of 80%, alpha error of 0.05, 18 months accrual time and 12-month follow-up period, a total number of 38 patients should be included in the study, considering a drop out rate of 10%.

9.2. Populations for Analysis

For purpose of analysis, the following populations are defined:

Population	Description
Enrolled	All participants who signed informed consent and were registered.
Treated	All participants who received at least one dose of any study medication. This is the primary dataset for dosing and safety analysis.
Response-Evaluable	All treated subjects who have a baseline and at least one on-treatment imaging evaluation or had progression or death prior to the first on-treatment scan.

9.3. Statistical Analysis

9.3.1. Efficacy Analysis

Endpoint	Statistical Analysis Methods
Primary	Primary endpoint is one year OS rate with nivolumab plus chemotherapy. This is defined as the proportion of patients that remain alive at 12 months since the beginning of treatment. OS will be censored on the last date a participant was known to be alive.

Secondary	PFS and OS with nivolumab plus chemotherapy assessed by Central Review.<br>ORR with nivolumab plus chemotherapy assessed by Central Review.<br>Chromogranin A and enolase levels and their association with ORR, PFS,<br>and OS.<br>**ORR** is defined as the number of participants with a best overall response of confirmed CR or PR divided by the number of enrolled participants. BOR is defined as the best response designation, as determined by the central review, recorded between the date of treatment start and the date of objectively documented progression per RECIST 1.1 or, the date of initiation of subsequent anti-cancer therapy, whichever occurs first. For participants without documented<br>progression or subsequent anti-cancer therapy, all available response designations will contribute to the BOR determination. For participants who continue treatment beyond progression, the BOR will be determined based on response designations recorded up to the time of the initial RECIST 1.1-defined progression. Complete or partial responses may be claimed only if the criteria for each are met at a subsequent time point of ≥ 4 weeks later.

9.3.1.1. Methods for Primary Endpoint

The efficacy analysis will be performed for the treatment population.

For the primary endpoint, the OS rate at 12 months with 95% CIs will be estimated using Kaplan-Meier methodology if follow-up requirement is met. OS curves, medians with 95% CIs and OS rates at 6, 18 and 24 months with 95% CIs will be estimated if follow-up requirement is met. The proportion will be calculated by the product limit method (Kaplan-Meier [K-M] estimate), which takes into account censored data.

9.3.1.2. Methods for Secondary Endpoints

The efficacy analysis for secondary endpoints will be performed for all treated patients.

PFS is defined as the time from the randomization date to the date of the first documented tumor progression per RECIST 1.1, or death due to any cause.

Participants who did not progress or die will be censored on the date of their last evaluable tumor assessment. Participants who did not have any on study tumor assessments will be censored on the beginning of treatment date. PFS curves, PFS medians with 95% CIs, and PFS rates at 6, 12, 18, 24, 36, and 48 months with 95% CIs will be estimated using Kaplan-Meier methodology if follow-up requirements are met.

ORR is defined as the number of participants with a best overall response (BOR) of confirmed CR or PR divided by the number of enrolled participants. BOR is defined as the best response designation, as determined by central review, recorded between the date of treatment start and the date of objectively documented progression per RECIST 1.1 or, the date of initiation of subsequent anti-cancer therapy, whichever occurs first. For participants without documented progression or subsequent anti-cancer therapy, all available response designations will contribute to the BOR determination. For participants who continue treatment beyond progression, the BOR will be determined based on response designations recorded up to the time of the initial RECIST 1.1-defined progression. Complete or partial responses may be claimed only if the criteria for each are met at a subsequent time point of ≥ 4 weeks later.
An estimate of the response rate and an associated exact two-sided 95% CI will be presented.

9.3.2. Safety Analysis

Safety analysis will be performed in all treated participants.

Descriptive statistics of safety will be presented using National Cancer Institute (NCI) Common Terminology Criteria for Adverse Events (CTCAE) version 5.0. All on-study AEs, drug related AEs, SAEs and drug-related SAEs, AEs and drug-related AEs leading to drug discontinuation will be tabulated using worst grade per NCI CTCAE v 5.0 criteria by system organ class and preferred term, based on MedDRA terminology. On-study lab parameters including hematology, coagulation, chemistry, liver function and renal function will be summarized using worst grade per NCI CTCAE v 5.0 criteria.

A tabular summary of the incidence of overall immune-mediated adverse events (imAEs) and serious imAEs will be performed. Frequency, management and resolution of imAEs will be analyzed and a descriptive analysis including time-to-onset, severity, duration, action taken with the study drug, dosing delays of the study drug, corticosteroid details, re-challenge information and outcome of the AE will be individually characterized by imAE category.

10. Translational research

Baseline tumor samples will be mandatory prior to the beginning of the study. Serial blood samples (including serum and plasma at baseline and at disease progression or EOT) will be collected for additional biomarker research to identify prognostic and/or predictive factors of response to nivolumab and carboplatin-etoposide chemotherapy.

Biological samples may undergo genomic, metabolomic, transcriptomic and proteomic analyses or other molecular assessments related to the disease, the immunological landscape of tumors and patients, or the mechanism of action of drugs tested with this purpose. Additional information on this subject is available for all participants in the informed consent form.

The samples will be sent to and stored at the Gastrointestinal Tumors Research Unit from the Research Institute at the Hospital Universitario 12 de Octubre, Madrid.

11. Patient registration procedure

- Each patient is identified in the study by a Subject Number (Subject No.) that is assigned when the patient is first enrolled for screening and is retained as the primary identifier for the patient throughout his/her entire participation in the trial. The Subject No. consists of the Center Number (Center No.) with a sequential patient number suffixed to it, so that each patient is numbered uniquely across the entire database.
- Upon signing the informed consent form, the patient is assigned to the next sequential Subject No. available to the investigator.
- The investigator or designated staff will contact the IRT and provide the requested identifying information for the patient to register them into the IRT. Once assigned, the Subject No. must not be reused for any other patient and the Subject No. for that individual must not be changed, even if the patient is re-screened. If the patient fails to start treatment for any reason, the reason will be entered into the Screening Disposition page.

12. Reporting adverse events

12.1. Definitions for adverse event reporting

- An **Adverse Event (AE)** is defined as any untoward medical occurrence or experience in a patient or clinical investigation subject which occurs following the administration of the trial medication regardless of the dose or causal relationship. This can include any unfavorable and unintended signs (such as rash or enlarged liver), or symptoms (such as nausea or chest pain), an abnormal laboratory finding (including blood tests, x-rays or scans) or a disease temporarily associated with the use of the protocol treatment. *(ICH-GCP).*
- An **Adverse Drug Reaction (ADR)** is defined as any response to a medical product, that is noxious and/or unexpected, related to any dose. *(ICH-GCP). Response to a medicinal product* (used in the above definition) means that a causal relationship between the medicinal product and the adverse event is at least a reasonable possibility, i.e. the relationship cannot be ruled out.
- An **Unexpected Adverse Drug Reaction** is any adverse reaction for which the nature or severity is not consistent with the applicable product information (e.g., Investigator's Brochure). *(ICH-GCP).*
- A **Serious Adverse Event (SAE)** is defined as any undesirable experience occurring to a patient, whether or not considered related to the protocol treatment. A Serious Adverse Event (SAE) which is considered related to the protocol treatment is defined as a **Serious Adverse Drug Reaction (SADR)**.
- Adverse events and adverse drug reactions which are considered as **serious** are those which result in:
    - Death
    - A life threatening event (i.e. the patient was at immediate risk of death at the time the reaction was observed)
    - hospitalization or prolongation of hospitalization
    - persistent or significant disability/incapacity
    - a congenital anomaly/birth defect
    - any other medically important condition (i.e. important adverse reactions that are not immediately life threatening or do not result in death or hospitalization but may jeopardize the patient or may require intervention to prevent one of the other outcomes listed above)

12.2. Reporting procedure

To ensure patient safety, every SAE, regardless of suspected causality, occurring after the patient has provided informed consent must be reported within 24 hours of knowing of its occurrence and until:

- Suspected SAEs up to Day 150 safety follow-up.
- Non-suspected SAEs up to Day 150 safety follow-up or start of new post treatment antineoplastic medication if administered during the period between the 30-Day safety follow-up and 150-Day safety follow-up, whichever is sooner.
- If a patient starts a post treatment antineoplastic therapy after the 30-day safety follow-up, then only SAEs suspected to be related to study treatment should be collected out to 150 days after discontinuation of treatment. SAEs suspected to be related to nivolumab will continue to be collected beyond the 150-Day safety visit.

- Any additional information for the SAE including complications, progression of the initial SAE, and recurrent episodes must be reported as follow-up to the original episode within 24 hours of the investigator receiving the follow-up information. A SAE occurring at a different time interval or otherwise considered completely unrelated to a previously reported one should be reported separately as a new event.
- Any SAEs experienced after the reporting period described above should only be reported if the investigator suspects a causal relationship to the study treatment.
- Information about all SAEs is collected and recorded on the Serious Adverse Event Report Form; all applicable sections of the form must be completed in order to provide a clinically thorough report. The investigator must assess and record the relationship of each SAE to each specific study treatment (if there is more than one study treatment).
- Each reoccurrence, complication, or progression of the original event should be reported as a follow-up to that event regardless of when it occurs. The follow-up information should describe whether the event has resolved or continues, if and how it was treated, and whether the patient continued or withdrew from study participation.
- Suspected Unexpected Serious Adverse Reactions (SUSARs) will be collected and reported to the competent authorities and relevant ethics committees in accordance with Directive 2001/20/EC or as per national regulatory requirements in participating countries.
- SAEs, whether related or not related to study drug, and pregnancies must be reported to BMS within 24 hours \ 1 Business Day of becoming aware of the event. SAEs must be recorded on either CIOMS, MedWatch, or approved site SAE form.
- Pregnancies must be reported and submitted to MFAR Clinical Research that will act on behalf of GETNE transferring the information to BMS. BMS will perform due diligence follow-up using the BMS Pregnancy Form which the investigator must complete.

  [redacted]
- If only limited information is initially available, follow-up reports are required. (Note: Follow-up SAE reports should include the same investigator term(s) initially reported.).
- If an ongoing SAE changes in its intensity or relationship to study drug or if new information becomes available, a follow-up SAE report should be sent within 24 hours \ 1 Business Day to BMS using the same procedure used for transmitting the initial SAE report.
- All SAEs should be followed to resolution or stabilization.

SAEs, whether related or not related to study drug, and pregnancies must be reported to MFAR Clinical Research that will manage, on behalf GETNE, the communications with BMS within 24 hours \ 1 Business Day of becoming aware of the event. SAEs must be recorded on either CIOMS, MedWatch, or approved site SAE form.

The causal relationship to study drug is determined by a physician and should be used to assess all adverse events (AE). The casual relationship can be one of the following:

- ***Related:*** There is a reasonable causal relationship between study drug administration and the AE.
- ***Not related:*** There is not a reasonable causal relationship between study drug administration and the AE.

The causal relationship to study drug is determined by a physician and should be used to assess all adverse events (AE). The casual relationship can be one of the following:

- ***Related:*** There is a reasonable causal relationship between study drug administration and the AE.
- ***Not related:*** There is not a reasonable causal relationship between study drug administration and the AE.
- The term "reasonable causal relationship" means there is evidence to suggest a causal relationship.
- Adverse events can be spontaneously reported or elicited during open-ended questioning, examination, or evaluation of a subject. (In order to prevent reporting bias, subjects should not be questioned regarding the specific occurrence of one or more AEs.)

NON SERIOUS ADVERSE EVENT

Non-serious Adverse Events (AE) are to be provided to BMS in aggregate via interim or final study reports as specified in the agreement or, if a regulatory requirement [eg, IND US trial] as part of an annual reporting requirement.

The Sponsor will reconcile the clinical database AE cases (case level only) transmitted to BMS Global Pharmacovigilance [redacted]

Non-serious Adverse Event Collection and Reporting

The collection of non-serious AE information should begin following the subject's written consent to participate in the study. All non serious adverse events (not only those deemed to be treatment-related) should be collected continuously during the treatment period and for a minimum of (provide # of days depending on the asset and study type) days following the last dose of study treatment.

Non-serious AEs should be followed to resolution or stabilization, or reported as SAEs if they become serious. Follow-up is also required for non-serious AEs that cause interruption or discontinuation of study drug and for those present at the end of study treatment as appropriate.

Laboratory Test Abnormalities

All laboratory test results captured as part of the study should be recorded following institutional procedures. Test results that constitute SAEs should be documented and reported to BMS as such.

The following laboratory abnormalities should be documented and reported appropriately:

- any laboratory test result that is clinically significant or meets the definition of an SAE.
- any laboratory abnormality that required the participant to have study drug discontinued or interrupted.
- any laboratory abnormality that required the subject to receive specific corrective therapy.

It is expected that wherever possible, the clinical rather than laboratory term would be used by the reporting investigator (eg, anemia versus low hemoglobin value).

Potential Drug Induced Liver Injury (DILI) Language required as noted below:

Definition of DILI criteria is mandatory for all pre-marketed asset protocols enrolling participants without known abnormalities in liver function at baseline AND for protocols involving participants with known liver abnormalities at baseline or with other clinical confounders where asset specific criteria for potential drug induced liver injury have been defined. Use for marketed assets is optional.

For protocols without known abnormalities in liver function at baseline, use the mandatory standard DILI definition listed below: Wherever possible, timely confirmation of initial liver related laboratory abnormalities should occur prior to the reporting of a potential DILI event. All occurrences of potential DILIs, meeting the defined criteria, must be reported as SAEs.

Potential drug induced liver injury is defined as:

1. AT (ALT or AST) elevation > 3 times upper limit of normal (ULN) AND
2. Total bilirubin > 2 times ULN, without initial findings of cholestasis (elevated serum alkaline phosphatase) AND
3. No other immediately apparent possible causes of AT elevation and hyperbilirubinemia, including, but not limited to, viral hepatitis, pre-existing chronic or acute liver disease, or the administration of other drug(s) known to be hepatotoxic.

AEs of Special Interest (Product Specific Usually a regulatory requirement. Remove if not applicable)

Pregnancy

If, following initiation of the investigational product, it is subsequently discovered that a study participant is pregnant or may have been pregnant at the time of investigational product exposure, including during at least 5 half-lives after product administration, the investigational product will be permanently discontinued in an appropriate manner (eg, dose tapering if necessary for participant).

The investigator must immediately notify [redacted] of this event and complete one of the following forms within 24 hours of awareness of the event via either the CIOMS, MedWatch or appropriate Pregnancy Surveillance Form in accordance with SAE reporting procedures.

Protocol-required procedures for study discontinuation and follow-up must be performed on the participant.

Follow-up information regarding the course of the pregnancy, including perinatal and neonatal outcome and, where applicable, offspring information must be reported on the CIOMS, MedWatch, BMS Pregnancy Surveillance Form, or approved site SAE form. A BMS Pregnancy Surveillance Form may be provided upon request.

Any pregnancy that occurs in a female partner of a male study participant should be reported to BMS. Information on this pregnancy will be collected on the Pregnancy Surveillance Form. In order for Sponsor or designee to collect any pregnancy surveillance information from the female partner, the female partner must sign an informed consent form for disclosure of this information.

Other Safety Considerations

Any significant worsening noted during interim or final physical examinations, electrocardiograms, X-rays, and any other potential safety assessments, whether or not these procedures are required by the protocol, should also be recorded as a non-serious or serious AE, as appropriate, and reported accordingly.

MFAR Clinical Research on behalf of GETNE will request from BMS GPV&E, [redacted] the SAE reconciliation report and include the BMS protocol number every 3 months and prior to database lock or final data summary.

GPV&E will send the investigator the report to verify and confirm all SAEs have been transmitted to BMS GPV&E.

The data elements listed on the GPV&E reconciliation report will be used for case identification purposes. If the Sponsor determines a case was not transmitted to BMS GPV&E, the case should be sent immediately to BMS [redacted]

13. Quality assurance

13.1. Control of data consistency

The investigator or qualified designee is responsible for recording and verifying the accuracy of subject data.

- The study will use eCRFs. A designated CRO will review the data entered by investigational staff for completeness and accuracy. Electronic data queries stating the nature of the problem and requesting clarification will be created for discrepancies and missing values and sent to the investigational site via the EDC system. Designated investigator site staff is required to respond promptly to queries and to make any necessary changes to the data.
- Concomitant treatments and prior medications entered into the database will be coded using the WHO Drug Reference List, which employs the Anatomical Therapeutic Chemical classification system.
- Medical history/current medical conditions and adverse events will be coded using the Medical dictionary for regulatory activities (MedDRA) terminology.
- The site staff designated by the investigator will enter the information required by the protocol onto the eCRFs as well as onto the designated CRO's requisition form.

13.2. On-site quality control (for multi-centre studies only when data are sent to a Data Centre or similar)

- Before study initiation, at a site initiation visit or at an investigator's meeting, the protocol and CRFs will be reviewed with the investigators and their staff.
- During the study, the field monitor will visit the site regularly to check the completeness of patient records, the accuracy of entries on the CRFs, the adherence to the protocol to Good Clinical Practice, the progress of enrollment, and to ensure that study treatment is being stored, dispensed, and accounted for according to specifications. Key study personnel must be available to assist the field monitor during these visits.
- The investigator must maintain source documents for each patient in the study, consisting of case and visit notes (hospital or clinic medical records) containing demographic and medical information, laboratory data, electrocardiograms, and the results of any other tests or assessments. All information recorded on CRFs must be traceable to source documents in the patient's file. The investigator must also keep the original signed informed consent form (a signed copy is given to the patient).
- The investigator must give the monitor access to all relevant source documents to confirm their consistency with the CRF entries.

13.3. Audits (for multi-centre studies only when data are sent to Data Centre or similar)

- To ensure quality of data, study integrity, and compliance with the protocol and the various applicable regulations and guidelines, the "Sponsor" may conduct site visits to institutions participating to protocols.
- The investigator, by accepting to participate to this protocol, agrees to cooperate fully with any quality assurance visit undertaken by third parties, including representatives from the

“Sponsor”, national and/or foreign regulatory authorities or company supplying the product under investigation, as well as to allow direct access to documentation pertaining to the clinical trial (including CRFs, source documents, hospital patient charts and other study files) to these authorized individuals.

- The investigator must inform the “Sponsor” immediately in case a regulatory authority inspection would be scheduled.

13.4. Central review of pathology

No central pathology review is planned.

13.5 Central review of Images

All images of patients will be uploaded to a platform in DICOM format and will be reviewed centrally by a radiologist specialised in pathology to avoid bias. Information for test uploading and management is provided in a central reviewing manual located at the ISF.

14. Ethical considerations

14.1. Patient protection

This protocol has been written, and the study will be conducted according to the ICH Harmonized Tripartite Guideline for Good Clinical Practice (ref: http://www.ifpma.org/pdfifpma/e6.pdf) with applicable local regulations (including European Directive 2001/20/EC and US Code of Federal Regulations Title 21), and with the ethical principles laid down in the Declaration of Helsinki.

The protocol and the proposed informed consent form must be reviewed and approved by a properly constituted Institutional Review Board/Independent Ethics Committee/Research required to sign a protocol signature page confirming his/her agreement to conduct the study in accordance with these documents and all of the instructions and procedures found in this protocol and to give access to all relevant data and records to auditors, Quality Assurance representatives, IRBs/IECs/REBs and regulatory authorities as required.

14.2. Subject identification

- The investigator must ensure anonymity of the patients; patients must not be identified by names in any documents submitted to the Sponsor.
- Signed informed consent forms and patient enrollment log must be kept strictly confidential to enable patient identification at the site.
- The investigator agrees that the IRB/ERC, or regulatory authority representatives may consult and/or copy trial documents in order to verify worksheet/case report form data. By signing the consent form, the subject agrees to this process. If trial documents will be photocopied during the process of verifying worksheet/case report form information, the subject will be identified by unique code only; full names/initials will be masked prior to transmission.
- By signing this protocol, the investigator agrees to treat all subject data used and disclosed in connection with this trial in accordance with all applicable privacy laws, rules and regulations.

14.3. Informed consent

- Eligible patients may only be included in the study after providing written (witnessed, where required by law or regulation), IRB/IEC/REB-approved informed consent.
- Informed consent must be obtained before conducting any study-specific procedures (i.e. all of the procedures described in the protocol). The process of obtaining informed consent should be documented in the patient source documents.
- The date when a patient’s Informed Consent was actually obtained will be captured in their CRFs.
- An informed consent form (ICF) that is considered appropriate for this study and complies with the ICH GCP guideline and regulatory requirements will be provided with this protocol.
- Women of childbearing potential should be informed that taking the study medication may involve unknown risks to the fetus if pregnancy were to occur during the study and agree that in order to participate in the study they must adhere to the contraception requirement for the duration of the study. If there is any question that the patient will not reliably comply, they should not be entered in the study.

Additional consent form

Sub-studies and studies with an optional biomarker component may have a separate consent form covering those studies. This form will be adapted for each study. These informed consent forms will be submitted for ethical approval together with the Study Protocol and the main informed consent form of the study. If a patient opts not to participate in the optional assessments, this in no way will affect the patient’s ability to participate in the main research study.

15. Publication policy

This trial is intended for publication, even if terminated prematurely. Publication may include any or all of the following: posting of a synopsis online, abstract and/or presentation at a scientific conference, or publication of a full manuscript.

The authors will work to submit a manuscript describing trial results within 12 months after the last data become available.

A synopsis of trial results for approved products will be posted on www.clinicaltrials.gov by 12 months after the last subject's last visit for the primary outcome, 12 months after the decision to discontinue development, or product marketing (dispensed, administered, delivered or promoted), whichever is later.

These timelines may be extended, if additional time is needed for analysis, to protect intellectual property, or to comply with confidentiality agreements with other parties.

Authors of the primary results manuscript will be provided the complete results from the Clinical Study Report, subject to the confidentiality agreement.

When a manuscript is submitted to a biomedical journal the protocol and statistical analysis plan will also be submitted to facilitate the peer and editorial review of the manuscript. If the manuscript is subsequently accepted for publication the journal will be allowed, to post on its website the key sections of the protocol that are relevant to evaluating the trial, specifically those sections describing the trial objectives and hypotheses, the subject inclusion and exclusion criteria, the trial design and procedures, the efficacy and safety measures, the statistical analysis plan, and any amendments relating to those sections.

The first author is responsible for defending the integrity of the data, method(s) of data analysis and the scientific content of the manuscript.

APPENDICES

Appendix A. Performance Status Criteria

**WHO Performance Status Scale**		**Karnofsky Performance Scale**	
**Grade**	Descriptions	**Percent**	Description
0	Normal activity. Fully active, able to carry on all pre-disease performance without restriction.	100	Normal, no complaints, no evidence of disease.
		90	Able to carry on normal activity; minor signs or symptoms of disease.
1	Symptoms, but ambulatory. Restricted in physically strenuous activity, but ambulatory and able to carry out work of a light or sedentary nature (e.g., light housework, office work)	80	Normal activity with effort; some signs or symptoms of disease.
		70	Cares for self, unable to carry on normal activity or to do active work.
2	In bed <50% of the time. Ambulatory and capable of all self-care, but unable to carry out any work activities. Up and about more than 50% of waking hours.	60	Requires occasional assistance, but is able to care for most of his/her needs.
		50	Requires considerable assistance and frequent medical care.
3	In bed >50% of the time. Capable of only limited self-care, confined in bed or chair more than 50% of waking hours.	40	Disabled, requires special care and assistance.
		30	Severely disabled, hospitalization indicated. Death not imminent.
4	100% bedridden. Completely disabled. Cannot carry on any self-care. Totally confined to bed or chair	20	Very sick, hospitalization indicated. Death not imminent.
		10	Moribund, fatal processes progressing rapidly.
5	Deceased.	0	Deceased.

Appendix B. References

[i] Garcia-Carbonero R , Capdevila J, Crespo-Herrero G et al. Incidence, patterns of care and prognostic factors for outcome of gastroenteropancreatic neuroendocrine tumors (GEP-NETs): results from the National Cancer Registry of Spain (RGETNE). Ann Oncol. 2010 Sep;21(9):1794-803.

[ii] Yao JC, Hassan M, Phan A, et al. One hundred years after "carcinoid": epidemiology of and prognostic factors for neuroendocrine tumors in 35,825 cases in the United States. J Clin Oncol. 2008 Jun 20;26(18):3063-72

[iii] Rindi G, Arnold R, Bosman FT, et al. Nomenclature and classification of neuroendocrine neoplasms of the digestive system. WHO classification of tumours of the digestive system. 4th ed Lyon: International Agency for Research on cancer (IARC); 2010.

[iv] Sorbye H, Strosberg J, Baudin E, et al. Gastroenteropancreatic high-grade neuroendocrine carcinoma. Cancer 2014;120: 2814-23.

[v] Sorbye H, Welin S, Langer SW et al. Predictive and prognostic factors for treatment and survival in 305 patients with advanced gastrointestinal neuroendocrine carcinoma (WHO G3): the NORDIC NEC study. Ann Oncol, 2013. 24(1): p. 152-60.

[vi] Moertel CG, Kvols LK, O'Connell MJ et al. Treatment of neuroendocrine carcinomas with combined etoposide and cisplatin. Evidence of major therapeutic activity in the anaplastic variants of these neoplasms. Cancer, 1991. 68(2): p. 227-232.

[vii] Mitry E, Baudin E, Ducreux M et al. Treatment of poorly differentiated neuroendocrine tumours with etoposide and cisplatin. Br J Cancer, 1999. 81(8): p. 1351.

[viii] Iwasa S, Morizane C, Okusaka T et al. Cisplatin and etoposide as first-line chemotherapy for poorly differentiated neuroendocrine carcinoma of the hepatobiliary tract and pancreas. Jpn J Clin Oncol, 2010. 40(4): p. 313-318.

[ix] Deutschbein T, Unger N, Yuece A et al. Chemotherapy in patients with progressive, undifferentiated neuroendocrine tumors: a single-center experience. Horm metab Res, 2011. 43(12): p. 838-843.

[x] Patta A, Fakih M. First-line cisplatin plus etoposide in high-grade metastatic neuroendocrine tumors of colon and rectum (MCRC NET): review of 8 cases. Anticancer Research, 2011. 31(3): p. 975-978.

[xi] Hanahan D, Weinberg RA. Hallmarks of cancer: the next generation. Cell 2011 Mar 4;144(5):646-74.

[xii] Pardoll DM. The blockade of immune checkpoints in cancer immunotherapy. Nat Rev Cancer 2012;12 (24):252-264.

[xiii] Borghaei H, Paz-Ares L, Horn L, et al. Nivolumab versus Docetaxel in Advanced Nonsquamous Non-Small-Cell Lung Cancer. N Engl J Med. 2015 Oct 22;373 (17):1627-39.

[xiv] Robert C, Long GV, Brady B, et al. Nivolumab in previously untreated melanoma without BRAF mutation. N Engl J Med. 2015 Jan 22;372 (4):320-30.

[xv] Hamid O, Carvajal RD. Anti-programmed death-1 and anti-programmed death-ligand 1 antibodies in cancer therapy. Expert Opin Biol Ther. 2013 Jun;13(6):847-61.

[xvi] Motzer RJ, Escudier B, McDermott DF, et al. Nivolumab versus Everolimus in Advanced Renal-Cell Carcinoma. N Engl J Med. 2015 Nov 5;373(19):1803-13.

[xvii] Goh G, Walradt T, Markarov V, et al. Mutational landscape of MCPyV-positive and MCPyV-negative merkel cell carcinomas with implications for immunotherapy. Oncotarget. 2015 Dec 7. doi: 10.18632/oncotarget.6494

[xviii] Talmadge JE, Donkor M, Scholar E. Inflammatory cell infiltration of tumors: Jekyll or Hyde. Cancer Metastasis Rev 2007; 26:373-400.

[xix] Hiraoka N. Tumor-infiltrating lymphocytes and hepatocellular carcinoma: molecular biology. Int J Clin Oncol 2010; 15:544-51.

[xx] Lee HE, Chae SW, Lee YJ, et al. Prognostic implications of type and density of tumour-infiltrating lymphocytes in gastric cancer. Br J Cancer 2008;99(10):1704-11.

[xxi] Leffers N, Gooden MJM, de Jong RA, et al. Prognostic significance of tumor-infiltrating T-lymphocytes in primary and metastatic lesions of advanced stage ovarian cancer. Cancer Immunol Immunother 2009; 58:449-59.

[xxii] Wolchok JD, Hoos A, O'Day S, et al. Guidelines for the evaluation of immune therapy activity in solid tumors: immune-related response criteria. Clin Cancer Res. 2009;15:7412-20.

[xxiii] Brahmer JR, Drake CG, Wollner I, et al. Phase I Study of Single-Agent Anti–Programmed Death-1 (MDX-1106) in Refractory Solid Tumors: Safety, Clinical Activity, Pharmacodynamics, and Immunologic Correlates. J Clin Oncol 2010; 28:3167-3175.

[xxiv] Opdivo Prescribing Information,2015.

[xxv] Antonia SJ, Lopez-Martin JA, Bendell J, et al. Nivolumab alone and nivolumab plus ipilimumab in recurrent small-cell lung cancer (CheckMate 032): a multicentre, open-label, phase 1/2 trial. Lancet Oncol. 2016;17(7):883–95.

[xxvi] Hellmann MD, Ott PA, Zugazagoitia J, Ready NE, et al. Nivolumab (nivo) ± ipilimumab (ipi) in advanced small-cell lung cancer (SCLC): first report of a randomized expansion cohort from CheckMate 032. J Clin Oncol. 2017;35(15_suppl):8503.

[xxvii] M. Reck, D. Vicente, T. Ciuleanu et al. Efficacy and safety of nivolumab (nivo) monotherapy versus chemotherapy (chemo) in recurrent small cell lung cancer (SCLC): Results from CheckMate 331. Annals of Oncology (2018) 29 (suppl_10): x39-x43. 10.1093/annonc/mdy511.

[xxviii] https://news.bms.com/press-release/corporatefinancial-news/bristol-myers-squibb-announces-checkmate-451-study-did-not-mee. Press release: Bristol-Myers Squibb Announces CheckMate -451 Study Did Not Meet Primary Endpoint of Overall Survival with Opdivo Plus Yervoy Vs. Placebo as A

Maintenance Therapy in Patients with Extensive-Stage Small Cell Lung Cancer After Completion of First-Line...

[xxix] De Ruysscher D, Pujol JL, Popat S, et al. STIMULI: a randomised open-label phase II trial of consolidation with nivolumab and ipilimumab in limited-stage SCLC after standard of care chemo-radiotherapy conducted by ETOP and IFCT. Ann Oncol. 2016;27(suppl_6):1430TiP-TiP.

[xxx] Nghiem PT, Bhathia S, Lipton EJ et al. PD-1 Blockade with pembrolizumab in advanced Merkel-Cell Carcinoma. N Engl J 2016 Jun 30; 374(26):2542-52.

[xxxi] Kaufman HL, Russell J, Hamid O et al. Avelumab in patients with chemotherapy-refractory metastatic Merkel cell carcinoma: a multicentre, single-group, open-label, phase 2 trial. Lancet Oncol. 2016 Oct;17 (10):1374-1385.

[xxxii] Antonia SJ, Bendell JC, Taylor MH, et al. Phase I/II study of nivolumab with or without ipilimumab for treatment of recurrent small cell lung cancer (SCLC): CA209-032. J Clin Oncol 33, 2015 (suppl; abstr 7503).

[xxxiii] Ott PA, Fernandez ME, Hiret S, et al. Pembrolizumab (MK-3475) in patients (pts) with extensive-stage small cell lung cancer (SCLC): Preliminary safety and efficacy results from KEYNOTE-028. J Clin Oncol 33, 2015 (suppl; abstr 7502).

[xxxiv] Topalian SL, Bhatia S, Hollebecque A et al. Abstract CT074: Non-comparative, open-label, multiple cohort, phase 1/2 study to evaluate nivolumab (NIVO) in patients with virus-associated tumors (CheckMate 358): Efficacy and safety in Merkel cell carcinoma (MCC). Cancer Research 2017; 77: CT074-CT074

[xxxv] Zhang P, Lu M, Li J et al. Efficacy and safety of PD-1 blockade with JS001 in patients with advanced neuroendocrine neoplasms: a non-randomized, open-label, phase 1b trial. Ann Oncol 2018, Volume 29, Issue suppl_8.

[xxxvi] Galluzzi L, Buque A, Kepp O, et al. Immunological effects of conventional chemotherapy and targeted anticancer agents. Cancer Cell 2015; 28: 690–714.

[xxxvii] Apetoh L, Ladoire S, Coukos G, et al. Combining immunotherapy and anticancer agents: the right path to achieve cancer cure? Ann Oncol 2015; 26: 1813–23.

[xxxviii] Rizvi NA, Hellmann MD, Brahmer JR, et al. Nivolumab in Combination With Platinum-Based Doublet Chemotherapy for First-Line Treatment of Advanced Non-Small-Cell Lung Cancer. J Clin Oncol. 2016 Sep 1;34 (25):2969-79.

[xxxix] Gadgeel SM, Stevenson J, Langer CJ, et al. Pembrolizumab (pembro) plus chemotherpay as front-line therapy for advanced NSCLC: KEYNOTE-021 cohorts A-C. Proc Am Soc Clin Oncol 2016; 34: Abstr 9016

[xl] Langer CJ, Gadgeel SM, Borghaei H, et al. Carboplatin and pemetrexed with or without pembrolizumab for advanced, non-squamous non-small-cell lung cancer: a randomised, phase 2 cohort of the open-label KEYNOTE-021 study. Lancet Oncol. 2016 Nov;17(11):1497-1508.

[xli] Camidge R, Liu S V, et al. Atezolizumab (MPDL3280A) Combined with Platinum-Based Chemotherapy in Non-Small Cell Lung Cancer (NSCLC): A Phase Ib Safety and

Efficacy Update. Journal of Thoracic Oncology • Volume 10, Number 9, Supplement 2, page S176, September 2015.

[xlii] Liu SV, Camidge DR, Gettinger SR et al.Long term follow-up of atezolizumab in combination with platinum-based doublet chemotherapy in patients with advanced non-small-cell lung cancer. Eur J Cancer 2018 2018 Sep;101:114-122.

[xliii] Common Terminology Criteria for Adverse Events (CTCAE) versión 5.0 Published: May 28, 2009 (v4.02: Sept. 15, 2009). http://evs.nci.nih.gov/ftp1/CTCAE/CTCAE_4.03_2010-06-14_QuickReference_8.5x11.pdf

[xliv] Eisenhauer E, Therasse P, Bogaerts J et al. New Response Evaluation Criteria in Solid Tumours: Revised RECIST Guideline (Version 1.1). Eur J Cancer. 2009 Jan; 45(2):228-47.

[xlv] Dancey JE, Dodd LE, Ford R, et al. Recommendations for the assessment of progression in randomised cancer treatment trials. Eur J Cancer 2009 Jan;45(2):281–9.

[xlvi] Wolchok JD, Hoos A, O´Day S et al. Guidelines for the evaluation of immune therapy activity in solid tumors: immune response criteria. Clin Cancer Res 2009 De 1; 15 (23): 7412-20.

[xlvii] Garcia-Carbonero R , Capdevila J, Crespo-Herrero G et al. Incidence, patterns of care and prognostic factors for outcome of gastroenteropancreatic neuroendocrine tumors (GEP-NETs): results from the National Cancer Registry of Spain (RGETNE). Ann Oncol. 2010 Sep;21(9):1794-803.

[xlviii] Sorbye H, Welin S, Langer SW et al. Predictive and prognostic factors for treatment and survival in 305 patients with advanced gastrointestinal neuroendocrine carcinoma (WHO G3): the NORDIC NEC study. Ann Oncol, 2013. 24(1): p. 152-60.
